# Increase in 6-pentyl-alpha-pyrone production by *Trichoderma* spp. in liquid medium using in situ removal by adsorbent resin

**DOI:** 10.1007/s13659-026-00650-y

**Published:** 2026-10-19

**Authors:** Alex Maan, Eirian Jones, John Hampton, Travis Glare, Artemio Mendoza-Mendoza

**Affiliations:** 1 Department of Pest Management and Conservation, Lincoln University, Lincoln, Canterbury New Zealand; 2 Department of Agricultural Sciences, Lincoln University, Lincoln, Canterbury New Zealand; 3 Lincoln Agritech, Lincoln, Canterbury New Zealand

**Keywords:** Bioproduction, Product inhibition, Fungi, Amberlite, Biosynthesis, Antimicrobial

## Abstract

The *Trichoderma*-produced secondary metabolite 6-pentyl-alpha-pyrone (6-PP) has demonstrated promise for agricultural applications, but cost-effective bioproduction remains limited by product inhibition during fermentation. This study aimed to evaluate the effect of media type on 6-PP production in *T. atroviride* IMI206040, screen production by nine New Zealand *Trichoderma* strains, and assess Amberlite XAD-4 in situ product removal in five strains to overcome self-inhibition. *T. atroviride* IMI206040 was cultured for 5 days in potato dextrose broth (PDB) or minimal media supplemented with ammonium or nitrate, and 6-PP production was assessed by thin-layer chromatography (TLC). The ten *Trichoderma* strains were cultured for 7 days in PDB and 6-PP production quantified by spectrophotometry. Finally, five strains were cultured in PDB for 14 days with and without Amberlite XAD-4, and 6-PP quantified by spectrophotometry and gas chromatography (GC). In *T. atroviride* IMI206040, PDB supported the highest 6-PP production, while ammonium-based minimal media repressed production, consistent with nitrogen catabolite repression. Screening revealed up to 14.6-fold variation among strains, with T*. gamsii* LU771 and *T. viridescens* LU1369 as the highest producers. Spectrophotometric and GC measurements showed strain-dependent disagreement, indicating GC is more reliable for cross-strain comparisons. Amberlite XAD-4 increased total 6-PP production 3- to 5.2-fold across all five strains, with > 99% of secreted 6-PP sequestered. The highest-producing strains, *T. gamsii* LU771 and *T. atroviride* LU132, achieved titres approaching 500 mg/L. These findings support Amberlite XAD-4 as an effective in situ product removal method for increasing 6-PP bioproduction and identifying strains with high intrinsic biosynthetic capacity in the absence of product inhibition.

**Graphical abstract:**

**Figure:**
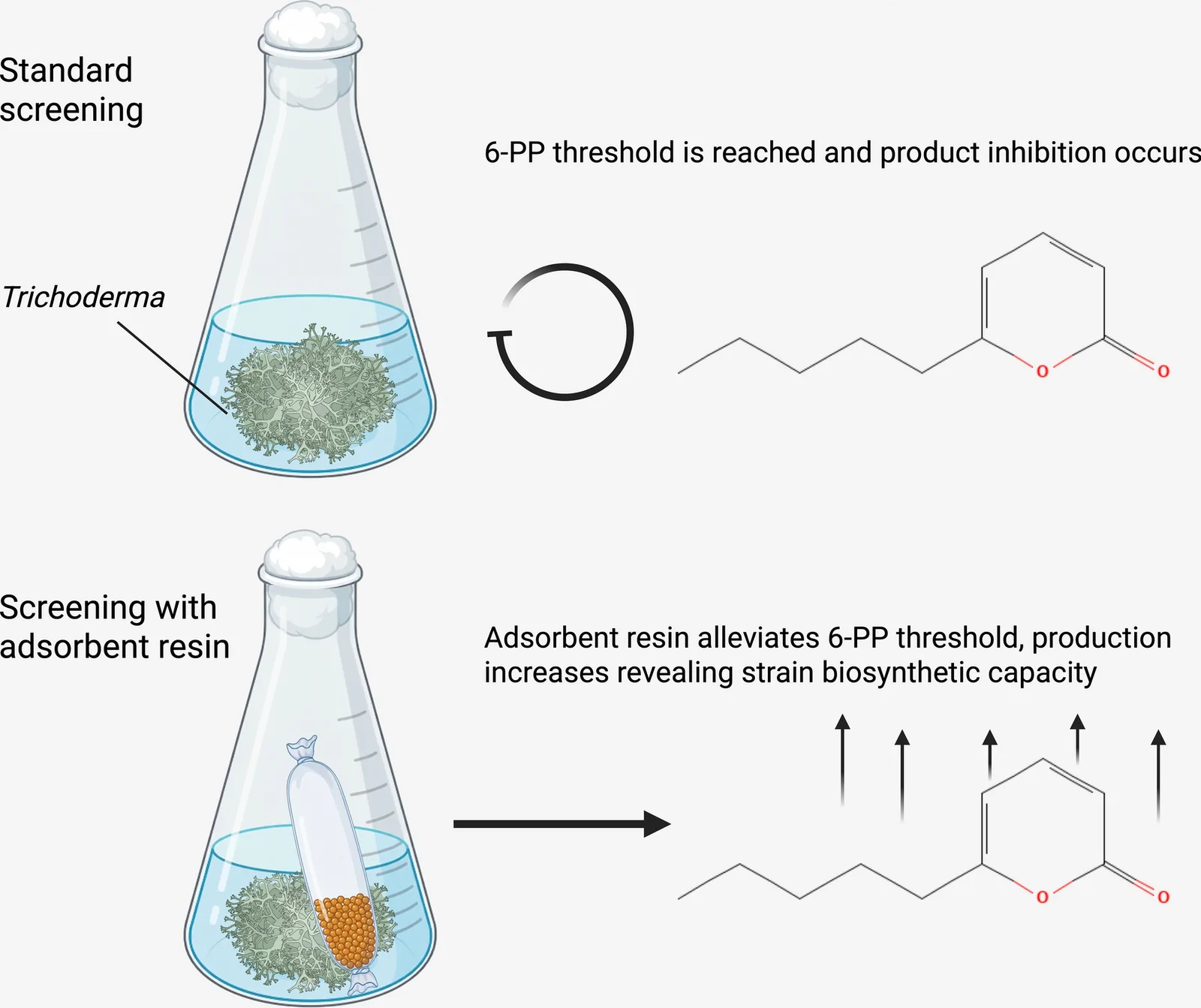

**Supplementary Information:**

The online version contains supplementary material available at https://doi.org/10.1007/s13659-026-00650-y.

## Introduction

The fungal secondary metabolite 6‑pentyl‑alpha‑pyrone (6‑PP) is an unsaturated lactone and volatile organic compound first identified from *Trichoderma viride* by Collins and Halim [1]. It has numerous potential agricultural uses, displaying antimicrobial activity against a number of economically important plant pathogens including inhibition of mycelial growth, spore germination, and mycotoxin production [2–5]. Beyond direct antagonism, 6‑PP can act as a plant defence elicitor, inducing systemic resistance via salicylic acid–dependent signalling, upregulation of pathogenesis-related genes, and enhancement of antioxidant enzyme activity [6–8]. At lower concentrations, 6‑PP also promotes plant growth through an auxin-like effect, stimulating root development, lateral root formation, and biomass accumulation [6, 7, 9, 10]. In addition to these plant-associated effects, 6‑PP has been reported to influence arthropods, for example acting as an attractant for the beneficial parasitoid wasp *Campoletis sonorensis* [11] and exhibiting miticidal activity against *Tetranychus urticae* [12], highlighting its broad potential for agricultural use.

Given the growing need for sustainable alternatives to synthetic agrichemicals, 6‑PP represents a promising eco‑friendly tool for integrated disease and pest management, as well as and plant growth promotion. However, translation of the predominantly laboratory-based evidence into field-scale applications is limited by the challenge of cost‑effective production [13]. Chemical synthesis of 6‑PP has been characterised, and involves multistep reactions, toxic or expensive reagents, and demanding conditions that limit scalability [14, 15]. As an alternative, microbial production using *Trichoderma* spp. offers the prospect of complete de novo synthesis from inexpensive substrates, and numerous fermentation studies have therefore focused on optimising 6‑PP titres [16–20].

Not all *Trichoderma* species are capable of producing 6‑PP, and even within 6‑PP–producing species, substantial strain-to-strain variation is observed [4, 21, 22]. 6‑PP has been isolated from at least thirteen *Trichoderma* species, including *T. atroviride*, *T. gamsii* and *T. viridescens*, with the latter two often emerging as high producers in screening assays [13]. Nevertheless, many strains within these species fail to produce detectable amounts of 6‑PP under standard conditions, and those that do differ in yield [13]. This suggests that strain-level genetics, rather than species identity, largely determines 6‑PP biosynthetic capacity, highlighting the importance of targeted screening and selection. Moreover, various environmental factors during fermentation impact the extent of 6-PP production. For example, light exposure has been identified as a key regulatory factor in *T. atroviride* under liquid fermentation conditions by acting as a repressor and drastically reducing 6-PP production [22, 23], although not all strains appear to be as sensitive to this regulatory pathway [22]. Additionally, media composition influences metabolite production in fungi [24] including 6-PP production in *Trichoderma* [13, 25–27], although the molecular mechanisms by which media composition impacts 6-PP production remain unclear.

A common barrier across 6‑PP–producing *Trichoderma* strains is the self‑inhibitory behaviour of 6‑PP. Although *Trichoderma* is less sensitive than many target fungi, 6‑PP still exerts product inhibition and retards mycelial growth of the producing strain [14, 16, 18–20, 28]. Under liquid fermentation conditions, once a strain-specific maximum tolerated concentration is reached, 6‑PP is actively degraded from the medium, leading to a decline from peak to a lower tolerated level, presumably as a self‑preservation mechanism. This phenomenon is the primary barrier to economically viable bioproduction [19]. To overcome product inhibition, various extractive fermentation and in situ product removal strategies have been evaluated, including dual‑phase culture systems and aqueous two‑phase systems. These sequester 6‑PP away from the aqueous phase containing the mycelia into an organic solvent or polyethylene glycol + salt phase [18, 19, 29]. These approaches can substantially increase 6‑PP production but their efficacy is strongly dependent on biocompatibility with the fungus and often requires sacrificing culture volume or adding process complexity.

A more space-effective in situ 6-PP removal process may be achieved using solid polymeric adsorbents such as Amberlite XAD resins. Adsorbent resins have highly porous structures with high surface area and are capable of binding to/capturing small organic molecules [30]. They have proven to be effective at increasing fungal metabolite production [31], including self-toxic [30, 32] and self-inhibiting [30, 33] products, and have shown excellent compatibility with various fungi [30, 31], including *Trichoderma* [16]. When added concurrently during culture inoculation, Amberlite XAD-2 achieved a 2.75-fold increase (to 248 mg/L) in 6-PP levels in *T. viride* with no apparent negative effects on the fungus [16]. Furthermore, Amberlite resins are regenerable and can be reused, offering an economic approach to in situ product removal at a commercial scale.

The present work aimed to assess the effect of media type on 6-PP production, and combine darkness and Amberlite-assisted in situ removal of 6‑PP during screening of New Zealand native *Trichoderma* isolates, with the goal of identifying high‑producing strains and culture conditions capable of achieving titres relevant to reducing production costs and increasing economic viability for agricultural uses.

## Materials and methods

### *Trichoderma* strains

The *Trichoderma* strains *T. atroviride* IMI206040 (ATCC 20476), *T. atroviride* LU132, *T. atroviride* LU136, *T. atroviride* LU497, *T. atroviride* LU663, *T. atroviride* LU668, *T. gamsii* LU771, *T. gamsii* LU775, *T. gamsii* LU780, and *T. viridescens* LU1369 were used. Strains with LU identifiers are New Zealand isolates from the Lincoln University *Trichoderma* collection. *T. atroviride* IMI206040, isolated in Sweden and deposited in the International Mycological Institute collection, is widely used in other 6-PP studies and used here as a reference strain. To produce fresh conidia, Petri dishes containing 25 mL PDA (BD Difco™) were inoculated, sealed with micropore tape, and incubated at 27 °C for 10–30 days (depending on strain) under a 12 h light cycle (Sanyo versatile environmental test chamber). Conidia were harvested within three days of their production and filtered through a double layer of sterile Miracloth (Millipore, Merck). An improved Double Neubauer Counting Chamber was used to estimate conidial concentrations. Conidial suspensions were refrigerated at 4 °C and used within one month of collection.

### Liquid media and liquid fermentation

Three types of liquid media were used: Potato Dextrose Broth (PDB, BD Difco™) prepared according to the manufacture’s specifications, and minimal media supplemented with NH_4_^+^ (MMNH_4_) or NO_3_^−^ (MMNO_3_) with each 1 L containing 200 mg MgSO_4_.7H_2_O, 900 mg K_2_HPO_4_, 200 mg KCl, 2 mg FeSO_4_.7H_2_O, 2 mg ZnSO_4_.7H_2_O, 1.57 mg MnCl_2_.4H_2_O, 500 mL of pH 5.4 McIlvaine’s buffer [34], 20 g glucose, and either 5.18 g of NH_4_Cl or 8.51 g of KNO_3_ [35]. All experiments were conducted in 250 mL Erlenmeyer flasks containing 70 mL of media and inoculated with 1 × 10^6^ conidia/mL. After inoculation, the flasks were wrapped in aluminium foil to prevent light penetration and incubated in orbital mixer incubators at 160 rpm and 25 °C (Thermo Scientific MaxQ4000 and Ratek orbital mixer incubator; 1.9 cm orbit diameter). All experiments included biological triplicates. After liquid fermentation, *Trichoderma* mycelia were separated from the supernatant via vacuum filtration using Whatman 3MM filter paper.

### Effect of media type on 6-PP production in *T. atroviride* IMI206040

*T. atroviride* IMI206040 was cultured using MMNO_3_, MMNH_4_, and PDB media and incubated for 5 days, after which the supernatant was separated from the mycelia. To create samples for Thin-Layer Chromatography (TLC), 12 mL of filtered supernatant and 3 mL of EA (4:1 supernatant/EA extraction) were combined and vortexed at 3400 rpm for one minute and then centrifuged at 1811 g for 3 min [22]. The solvent phase was recovered and stored in 4 mL analytical vials at − 20 °C. Samples from each biological triplicate were combined in equal portions to create an averaged representation. Fifty μL of each sample was applied to aluminium TLC silica gel 60 _F254_ 20 × 20 plates (Merck). For the 6-PP identification standard, 50 μL of a 200 mg/L 6-PP (Sigma-Aldrich)/ethyl acetate solution was applied. The samples were separated using a glass chamber with 20 mL of *n*-hexane: diethyl ether: acetic acid (70:30:1) as the mobile phase for 45 min. TLC plates were photographed under a 254 nm UV lamp (UVGL-58). Final images are presented in greyscale to increase clarity.

### Comparison of 6-PP production in ten *Trichoderma* strains

All ten *Trichoderma* strains were cultured in PDB and incubated for 7 days as described above. Filtered supernatants were subject to the 4:1 EA extraction process for TLC as described above. The TLC plate was scanned and visualised using a CAMAG HPTLC Scanner 4 and VisionCATS software at 254 and 320 nm wavelengths. For 6-PP quantification by spectrophotometric absorbance, a 1:1 supernatant/EA extract was created whereby 6 mL of filtered supernatant and 6 mL of EA were combined in a 50 mL Falcon tube, vortexed at 3400 rpm, then centrifuged at 1811 g for 3 min and the solvent phase was recovered. To create a standard curve for 6-PP quantification, a stock solution of 150 mg/L 6-PP was prepared using the purified compound in analytical-grade ethyl acetate and used to create dilutions of 1, 2.5, 5, 10, 20, 40, 60, 80, and 100 mg/L [22]. For each dilution, 2 mL was transferred into 10 mm length quartz cuvettes and absorbance was assessed using a UV-1600PC spectrophotometer (VWR®) using a 320 nm wavelength with M. Wave Professional software (v1.0). Following the generation of the standard curve, the absorbance of *Trichoderma* supernatant extracts was assessed, and the cuvettes were washed with ethyl acetate between samples.

### Effect of Amberlite XAD-4 in situ 6-PP extraction on five *Trichoderma* strains

Based on their relative performance in the screening, the highest and lowest producing *T. atroviride* strains (LU497 and LU132, respectively) and *T. gamsii* strains (LU771 and LU775, respectively), as well as a single high-performing *T. viridescens* strain (LU1369), were selected for in situ 6-PP removal using Amberlite XAD-4 (Sigma-Aldrich, hereinafter referred to as Amberlite) placed inside dialysis tubes. The Amberlite was prepared according to Singh et al. [32] by immersing/stirring the resin in twice the volume of methanol for an hour, decanting and rinsing in methanol, followed by five thorough rinses with deionised water, and finally vacuum filtered. The 25 mm MW 1000 dialysis tubing was cut into 15 cm sections and prepared according to the manufacturer’s specifications. Prepared Amberlite XAD-4 was placed inside each dialysis tube in 2 g aliquots with 5 mL of double-distilled water (DDH_2_O), tied off, and autoclaved at 121 °C and 15 psi for 15 min in DDH_2_O. The strains were cultured in flasks containing PDB with dialysis tubes containing 2 g of Amberlite or PDB only for 14 days. Afterwards, 6-PP was extracted from the filtered supernatants using the 1:1 EA process as previously described for quantification by spectrophotometry and gas chromatography (GC). The Amberlite was vacuum filtered to remove the supernatant. To extract 6-PP from the Amberlite, 100 mg samples were weighed, placed into 2 mL tubes with 500 μL of methanol, and vortexed at 3400 rpm for 1 min. The tubes were then placed in an ultrasonic water bath (Bandelin Sonorex Digitec) for 20 min, then vortexed again for 1 min. The methanol phase was transferred to a new 2 mL tube, and the process was repeated twice, resulting in 1.5 mL of methanol-Amberlite extract per sample. The methanol extracts were then dried *in vacuo* using a CentriVap Centrifugal Vacuum Concentrator (Labconco) at 45 °C for 120 min. The dried samples were resuspended in 1 mL of EA and vortexed at 3400 rpm for 1 min to produce the final samples for GC.

### Gas chromatography and gas chromatography coupled to mass spectrometry (GC–MS)

GC quantification of 6-PP was performed using a Shimadzu GC-2010 gas chromatograph equipped with an AOC-20i autosampler and a Restek Rxi-1 ms column (0.25 mm × 30 mm, 25 μm film thickness) using hydrogen as the carrier gas (80 cm/s linear velocity). Sample injection volumes were 1 μL with a 1:10 split ratio, and splitless mode with 2-min sampling for samples with low 6-PP concentration. Samples extracted from Amberlite contained high concentrations of 6-PP, so they were diluted by a factor of 20. For quantification, a standard curve was created using 6-PP diluted in EA. The oven temperature started at 35 °C (2-min hold), then increased by 29 °C/minute to 130 °C (2-min hold), and further by 115 °C/minute to 240 °C, with a 5-min equilibrium between runs. The injector and flame ionisation detector were maintained at 250 °C. The autosampler needle was washed with ethyl acetate between samples. Prior to analysis, the column was conditioned overnight at 35 °C (flame off), then with the flame on for at least two hours before any analysis.

GC–MS analysis was performed using a Shimadzu GCMS-QP2010 gas chromatograph–mass spectrometer equipped with a CTC Combi Pal autosampler ready for liquid injection. Data acquisition was carried out using GCMSolutions version 4.45 software. The chromatographic separation was performed using a Rxi-1 ms 30 m × 0.25 mm i.d. × 0.25 μm film thickness GC column (100% dimethyl polysiloxane—Restek, Bellefonte, PA, USA). Helium was used as the carrier gas at a constant linear velocity of 36.1 cm sec-1. The injection port was held at 250 ⁰C and operated in splitless mode for 100 s before switching to a 20:1 split. Samples in ethyl acetate were liquid-injected (1 μL) and separated on the GC column via a temperature ramp. An initial oven temperature of 40 ⁰C was used, ramped to 320 ⁰C at 10 ⁰C min-1 and then held at this temperature for 10 min. The mass spectrometer’s (MS) ion source and transfer line were set at 260 ⁰C and 320 ⁰C, respectively. The MS was operated in electron-impact (EI) mode at 70 eV. All analyses were carried out in full-scan mode (m/z 33–550), with all VOC peaks integrated for qualitative analysis. All data were compared against the NIST 14 (2014 NIST/EPA/NIH Mass Spectral Library, National Institute of Standards and Technology, Maryland, USA) and Wiley 10 (Wiley Registry of Mass Spectral Data, 10th edition, John Wiley & Sons, New Jersey, USA) mass spectral libraries. Peak identities were confirmed using published retention indices (RI’s) sourced from the NIST Chemistry Webbook website webbook.nist.gov for G1, G2, G9, G38 type (DB-1 ms equivalent) GC columns. An alkane mix (C8-C20, Merck, Australia) was used to calculate the retention indices (RI) of each peak, with identifications confirmed based on their match to published literature RI and mass spectral library matches greater than 80%.

### Statistics

R version 4.4.1 and GraphPad Prism version 10.5.0 for MacOS were used to conduct statistical comparisons and data presentation. Two one-way ANOVAs were conducted to compare 6-PP production and mycelial mass across 10 *Trichoderma* strains, followed by Tukey’s HSD post-hoc comparisons. For the in situ extraction experiment, Bland Altman plots were used to assess measurement disagreement between spectrophotometry and GC for each strain. To assess the effect of Amberlite on 6-PP production, a two-way ANOVA was used, with significant differences in treatment further explored using Tukey’s HSD post-hoc tests.

## Results

### Effect of media type on 6-PP production in *T. atroviride* IMI206040

**Fig. 1:**
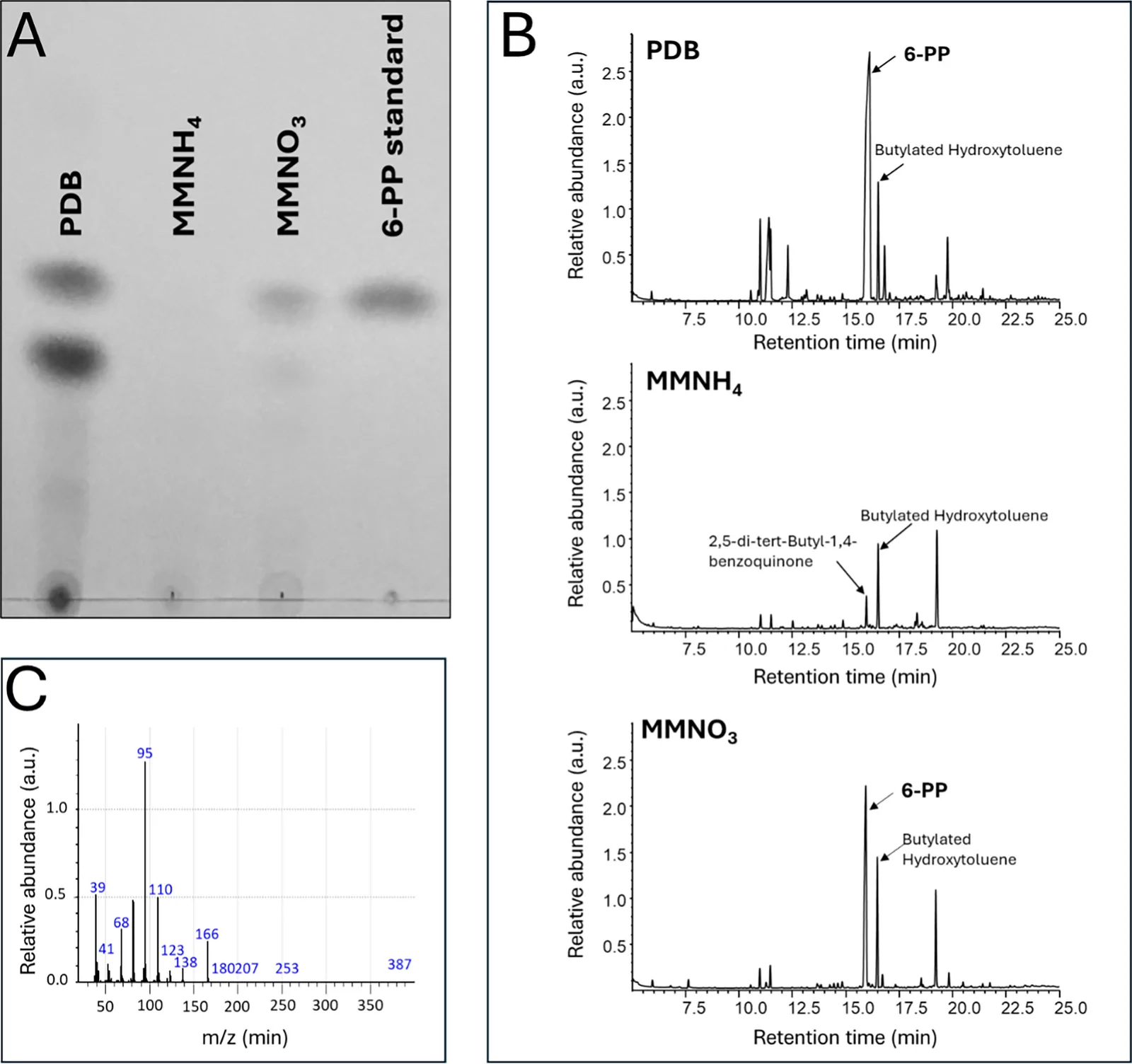
Production of 6-pentyl-alpha-pyrone (6-PP) by *Trichoderma atroviride* IMI206040 in different culture media. **A** Thin-layer chromatography (TLC) analysis with *T. atroviride* IMI206040 supernatant samples cultivated in three different media for 5 days. Lanes from left to right: potato dextrose broth (PDB), minimal media with nitrate (MMNO_3_), minimal media with ammonium (MMNH_4_), 6-PP standard. **B** GC–MS chromatograms of extracts from the different culture media, confirming the presence of a peak corresponding to 6-PP in PDB and MMNO₃ cultures but absent in MMNH_4_. **C** Retention time and mass spectral analysis of the detected peak, showing agreement with the 6-PP standard and confirming the identity of 6-PP

The production of 6-PP by *T. atroviride* differed among the culture media tested. The nutrient-rich medium PDB induced the highest 6‑PP production, followed by the nitrate-based minimal medium (MMNO₃), whereas 6‑PP production was undetectable in the ammonium-based minimal medium (MMNH₄), as shown by TLC (Fig. 1A), and confirmed by GC–MS (Fig. 1B, C). Together, these analyses demonstrate that 6-PP production by *T. atroviride* IMI206040 is strongly influenced by culture medium composition and nitrogen source, with production occurring in PDB and MMNO₃ but not in MMNH₄ under the conditions tested. Moreover, Butylated hydroxytoluene was identified, which has previously been reported to be produced by a diverse range of *Trichoderma* strains and to exhibit antifungal and antibacterial activity [36–38]. In MMNH_4_ medium, accumulation of 2,5-di-tert-butyl-1,4-benzoquinone, a quinone with antifungal activity [39] was detected (Fig. 1B). The presence of 2H-Pyran-2-one, 5,6-dihydro-6-pentyl- was also detected, but in low levels in PDB and not in the other two media (Supplementary Tables 1–3).

### Comparison of 6-PP production in ten *Trichoderma* strains

**Fig. 2:**
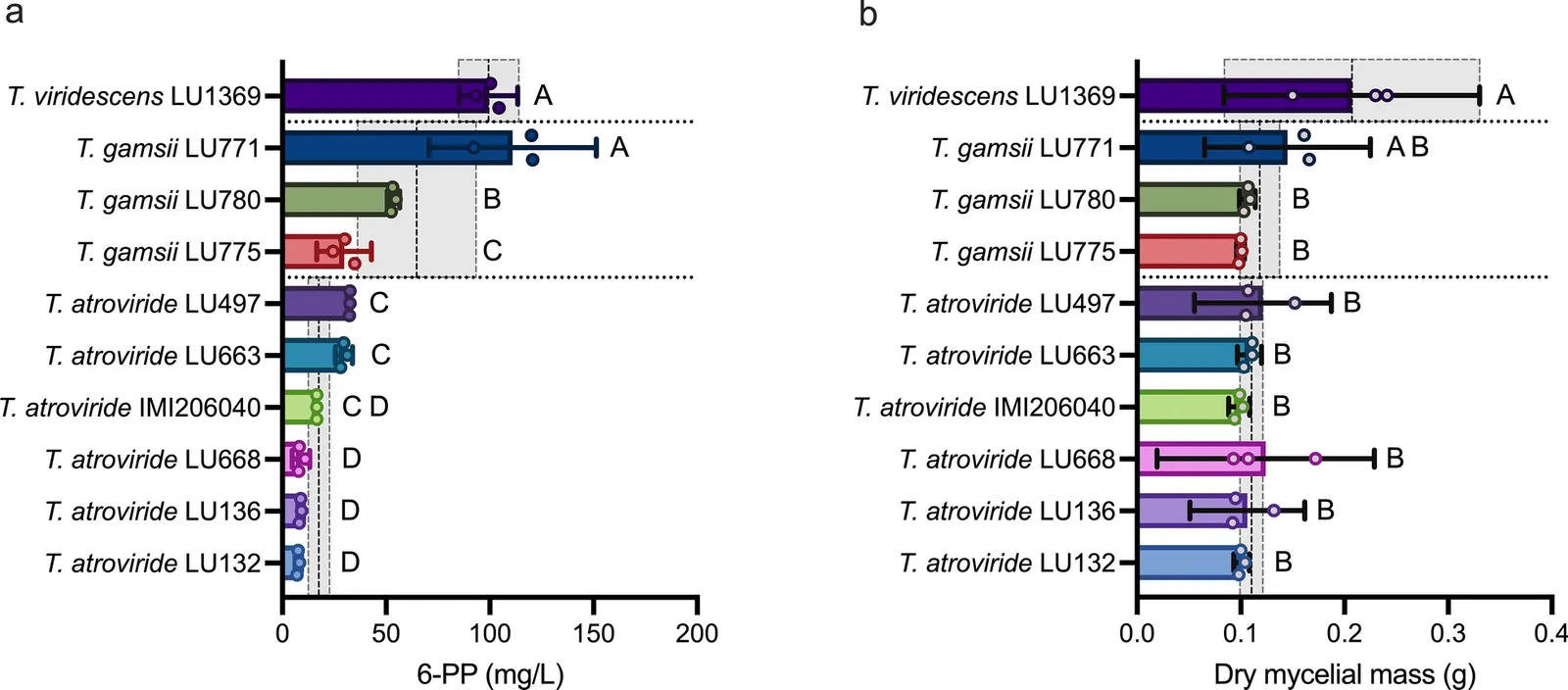
6-PP production and biomass of ten *Trichoderma* strains after 7 days*.* Bar graphs showing **a** 6-PP production from supernatant samples analysed by spectrophotometry at 320 nm, and **b** dry mycelial mass for the same cultures. Each bar represents an individual strain with individual values plotted and error bars representing 95% CI. Strains with the same letter/s are not significantly different from one another. Strains of the same species are grouped as indicated by the horizontal dotted line, with species means indicated by black vertical dashed lines with 95% CI shaded grey

TLC confirmed that all ten *Trichoderma* strains produced 6‑PP and revealed additional strain-specific metabolites absorbing at 254 nm (Supplementary Fig. 1). For spectrophotometric quantification at 320 nm 6‑PP production differed significantly among strains (F(9, 20) = 125.9, *p* < 0.0001). Post hoc comparisons grouped strains into four production classes: the highest producers were *T. viridescens* LU1369 and *T. gamsii* LU771; intermediate producers were *T. gamsii* LU780, *T. gamsii* LU775, *T. atroviride* LU497, *T. atroviride* LU663, and *T. atroviride* IMI206040; and the lowest producers were *T. atroviride* LU668, LU136, and LU132. The top-performing strain, *T. gamsii* LU771, produced 12.7–14.6 times more 6‑PP than the lowest-producing strain (*p* < 0.0001) (Fig. 2a). Mycelial biomass also differed significantly among strains (F(9, 20) = 5.07, *p* = 0.0012), but only *T. viridescens* LU1369 consistently showed higher biomass than most other strains, suggesting a strain-specific trait unrelated to 6‑PP production-biomass relationship (Fig. 2b).

### Comparison of 6-PP production in five *Trichoderma* strains after 14-days

#### Comparison of spectrophotometry and gas chromatography quantification of 6-PP

**Fig. 3:**
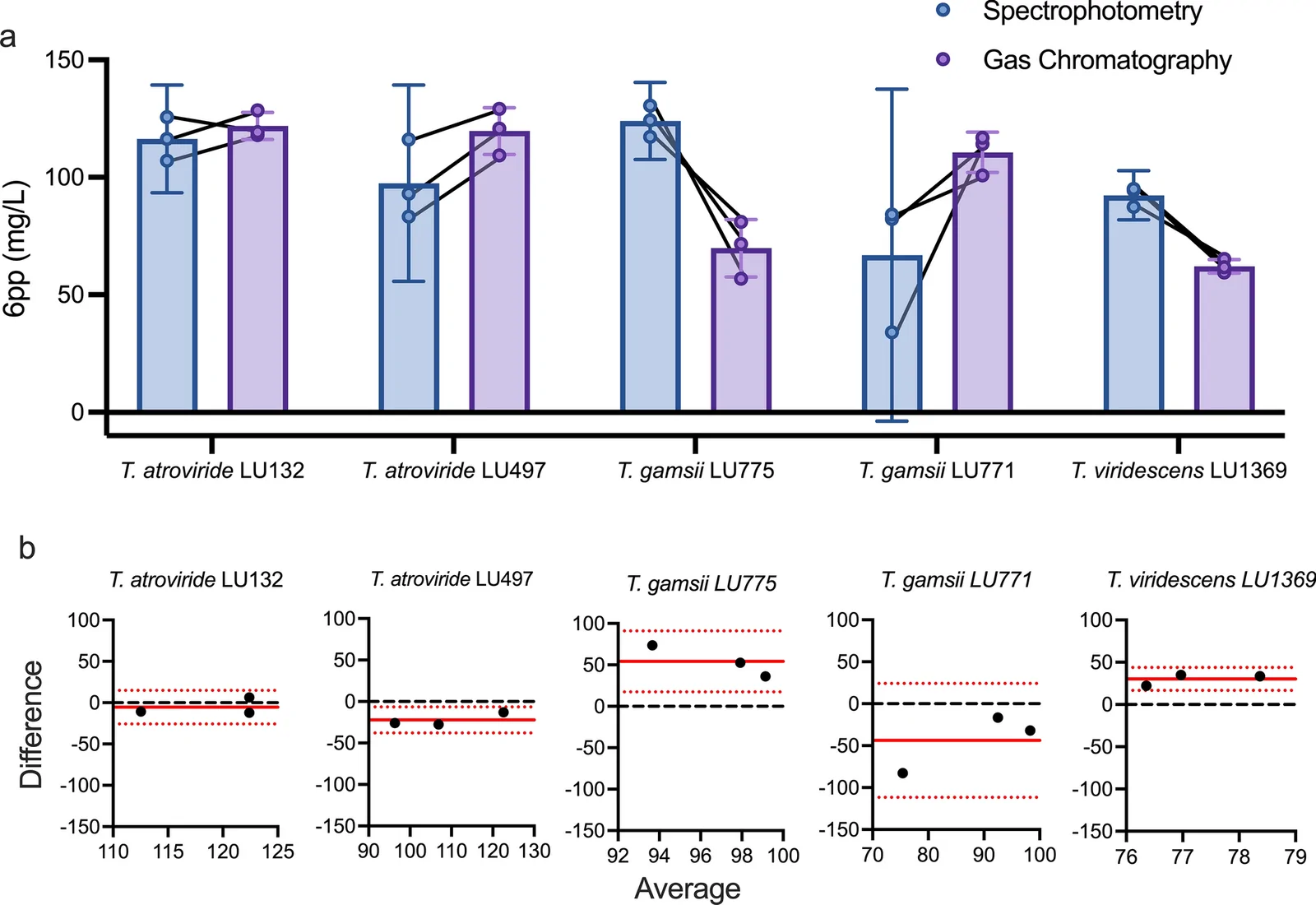
Comparison of 6-PP quantification methods in five *Trichoderma* strains cultured for 14 days. **a** A bar chart of 6-PP measurements of five *Trichoderma* strains using spectrophotometry (320 nm) and GC. Individual samples are plotted, and matched samples are connected with a line between measurement methods. Error bars represent 95% CI. **b** Bland–Altman plots showing the measurement disagreement of spectrophotometry against GC. The y-axis represents the difference between measurement types, and the x-axis represents the average between the measurement types. The solid red line represents the mean disagreement, and the dotted red lines show 95% limits of agreement

Quantification of 6‑PP in 14-day culture supernatants without Amberlite differed between spectrophotometric measurements at 320 nm and by GC analysis. Spectrophotometric estimates ranged from 33.99 to 130.5 mg/L, whereas GC values ranged from 56.83 to 129.1 mg/L, with individual samples differing by up to 82.82 mg/L (e.g. *T. gamsii* LU771). The Bland–Altman analysis showed strain‑dependent, bidirectional bias: spectrophotometry underestimated 6‑PP for some strains and overestimated it for others (Fig. 3), consistent with interference from additional 320 nm‑absorbing components detected by TLC scanning (Supplementary Fig. 2). Because GC provides more accurate quantification, GC measurements were used for all subsequent analyses.

#### Effect of Amberlite XAD-4 in situ 6-PP extraction on five *Trichoderma* strains

**Fig. 4:**
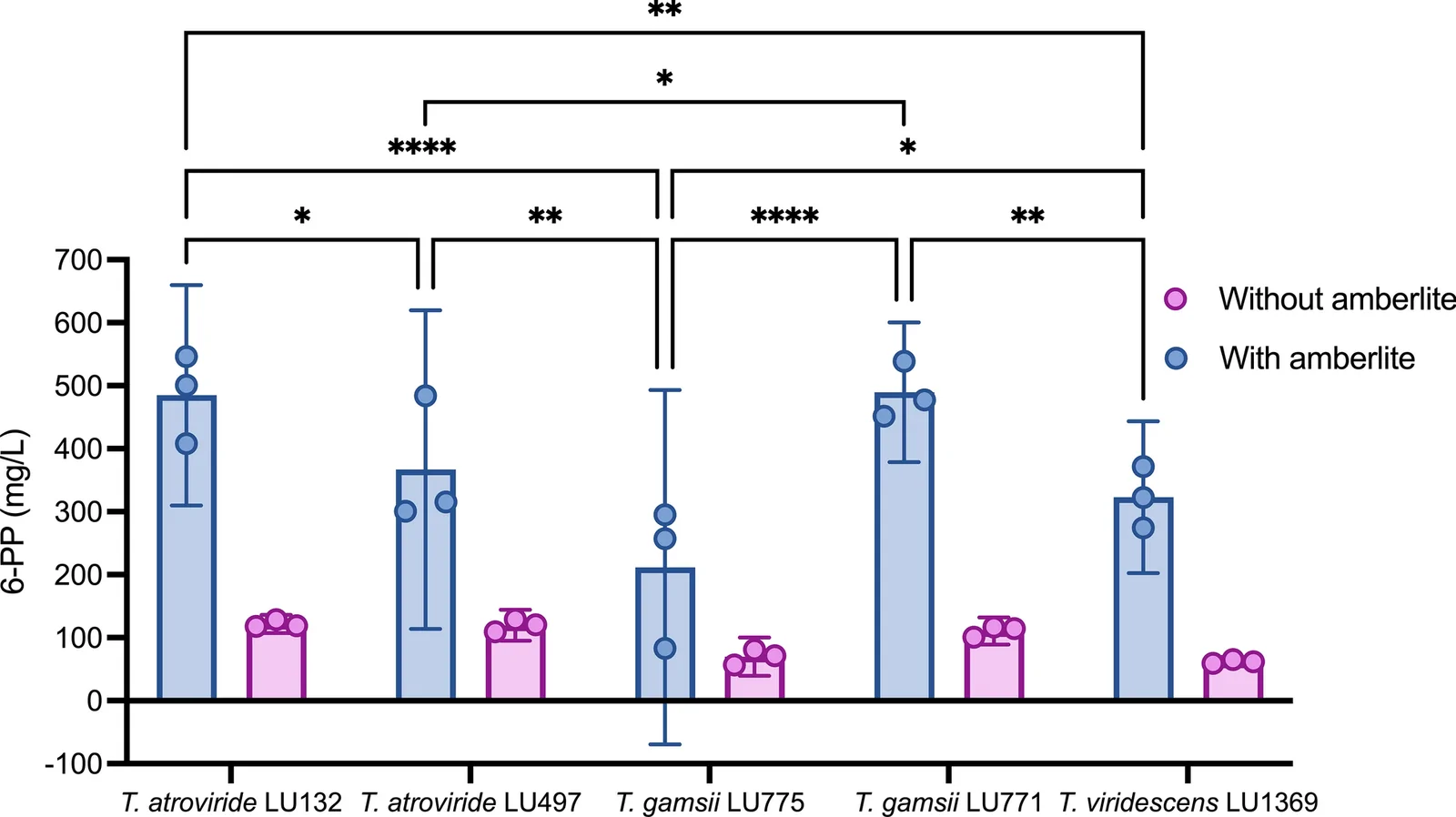
Comparison of 6-PP production in *Trichoderma* strains *T. atroviride* LU132, *T. atroviride* LU497, *T. gamsii* LU775, *T. gamsii* LU771, and *T. viridescens* LU1369 cultured with or without Amberlite XAD-4 as measured by gas chromatography. Bars represent the mean of three biological replicates. Individual replicate values are plotted, and error bars represent 95% CI. Pairwise comparisons are shown between strains cultured with Amberlite, and significance is indicated (*p* = 0.05 = *, *p* = 0.005 = **, *p* = 0.0005 = ***, *p* < 0.0001 = ****)

In cultures grown with Amberlite, GC analysis of Amberlite extracts showed 6‑PP concentrations of 211.82–489.4 mg/L, while residual 6‑PP in the corresponding supernatants was only 0.59–3.99 mg/L, indicating that > 99% of the secreted 6‑PP was sequestered by the Amberlite for all strains. In contrast, control cultures without Amberlite contained 62.13–121.86 mg/L 6‑PP in the supernatant. For each strain, the presence of Amberlite increased total 6‑PP production (Amberlite extract + supernatant) by approximately 3- to 5.2‑fold relative to cultures without Amberlite (Fig. 4).

There was a significant strain and sample type (Amberlite extract, Amberlite supernatant, control supernatant) interaction (F (8, 30) = 5.384, *p* < 0.001, η^2^ = 0.069), indicating that the level of 6-PP in the three different sample types varied by strain. The sample type accounted for the largest proportion of variance in 6‑PP levels (F(2, 30) = 258.0, *p* < 0.001, η^2^ = 0.825), followed by the strain main effect (F (4, 30) = 9.052, *p* < 0.001, η^2^ = 0.058). Within each strain, Amberlite cultures produced significantly more total 6‑PP than controls, and in all cases 6‑PP concentrations were substantially higher in Amberlite extracts than in the corresponding supernatants, confirming highly efficient in situ capture. Between strains, *T. gamsii* LU771 and *T. atroviride* LU132 achieved the highest 6‑PP titres in the Amberlite extracts with mean values of 489.4 mg/L and 484.9 mg/L, respectively, while *T. gamsii* LU775 showed the lowest production among the tested isolates with a mean value of 211.8 mg/mL.

GC–MS analysis confirmed that 6-PP was the predominant pyrone produced by all strains tested, while only minor amounts of structurally related analogues were detected (Supplementary Table 4, Supplementary Fig. 3). These findings support the accuracy of the GC-based quantification. Chromatograms of Amberlite extracts also indicated accumulation of additional unidentified metabolites alongside 6‑PP (Supplementary material 2).

Overall, these results demonstrate that Amberlite XAD-4 effectively sequestered 6‑PP from the culture supernatant across all tested *Trichoderma* strains and increased total 6‑PP production. There were still clear strain‑specific differences in production capacity despite an absence of 6-PP product inhibition.

## Discussion

This study evaluated the effect of media type on 6-PP production in one strain of *T. atroviride*, screened ten *Trichoderma* strains for 6-PP production, and assessed the capacity of Amberlite XAD-4 to perform in situ removal of 6-PP and overcome product inhibition in a subset of five strains.

### Effect of media type on 6-PP production in *T. atroviride* IMI206040

PDB supported the highest 6-PP production in *T. atroviride* IMI206040, followed by MMNO₃, while production was repressed below the detection level in MMNH₄. Greater 6-PP production in PDB relative to minimal media is consistent with previous reports that nutrient-rich media promote higher 6-PP titres [13, 16]. However, the differential production observed between the two minimal media, which contained different but equimolar nitrogen sources, is more informative.

Because the carbon-to-nitrogen ratios were identical, the suppression of 6-PP in MMNH₄ relative to MMNO₃ is possibly attributable to nitrogen catabolite repression (NCR). NCR is a conserved regulatory mechanism in fungi whereby preferred nitrogen sources such as NH₄⁺ repress the transcription of genes involved in the utilisation of less-preferred nitrogen sources [40]. The global nitrogen-responsive transcription factor AreA, which is active under nitrogen-limiting or non-preferred nitrogen conditions, is known to regulate secondary metabolite production broadly across fungi (orthologous to Are1 in *Trichoderma*) [40, 41]. The production pattern observed here is consistent with 6-PP biosynthesis being nitrogen-repressed, as production was higher under the less-preferred MMNO₃ condition. This contrasts with reports in *T. viride* where NH₄⁺ enhanced 6-PP production [13, 26], which may reflect variation in NCR sensitivity compared to *T. atroviride* IMI206040, or differences in carbon-to-nitrogen ratios between studies. Based on these results, PDB was selected for subsequent experiments to maximise 6-PP production.

### Comparison of 6-PP production in ten *Trichoderma* strains

All ten *Trichoderma* strains produced 6-PP under standardised liquid fermentation conditions in PDB. Substantial strain-to-strain variation was observed, with up to a 14.6-fold difference between the highest and lowest producers as measured by spectrophotometry. *T. viridescens* LU1369 and *T. gamsii* LU771 emerged as the highest producers, while *T. atroviride* strains were mid to low producers. This pattern is consistent with previous reports that *T. gamsii* and *T. viridescens* are often among the highest 6-PP producers in comparative screening assays [13].

Mycelial biomass was relatively consistent among strains, except for *T. viridescens* LU1369 showing the largest biomass accumulation. The ten-strain screening was based on a single timepoint assessment (7 days), so the results may reflect cumulative yield capacity rather than peak 6-PP titres, which likely vary in timing across strains and species [16, 28]. Strains with a peak production curve occurring prior to day 7, for example *T. atroviride* IMI206040, which produced higher 6-PP levels after 5 days in the media experiment, may have already begun degrading 6-PP by the time of sampling. This may partly explain why *T. gamsii* and *T. viridescens* outperformed *T. atroviride* at this time point, if the former strains have comparatively longer or delayed production curves, or reduced sensitivity to 6-PP self-inhibition. Taken together, the endpoint screening approach used here may underestimate the 6-PP production potential of some strains, so for additional screening in situ 6-PP removal was implemented.

### Spectrophotometric 6-PP quantification measurement error

Spectrophotometric quantification of 6-PP from ethyl acetate supernatant extracts at 320 nm and calibrated against a pure 6-PP standard, has previously been reported as a rapid and efficient measurement method [22], and it was used as the quantification approach in the 10 strain screening experiment of this study. However, after GC was implemented, differences between spectrophotometric estimates and GC measurements were observed, with both the magnitude and direction of the measurement disagreement varying among samples. Scanning TLC plates confirmed the presence of additional 320 nm absorbing compounds whose abundance varied among strains. This suggests that the spectrophotometric estimates may be influenced by co-extracted metabolites with overlapping absorbance spectra, particularly when comparing strains with distinct secondary metabolite profiles. While spectrophotometric measurements provide a rapid screening approach, GC offered greater analytical specificity in our system. Consequently, GC was used as the primary quantification method for the in situ extraction experiment, and may be preferable for future 6-PP fermentation studies.

### Effect of in situ 6-PP extraction with Amberlite XAD-4

The addition of Amberlite XAD-4 resulted in a 3- to 5.2-fold relative increase in total 6-PP production across all five strains tested, with > 99% of secreted 6-PP sequestered by the resin in all cases. This confirms that product inhibition was effectively eliminated and that all strains were sensitive to 6-PP-mediated self-inhibition under standard conditions. These findings are consistent with previous reports of 6-PP in situ product removal methods [18, 19, 29], including adsorbent resins. Prapulla and Engel [16] found a 2.75-fold increase in 6-PP production to ~ 248 mg/L in *T. viride* cultured with Amberlite XAD-2. Here, we found higher 6-PP titres with individual samples exceeding 500 mg/L, albeit with a significantly longer culture duration. Additionally, Amberlite XAD-4 has approximately twice the surface area of Amberlite XAD-2 and smaller pores making it more suitable for small organic molecules such as 6-PP.

Despite the alleviation of product inhibition, clear strain-specific differences in 6-PP production capacity persisted, indicating that inherent biosynthetic capacity, not just inhibition tolerance, determines final titres. The ranking of strains by 6-PP production differed between the initial ten-strain screening and the Amberlite experiment. For the *T. atroviride* strains, LU132 performed poorly in the initial screening, but achieved the second-highest 6-PP titre with Amberlite, approaching that of LU497. LU132 and LU497 appeared less prone to 6-PP measurement disagreement between spectrophotometric and GC measurements, so this reversal suggests that LU132 may have a higher intrinsic production capacity but lower self-inhibition tolerance than LU497, and that endpoint supernatant measurements without in situ removal do not reliably reflect production capacity potential.

Across strains, *T. gamsii* LU771 and *T. atroviride* LU132 achieved the highest 6-PP titres in the presence of Amberlite, with mean values of 489.4 mg/L and 484.9 mg/L, respectively, demonstrating the potential for further process optimisation of these isolates. These concentrations substantially exceed those typically reported from conventional liquid fermentation and represent progress toward economically viable bioproduction [14, 16, 18–20, 28]. However, 6-PP production in the Amberlite cultures may have been further influenced by gradual nutrient depletion over the 14-day fermentation period. Future studies should track these parameters, as 6-PP production is likely affected by nutrient availability. As such, additional nutrient supplementation using the current method is likely to further increase 6-PP titres.

## Conclusion

This study screened New Zealand *Trichoderma* isolates for 6‑PP production and evaluated culture conditions to enhance titres by alleviating self‑inhibition. Media composition, particularly nitrogen source, significantly influenced 6-PP production in *T. atroviride* IMI206040, with results consistent with nitrogen catabolite repression. Furthermore, in situ 6-PP removal with Amberlite XAD-4 increased total 6-PP titres 3- to fivefold across all five strains tested, with near-complete sequestration of secreted 6-PP confirming that product inhibition is a universal constraint in the isolates assessed. The highest-producing strains, *T. gamsii* LU771 and *T. atroviride* LU132, achieved titres of almost 500 mg/L under Amberlite conditions, approaching economically viable bioproduction. These findings support the use of Amberlite XAD-4 as an in situ product removal strategy for both 6-PP bioprocess development and screening *Trichoderma* under conditions that eliminate self-inhibition to uncover strains with high intrinsic production capacity.

## Supplementary Information

Additional file 1.

Additional file 2.

## Data Availability

The data is included as supplementary data.
