# Supplementary material for: Increase in 6-pentyl-alpha-pyrone production by Trichoderma spp. in liquid medium using in situ removal by adsorbent resin
Source: Nat Prod Bioprospect. 2026 Oct 19;16(1):93. doi: 10.1007/s13659-026-00650-y (PMC13680593; doi:10.1007/s13659-026-00650-y)
Supplement: Supplementary file 1 [file 13659_2026_650_MOESM2_ESM.pdf]

Chromatogram No.	Strain	Replicate	Sample type	notes
1	LU132	a (with amberlite)	amberlite resin extract	20x sample dilution
2	LU132	b (with amberlite)	amberlite resin extract	20x sample dilution
3	LU132	c (with amberlite)	amberlite resin extract	20x sample dilution
4	LU497	a (with amberlite)	amberlite resin extract	20x sample dilution
5	LU497	b (with amberlite)	amberlite resin extract	20x sample dilution
6	LU497	c (with amberlite)	amberlite resin extract	20x sample dilution
7	LU771	a (with amberlite)	amberlite resin extract	20x sample dilution
8	LU771	b (with amberlite)	amberlite resin extract	20x sample dilution
9	LU771	c (with amberlite)	amberlite resin extract	20x sample dilution
10	LU775	a (with amberlite)	amberlite resin extract	20x sample dilution
11	LU775	b (with amberlite)	amberlite resin extract	20x sample dilution
12	LU775	c (with amberlite)	amberlite resin extract	20x sample dilution
13	LU1369	a (with amberlite)	amberlite resin extract	20x sample dilution
14	LU1369	b (with amberlite)	amberlite resin extract	20x sample dilution
15	LU1369	c (with amberlite)	amberlite resin extract	20x sample dilution
16	LU132	a (control without amberlite)	control supernatant extract	
17	LU132	b (control without amberlite)	control supernatant extract	
18	LU132	c (control without amberlite)	control supernatant extract	
19	LU132	a (with amberlite)	amberlite supernatant extract	
20	LU132	b (with amberlite)	amberlite supernatant extract	
21	LU132	c (with amberlite)	amberlite supernatant extract	
22	LU497	a (control without amberlite)	control supernatant extract	
23	LU497	b (control without amberlite)	control supernatant extract	

24	LU497	c (control without amberlite)	control supernatant extract	
25	LU497	a (with amberlite)	amberlite supernatant extract	
26	LU497	b (with amberlite)	amberlite supernatant extract	
27	LU497	c (with amberlite)	amberlite supernatant extract	
28	LU771	a (control without amberlite)	control supernatant extract	
29	LU771	b (control without amberlite)	control supernatant extract	
30	LU771	c (control without amberlite)	control supernatant extract	
31	LU771	a (with amberlite)	amberlite supernatant extract	
32	LU771	b (with amberlite)	amberlite supernatant extract	
33	LU771	c (with amberlite)	amberlite supernatant extract	
34	LU775	a (control without amberlite)	control supernatant extract	
35	LU775	b (control without amberlite)	control supernatant extract	
36	LU775	c (control without amberlite)	control supernatant extract	
37	LU775	a (with amberlite)	amberlite supernatant extract	
38	LU775	b (with amberlite)	amberlite supernatant extract	
39	LU775	c (with amberlite)	amberlite supernatant extract	
40	LU1369	a (control without amberlite)	control supernatant extract	
41	LU1369	b (control without amberlite)	control supernatant extract	
42	LU1369	c (control without amberlite)	control supernatant extract	
43	LU1369	a (with amberlite)	amberlite supernatant extract	
44	LU1369	b (with amberlite)	amberlite supernatant extract	
45	LU1369	c (with amberlite)	amberlite supernatant extract	

Chromatogram No1

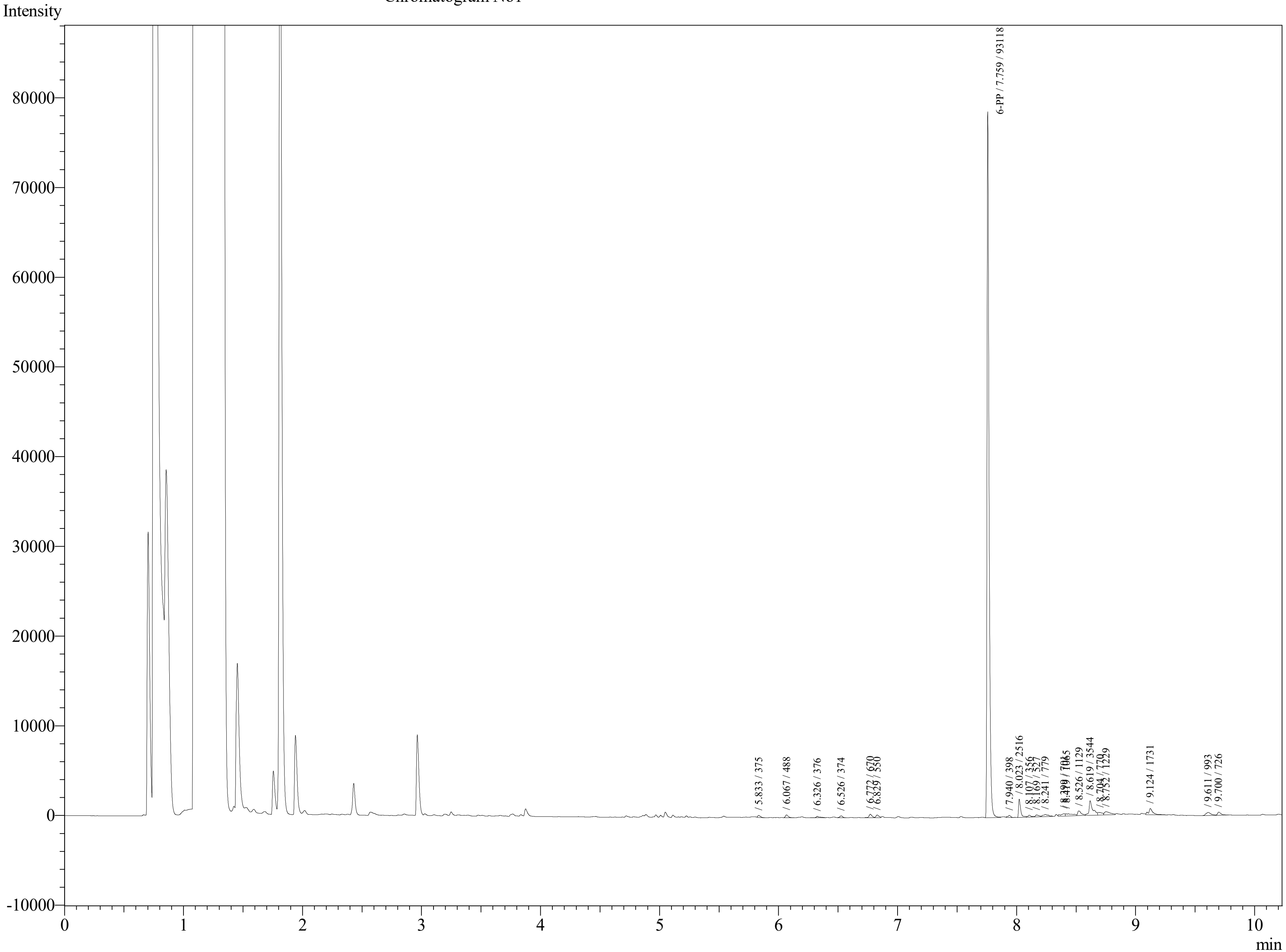

Chromatogram No. 2

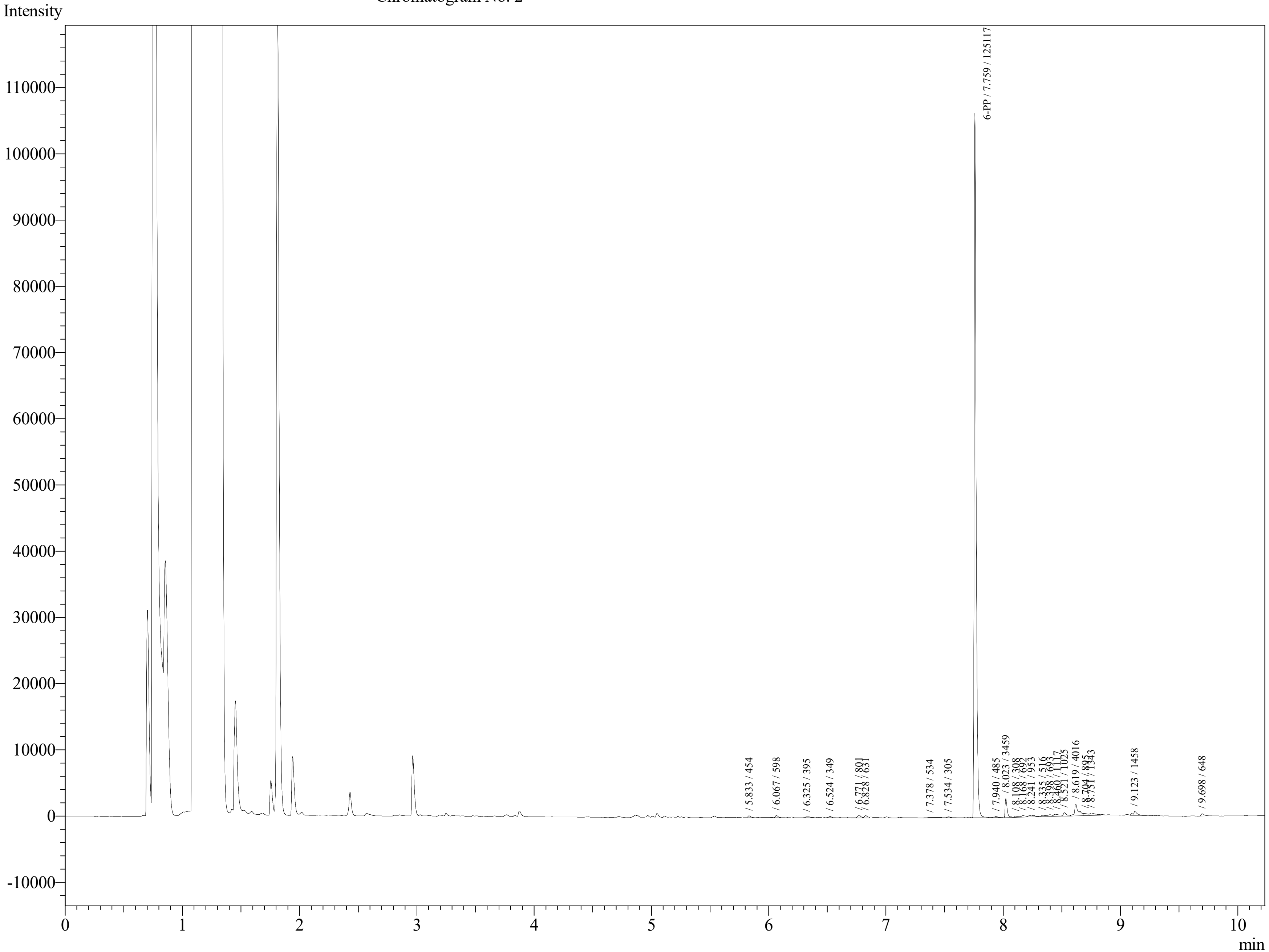

Chromatogram No. 3

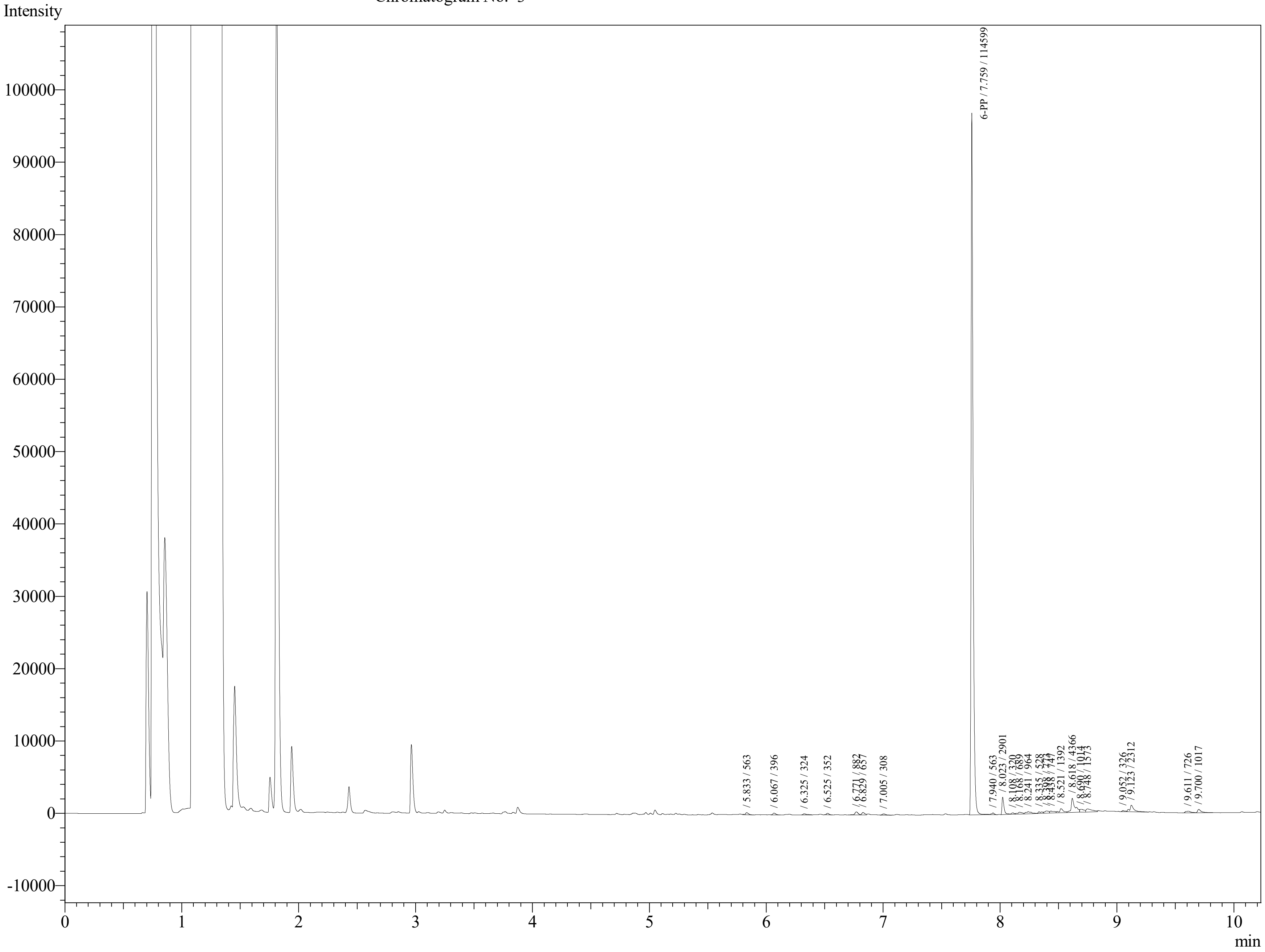

Chromatogram No. 4

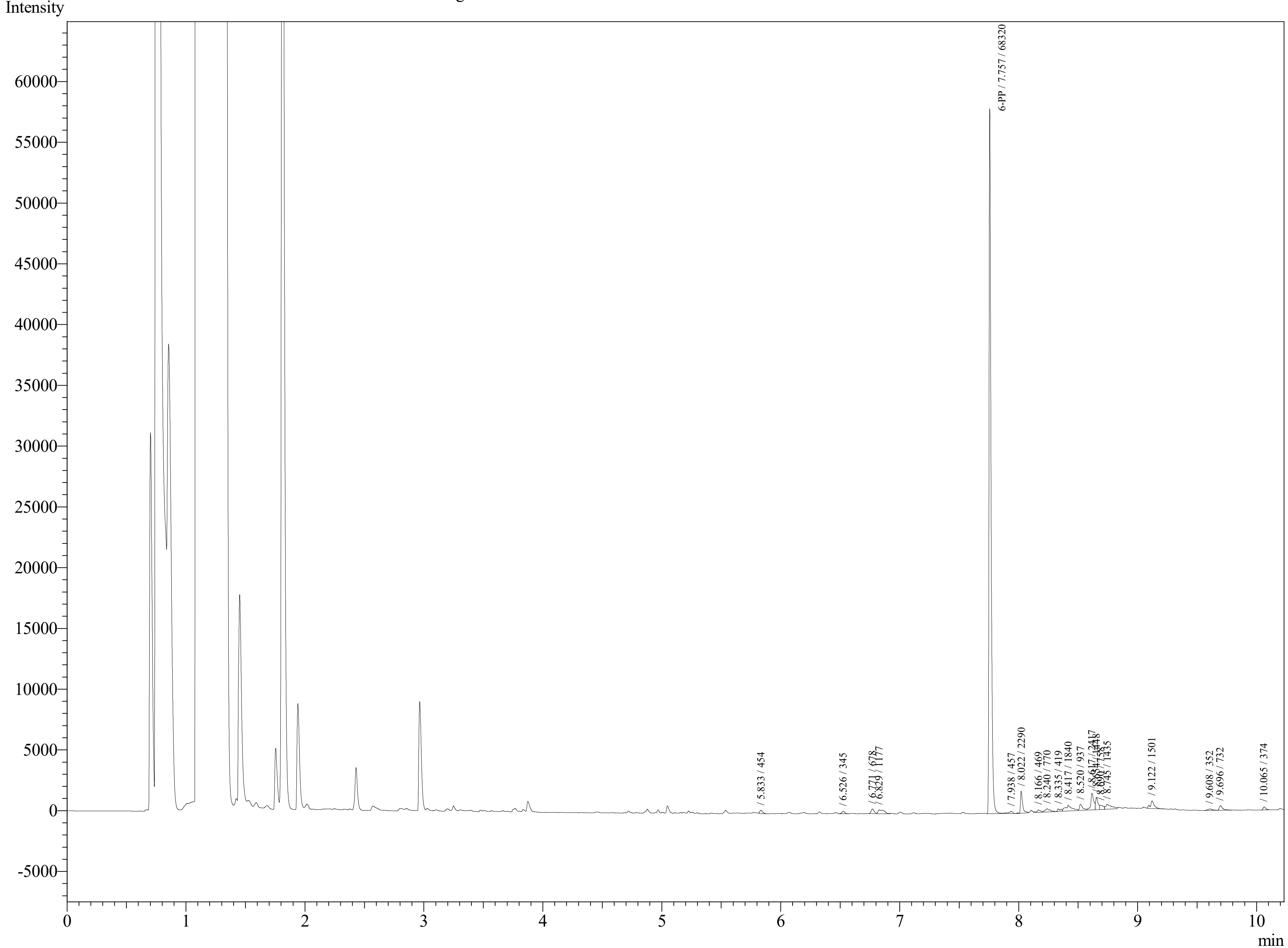

Chromatogram No. 5

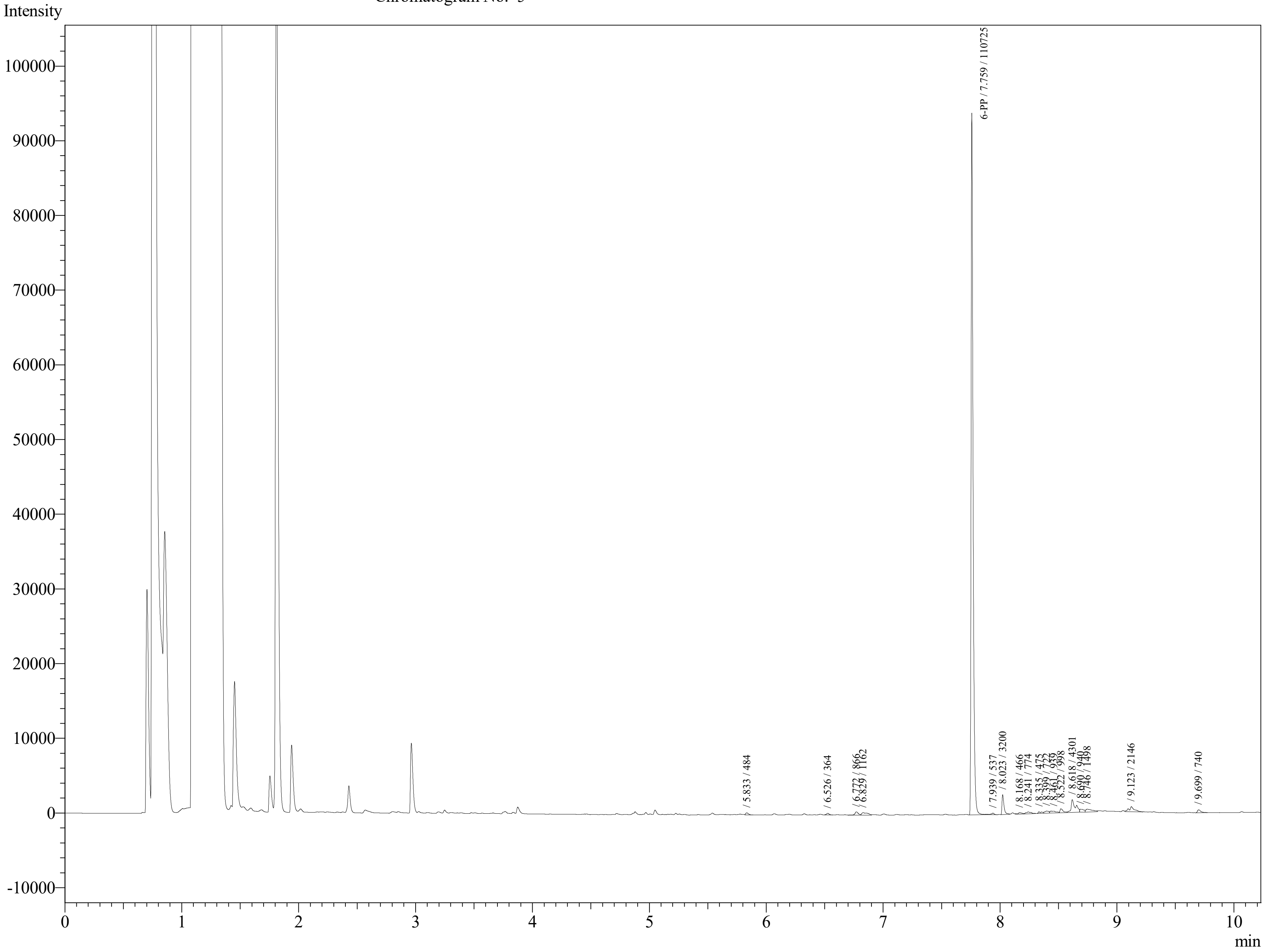

Chromatogram No. 6

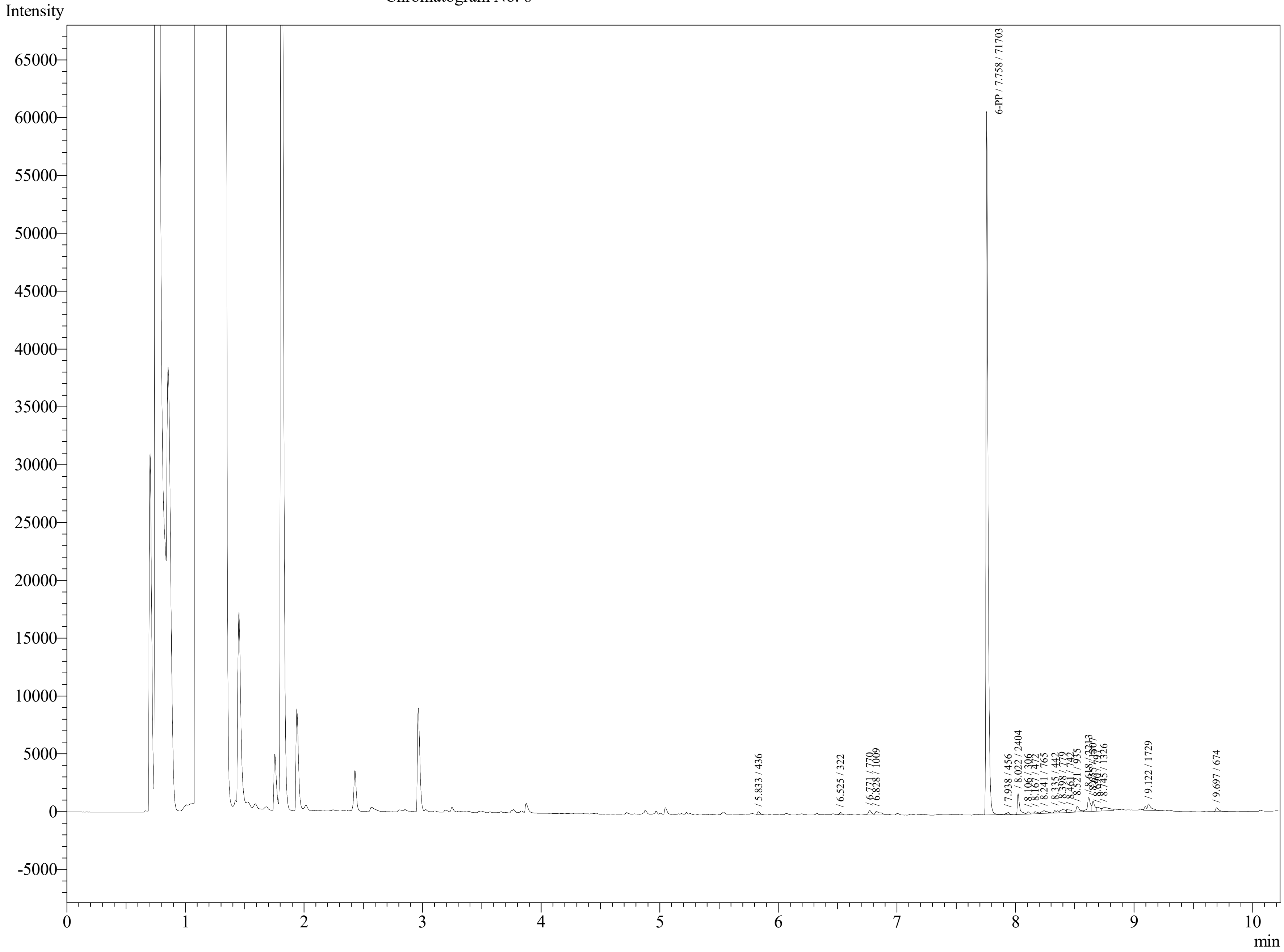

Chromatogram No. 7

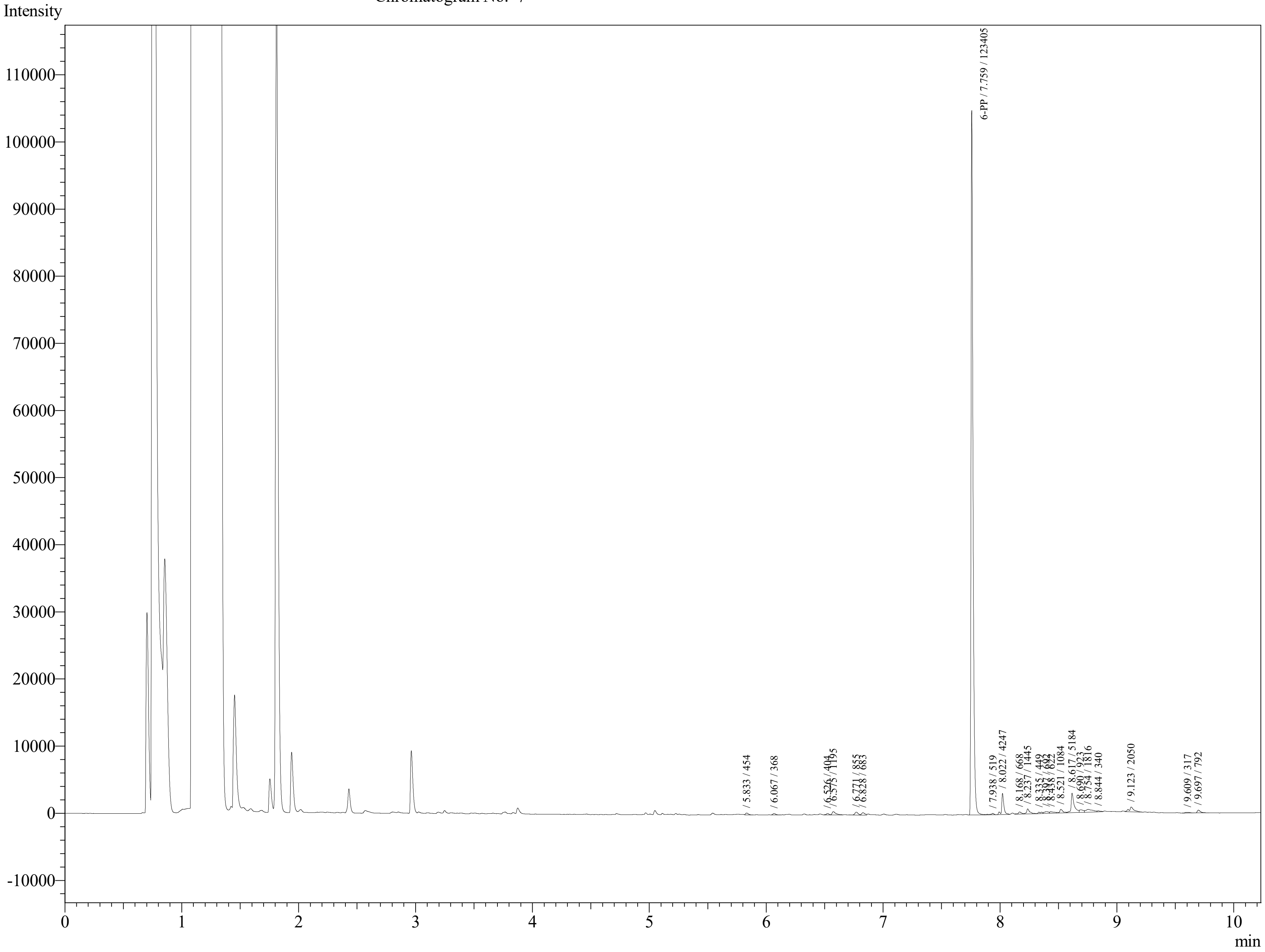

Chromatogram No. 8

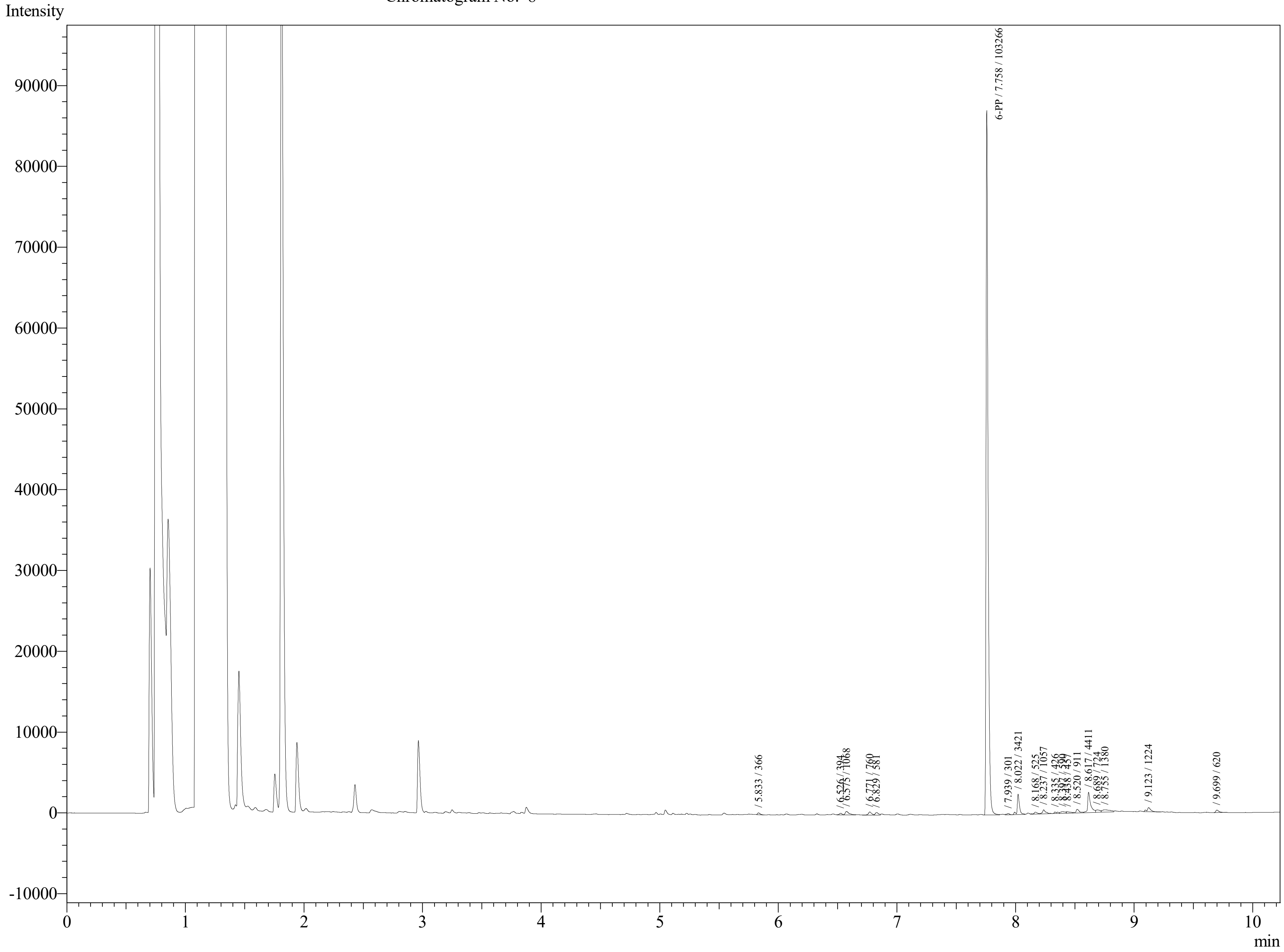

Chromatogram No. 9

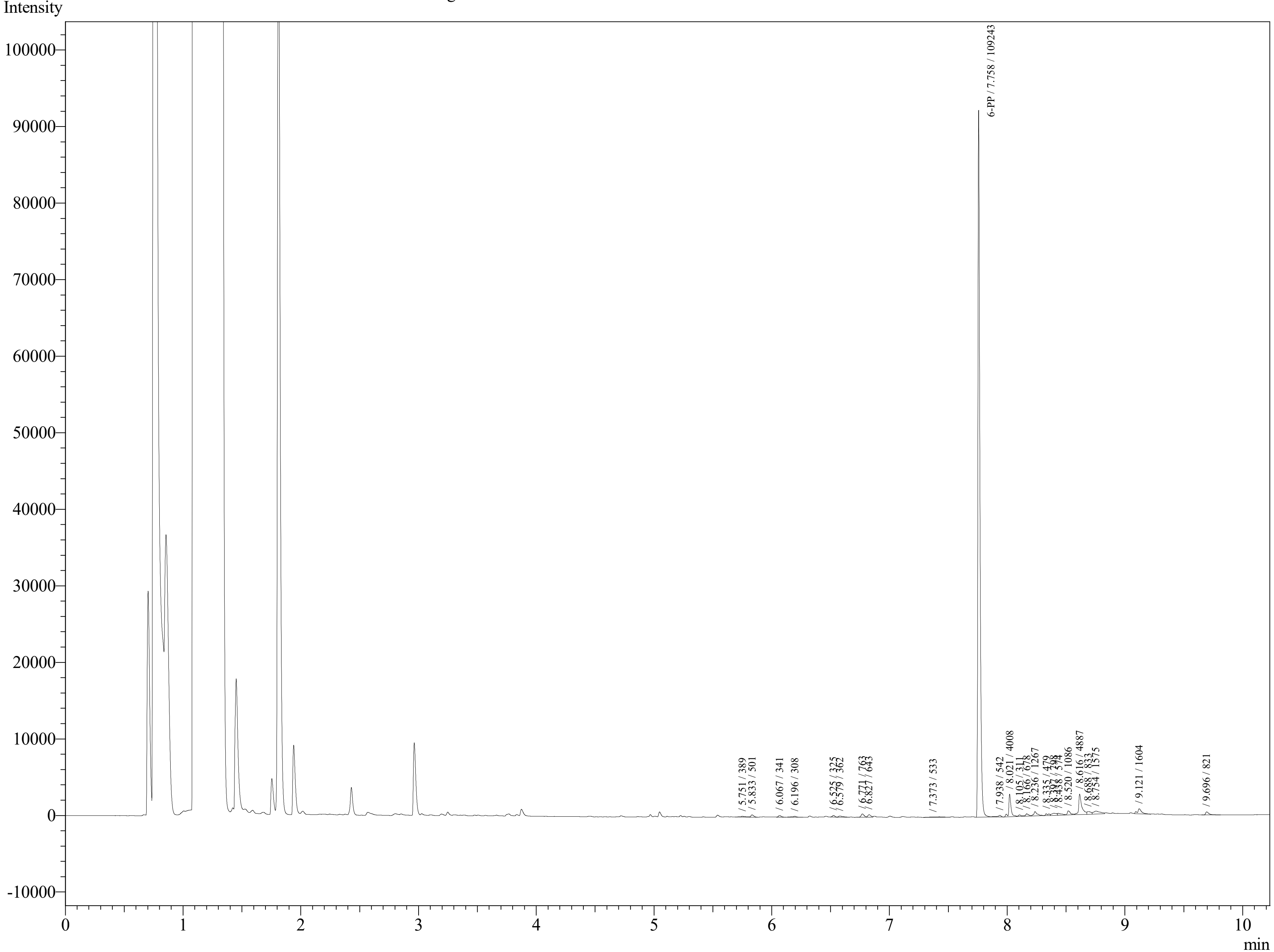

Chromatogram No 10

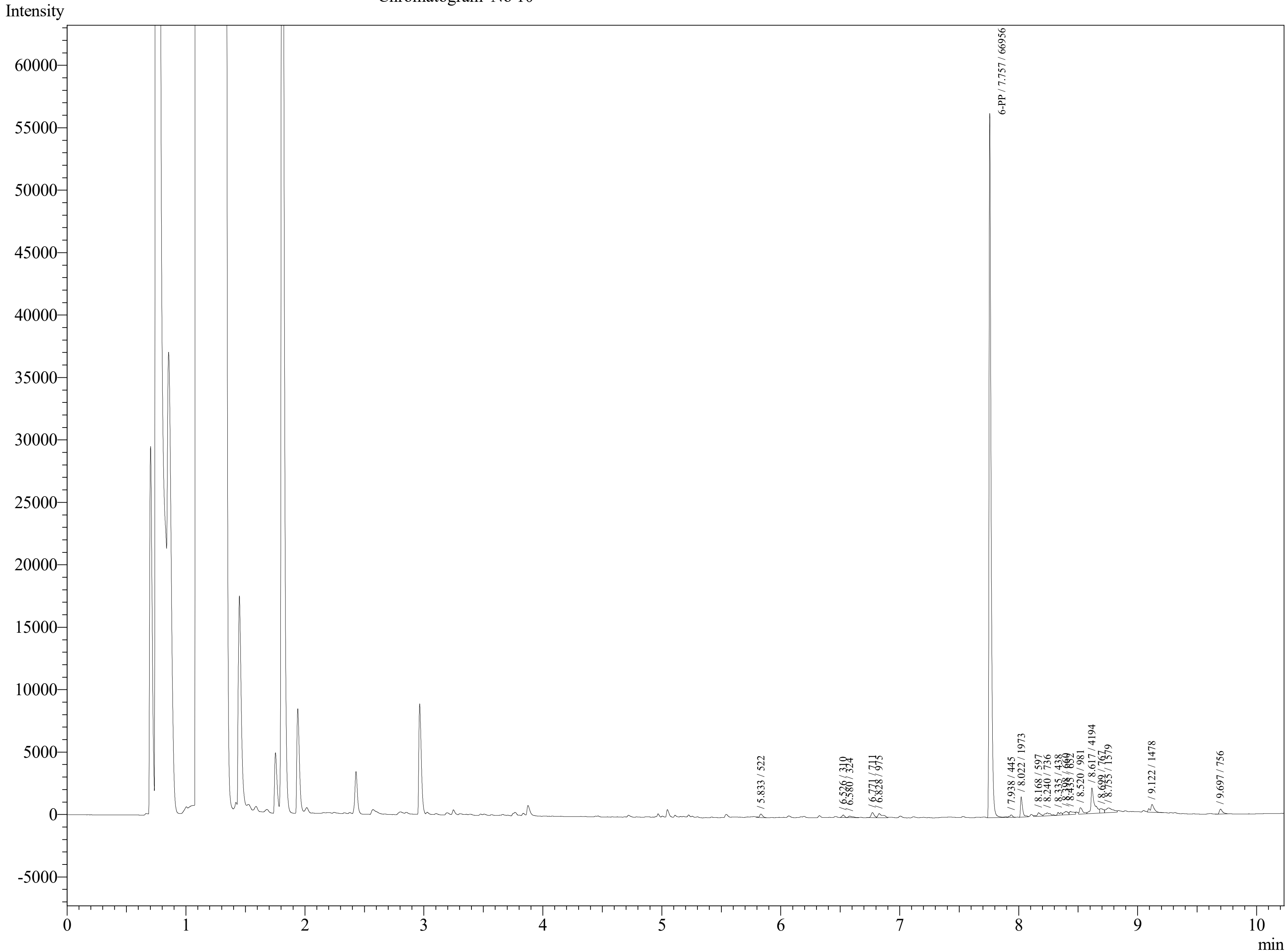

Chromatogram No. 11

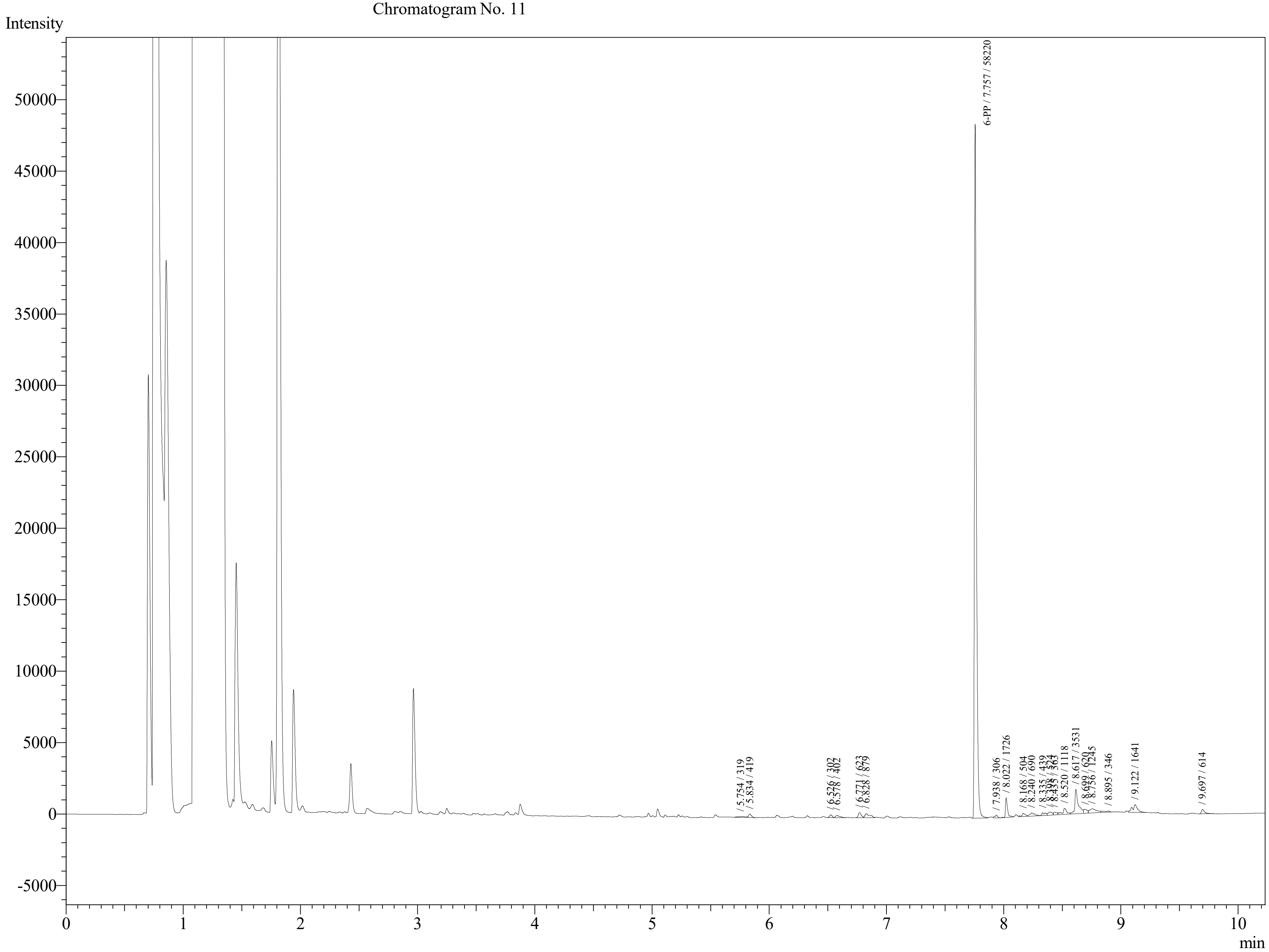

Chromatogram No. 12

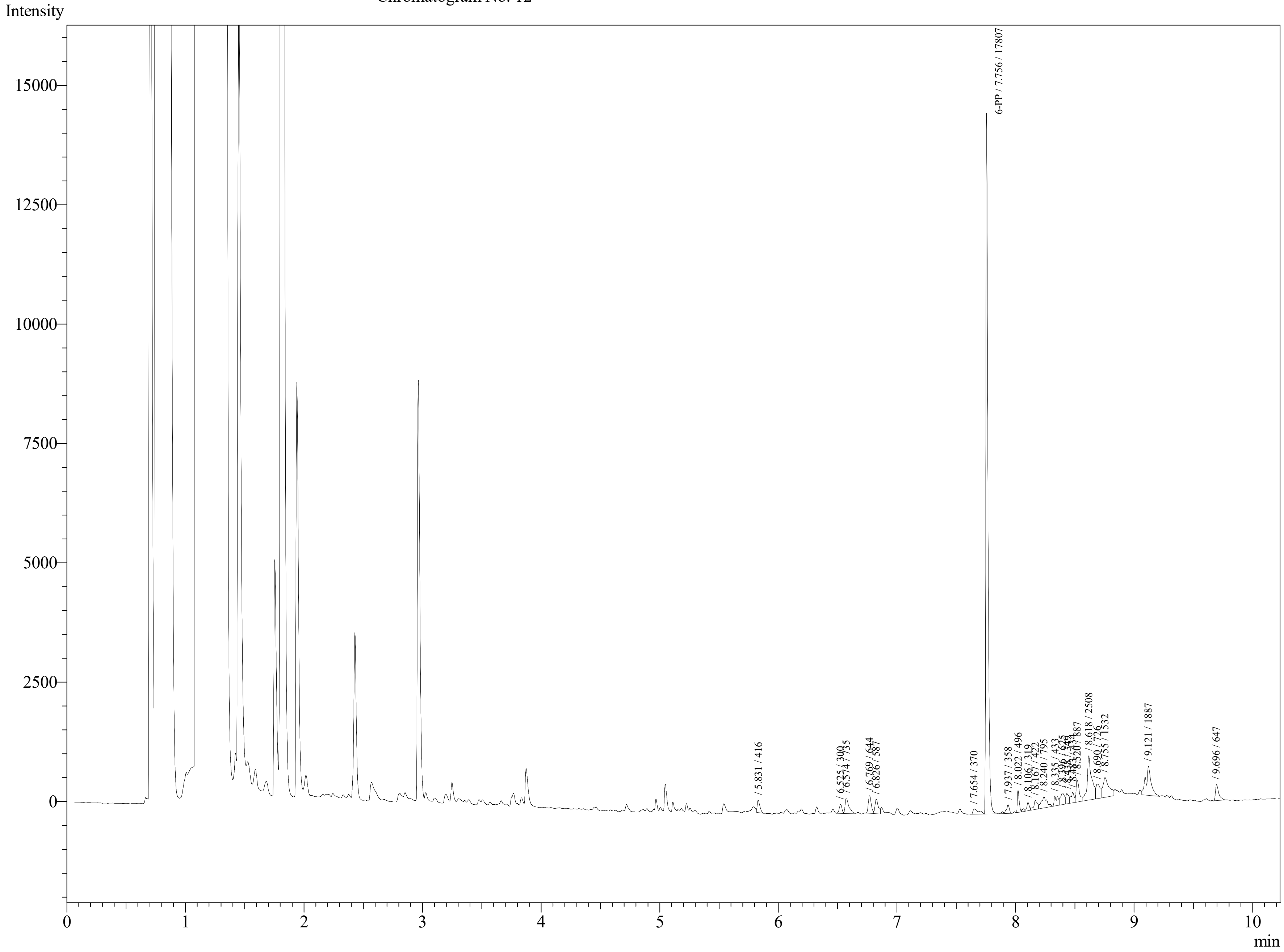

Chromatogram No. 13

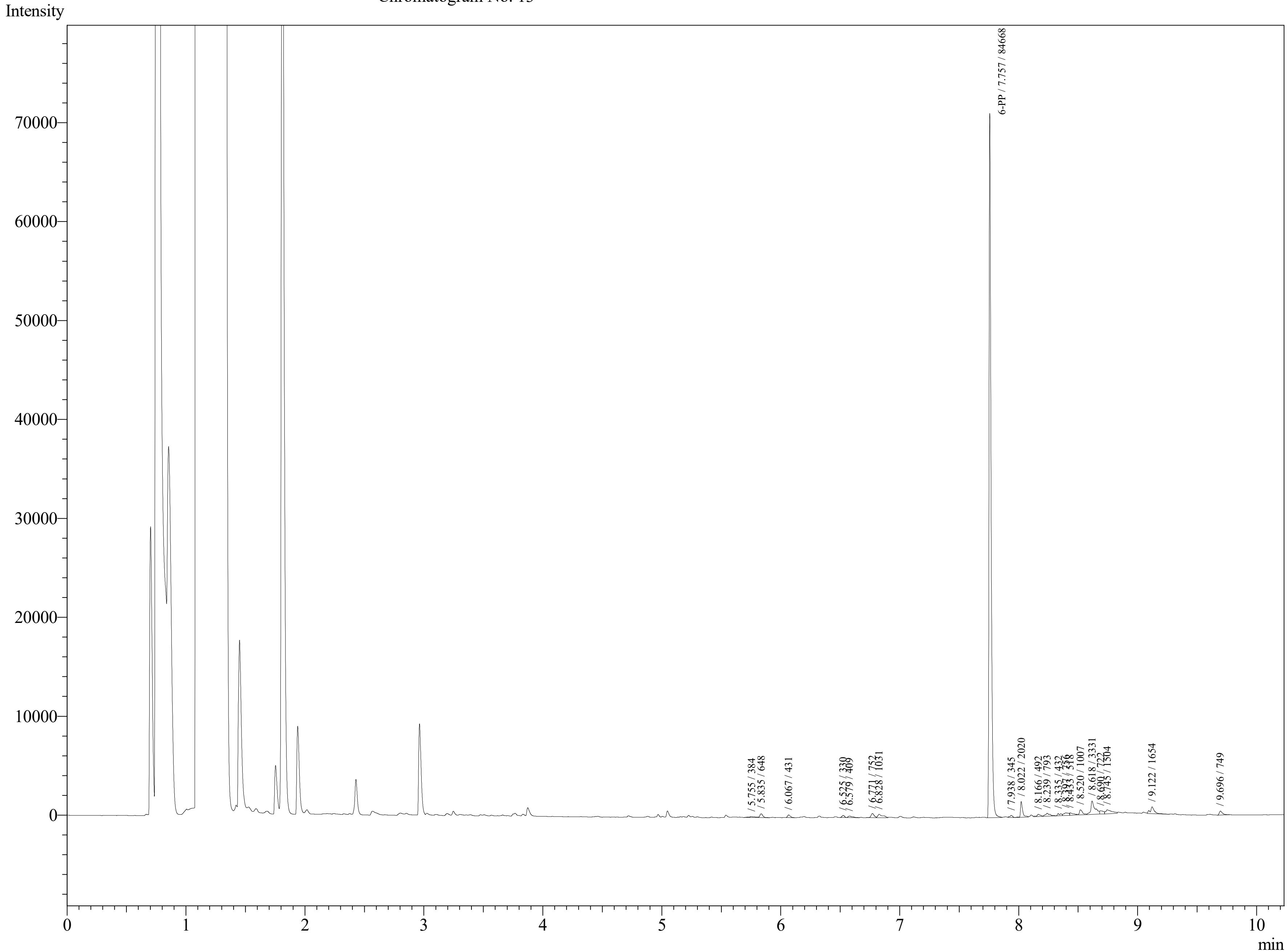

Chromatogram No. 14

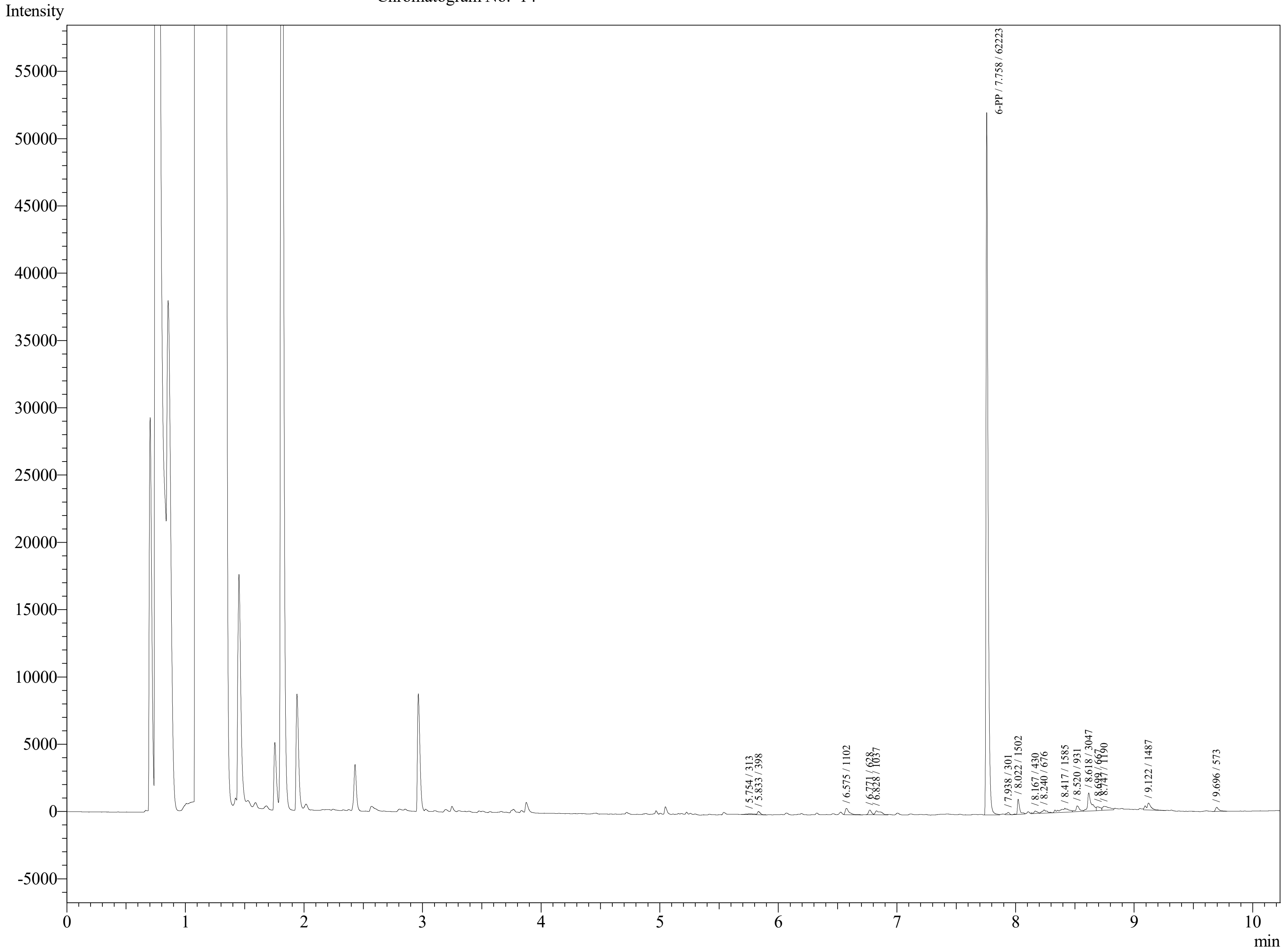

Chromatogram No. 15

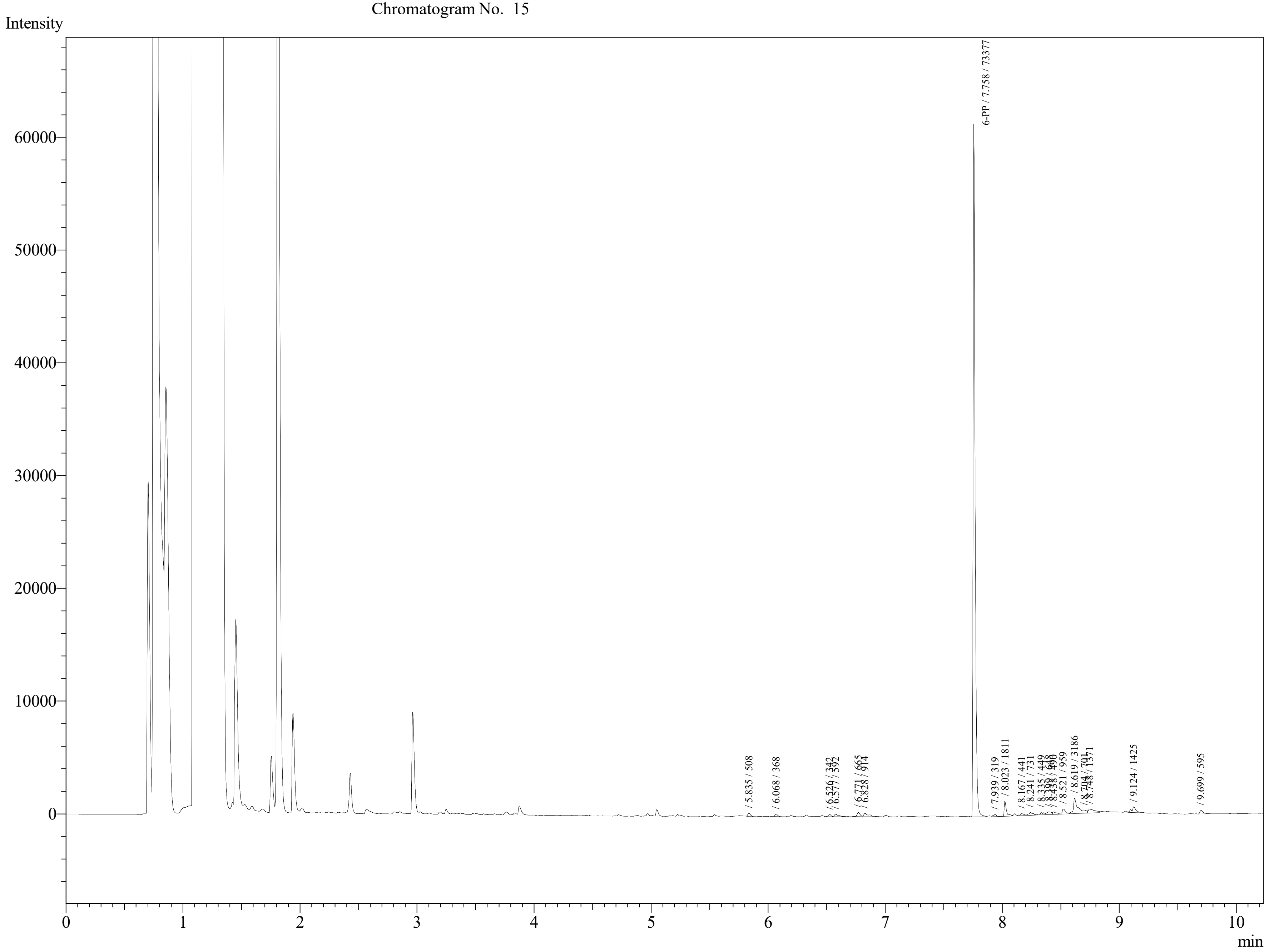

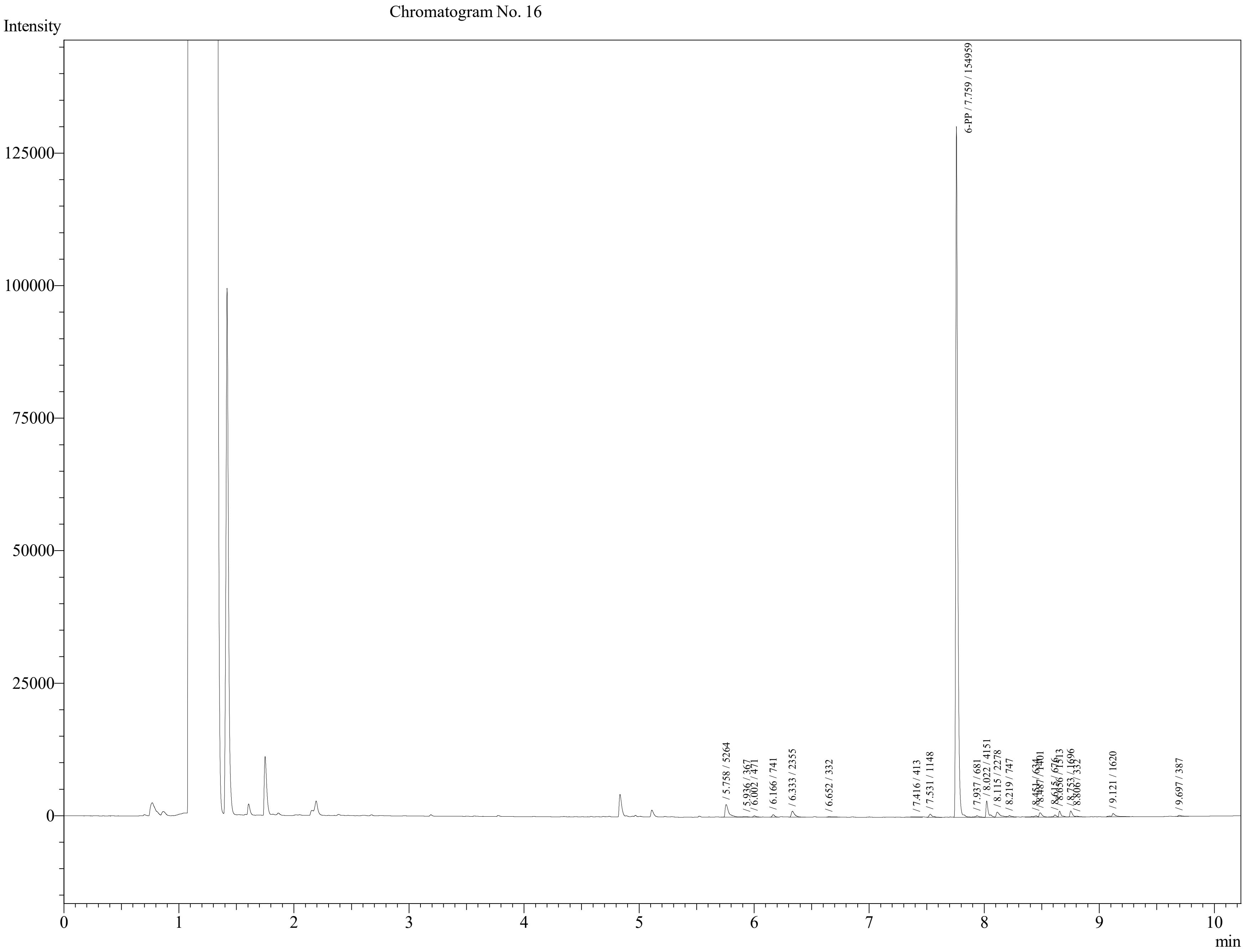

Chromatogram No. 16
Intensity
125000
100000
75000
50000
25000
0
0
1
2
3
4
5
6
7
8
9
10
min
6-PP / 7.759 / 154959
/ 5.758 / 5264
/ 5.936 / 367
/ 6.002 / 471
/ 6.166 / 741
/ 6.333 / 2355
/ 6.652 / 332
/ 7.416 / 413
/ 7.531 / 1148
/ 7.937 / 681
/ 8.022 / 4151
/ 8.115 / 2278
/ 8.219 / 747
/ 8.451 / 634
/ 8.487 / 1401
/ 8.615 / 676
/ 8.656 / 1513
/ 8.753 / 1696
/ 8.806 / 332
/ 9.121 / 1620
/ 9.697 / 387

Chromatogram No. 17

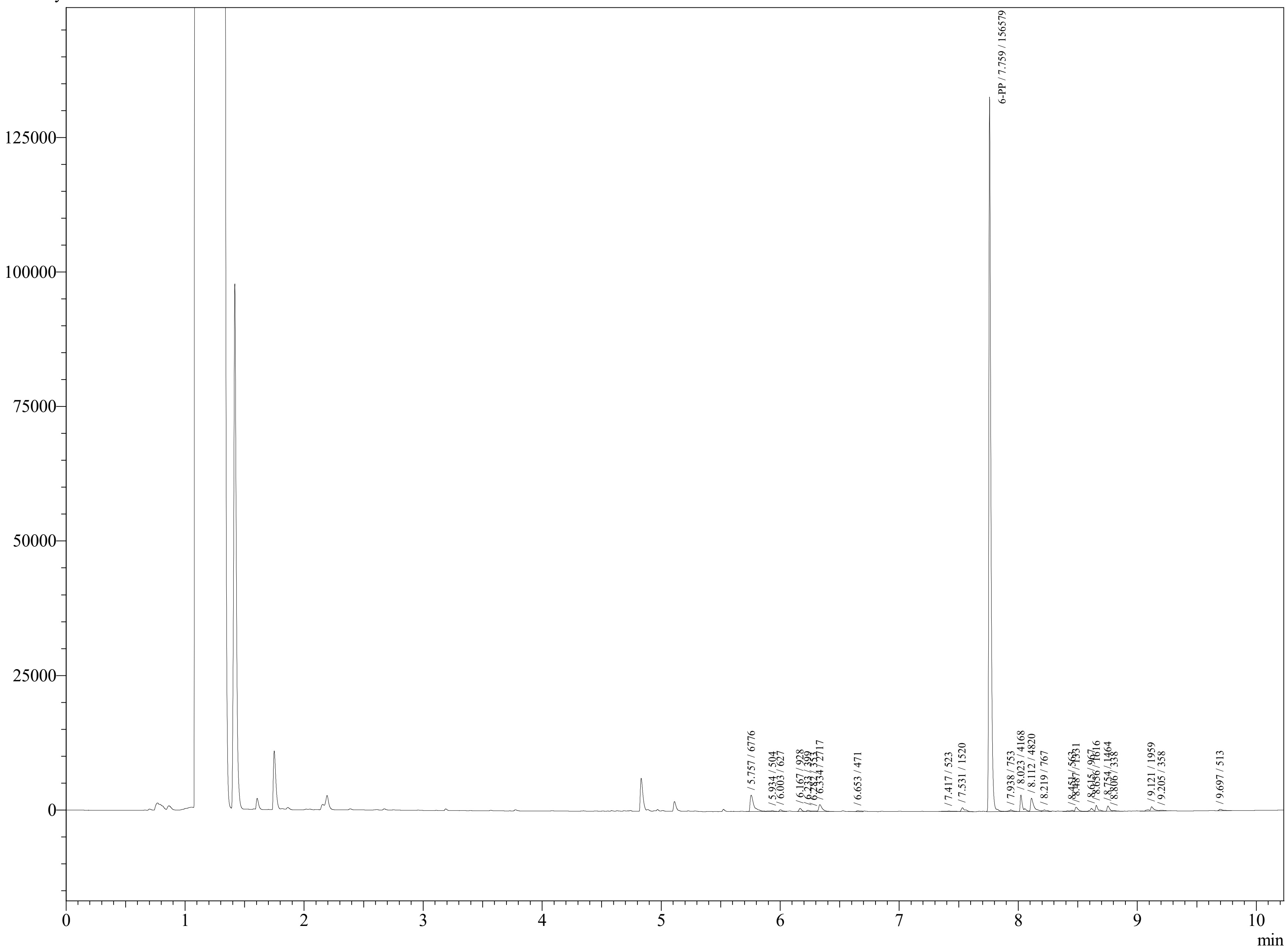

Chromatogram No. 18

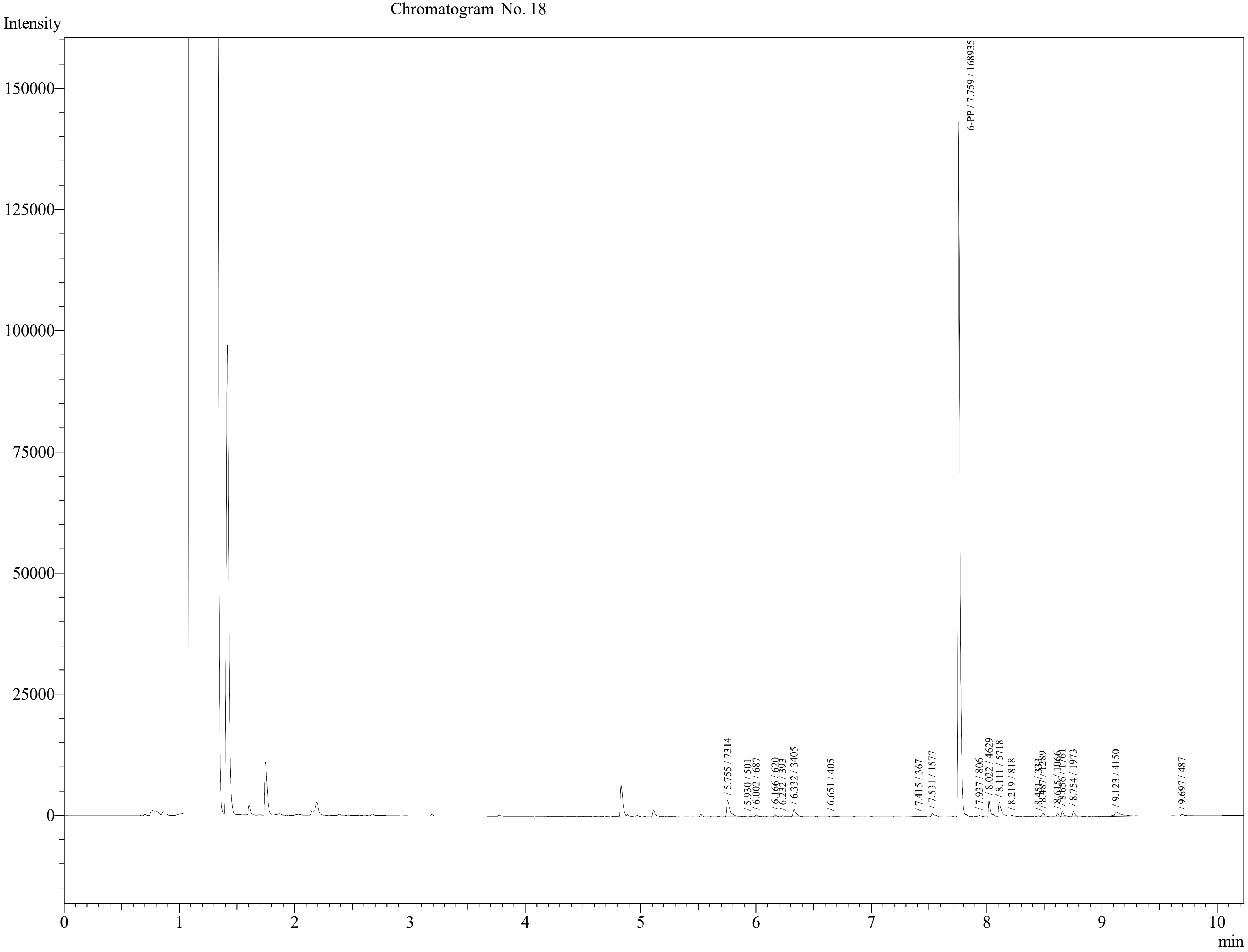

Chromatogram No. 19

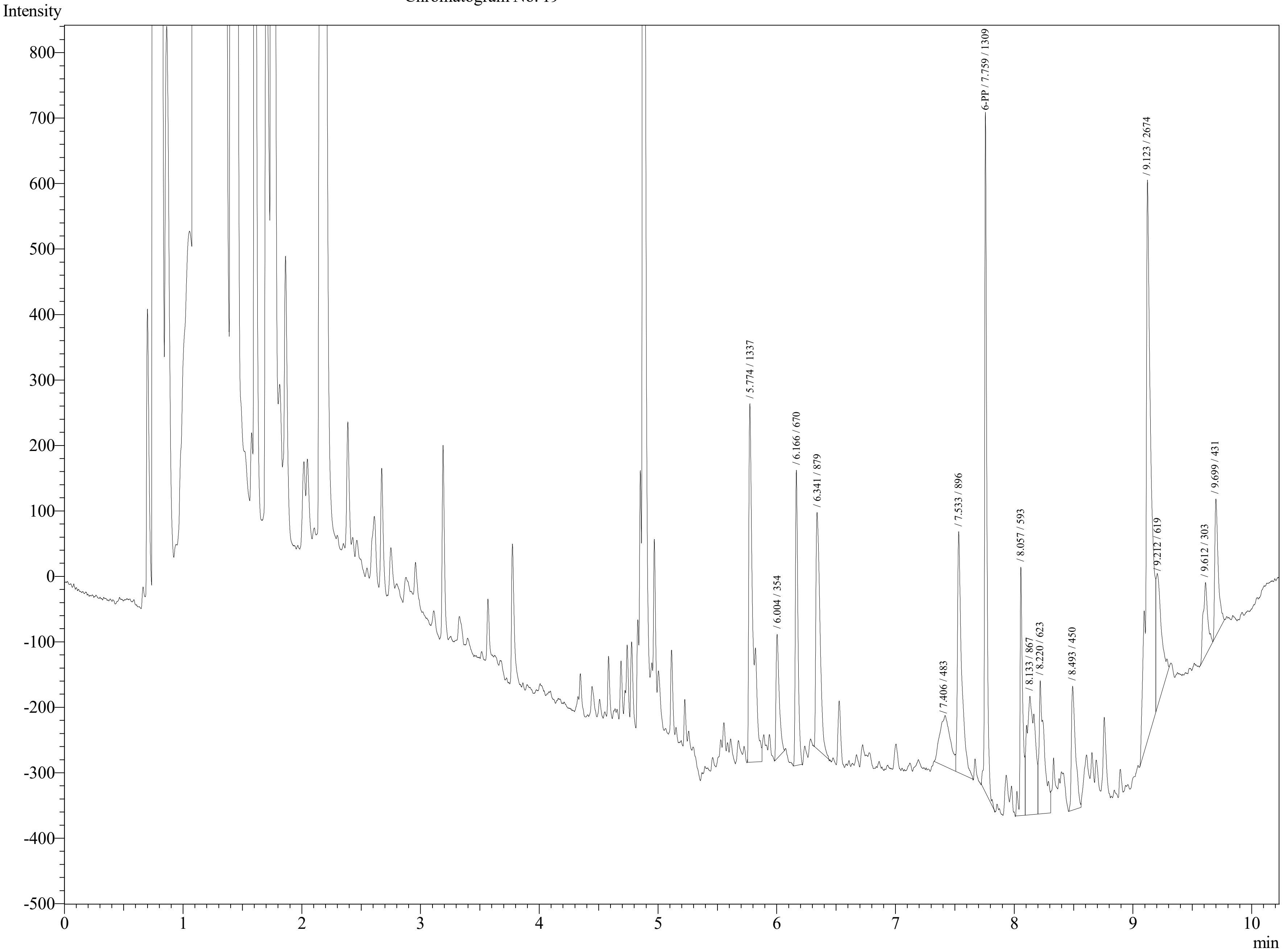

Chromatogram No. 20

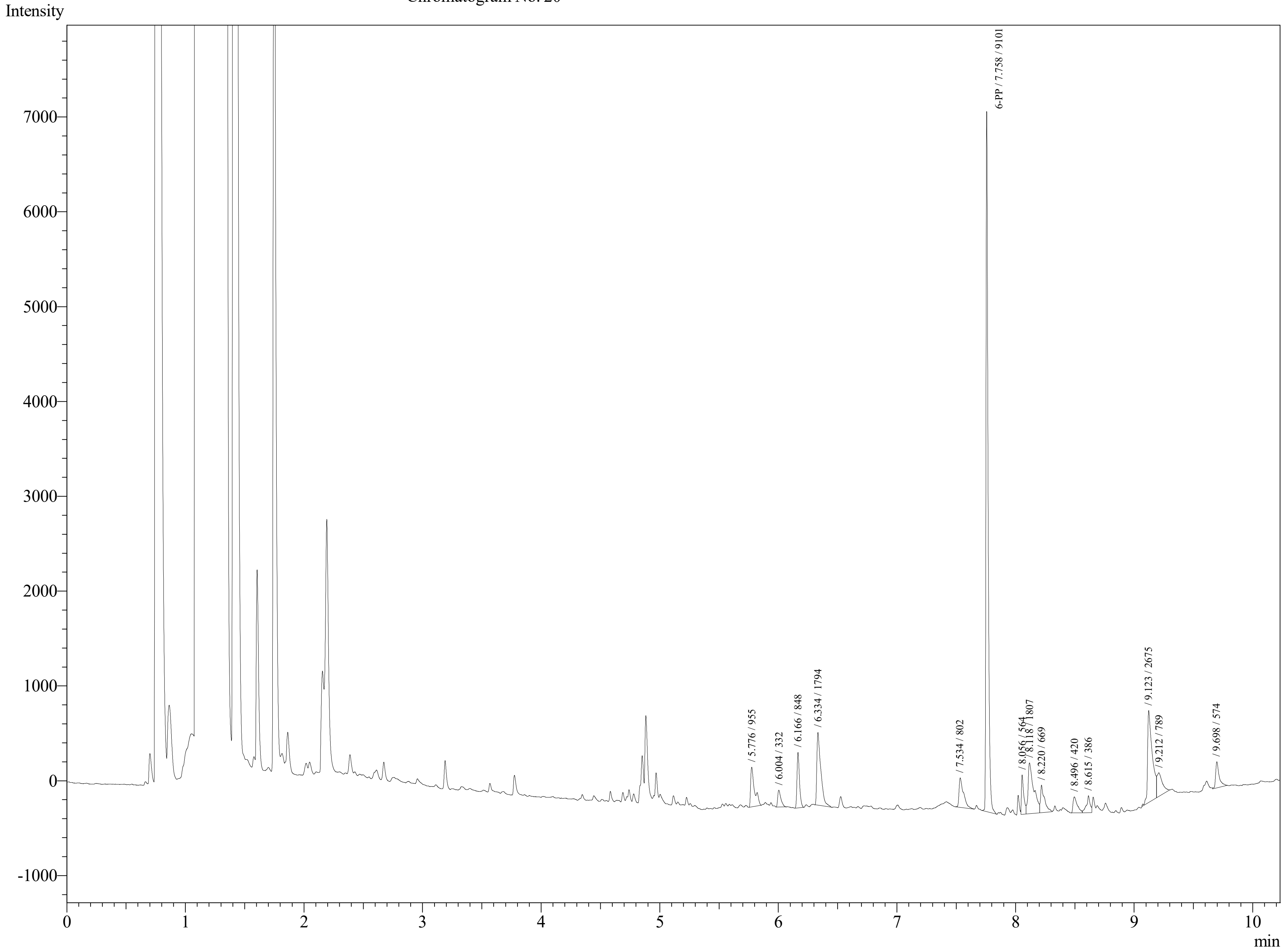

Chromatogram No. 21

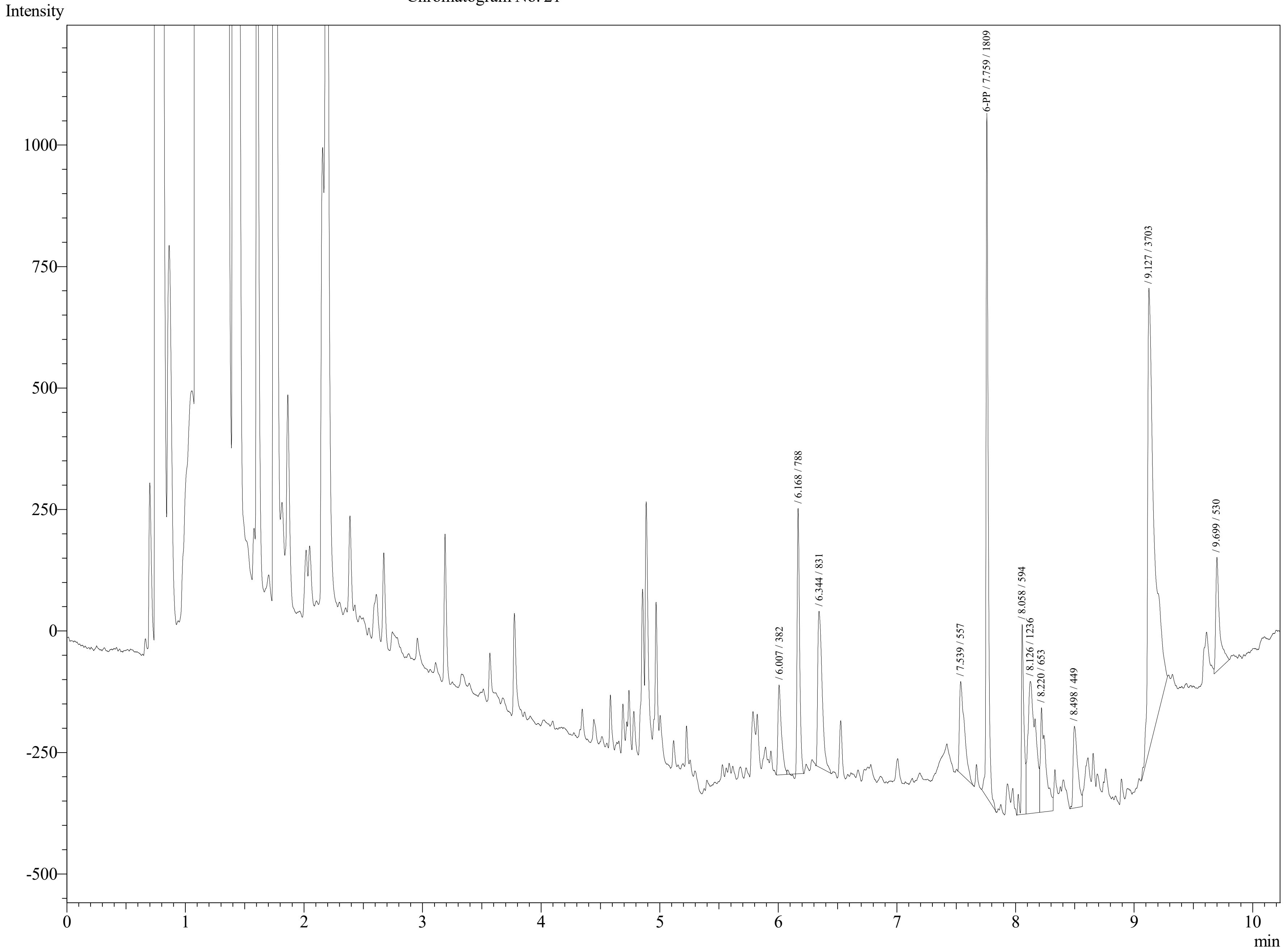

Chromatogram No. 22

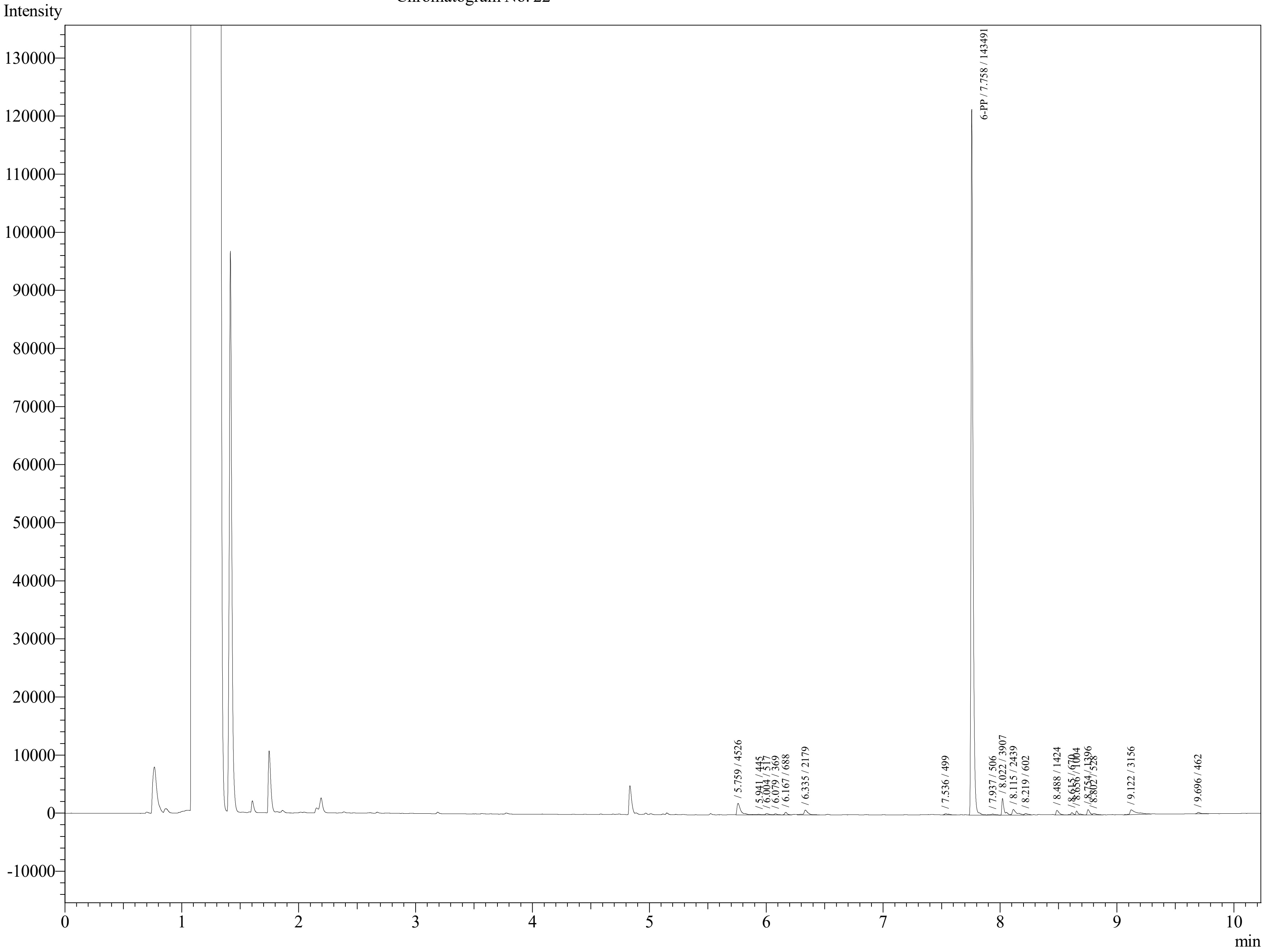

Chromatogram No. 23

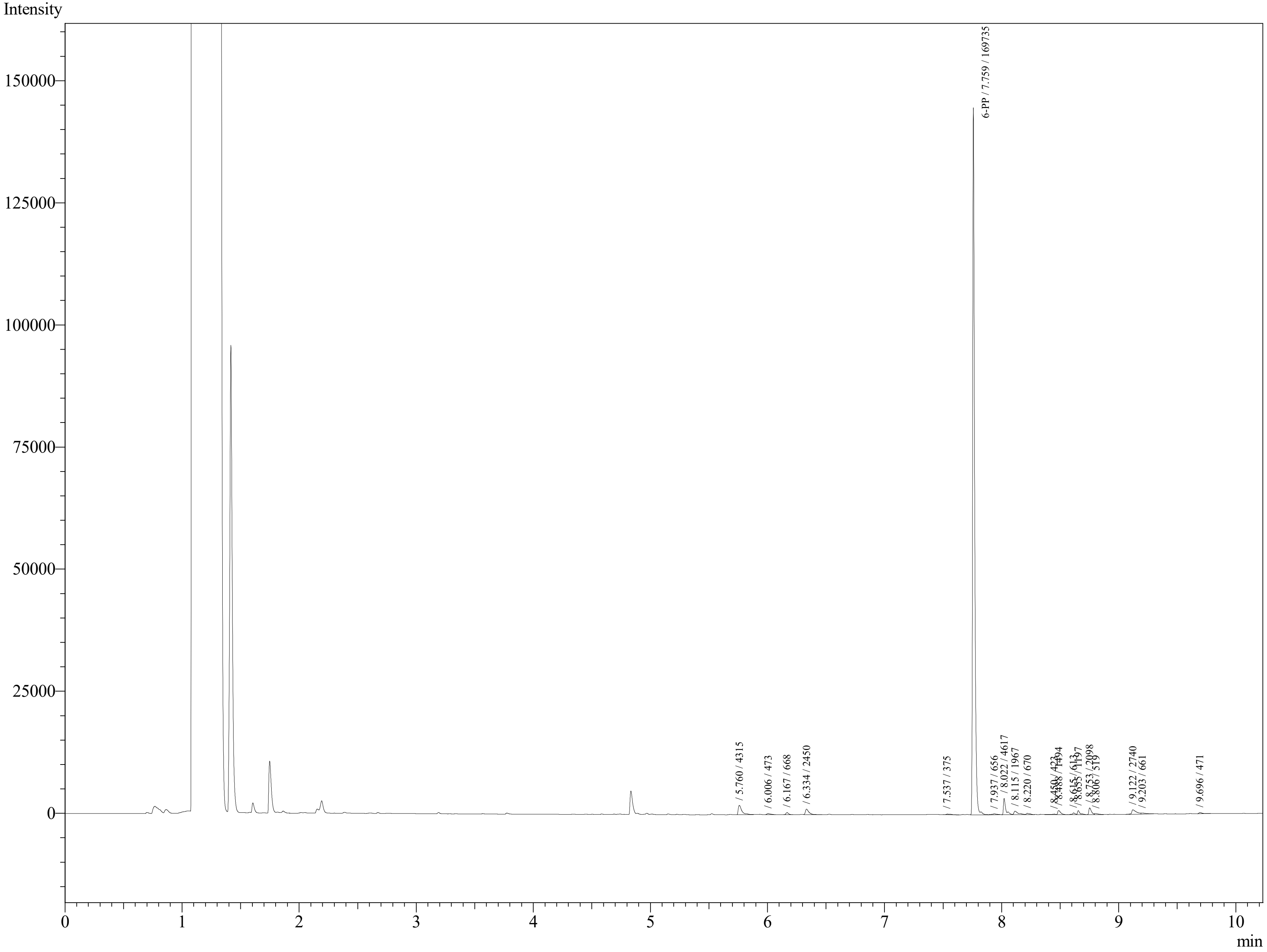

Chromatogram No. 24

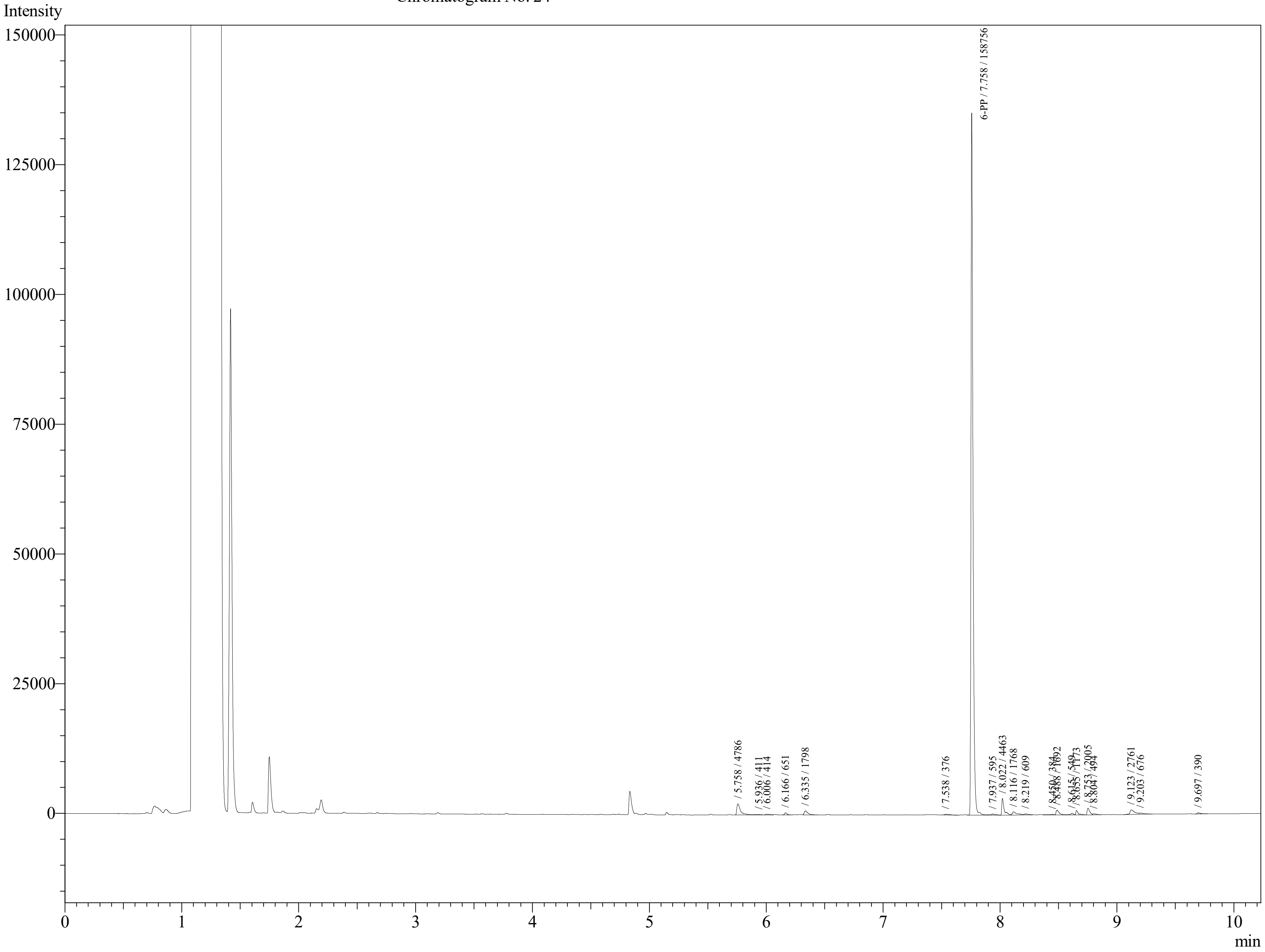

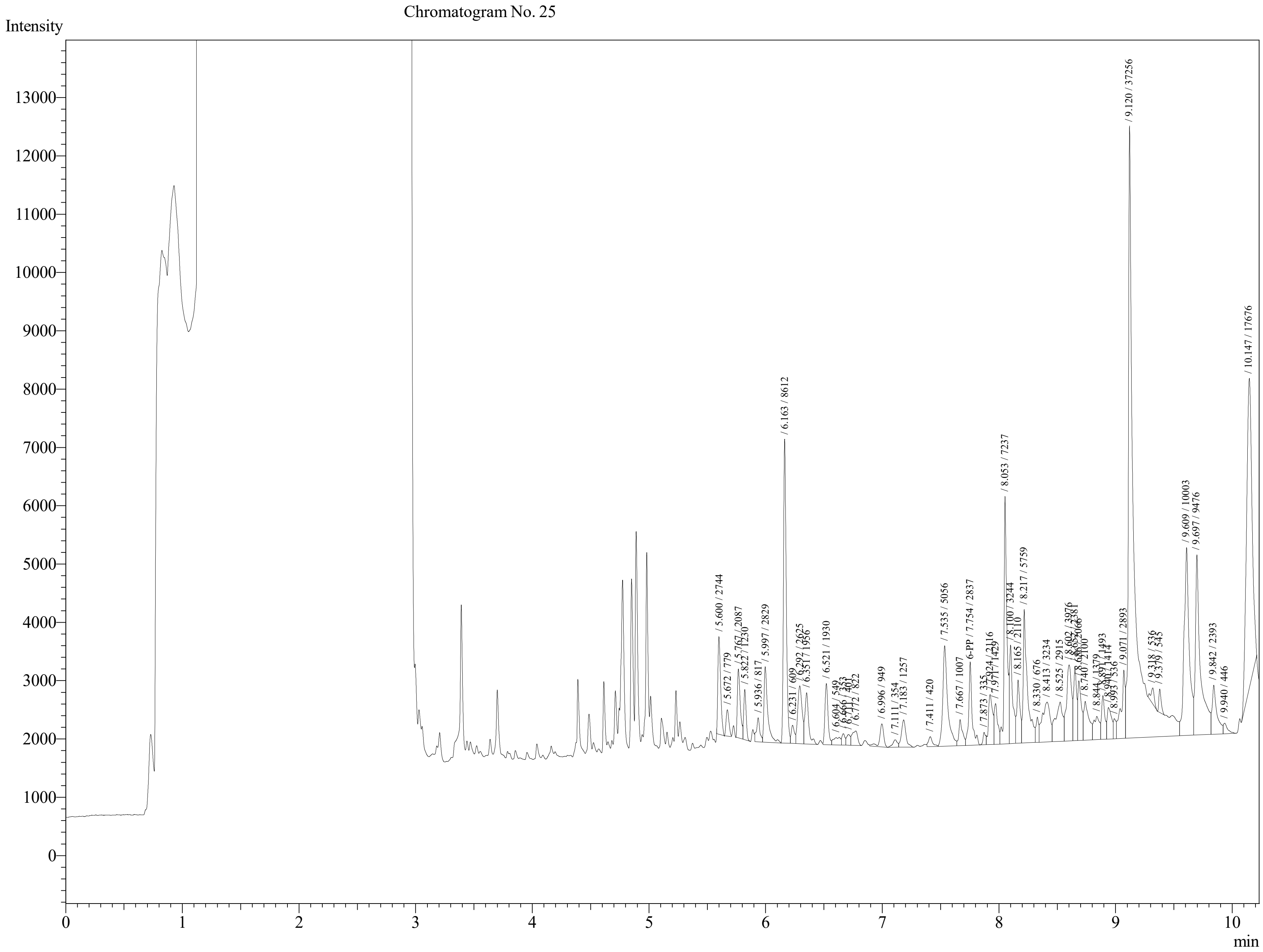

Chromatogram No. 25
Intensity
min
0
1000
2000
3000
4000
5000
6000
7000
8000
9000
10000
11000
12000
13000
0
1
2
3
4
5
6
7
8
9
10
/ 5.600 / 2744
/ 5.672 / 779
/ 5.767 / 2087
/ 5.822 / 1230
/ 5.936 / 817
/ 5.997 / 2829
/ 6.163 / 8612
/ 6.231 / 609
/ 6.292 / 2625
/ 6.351 / 1956
/ 6.521 / 1930
/ 6.604 / 549
/ 6.772 / 822
/ 6.996 / 949
/ 7.111 / 354
/ 7.183 / 1257
/ 7.411 / 420
/ 7.535 / 5056
/ 7.667 / 1007
6-PP / 7.754 / 2837
/ 7.873 / 335
/ 7.924 / 2116
/ 7.971 / 1429
/ 8.053 / 7237
/ 8.100 / 3244
/ 8.165 / 2110
/ 8.217 / 5759
/ 8.330 / 676
/ 8.413 / 3234
/ 8.525 / 2915
/ 8.602 / 3976
/ 8.740 / 2100
/ 8.844 / 1379
/ 8.993 / 536
/ 9.071 / 2893
/ 9.120 / 37256
/ 9.318 / 536
/ 9.379 / 545
/ 9.609 / 10003
/ 9.697 / 9476
/ 9.842 / 2393
/ 9.940 / 446
/ 10.147 / 17676

Chromatogram No. 26

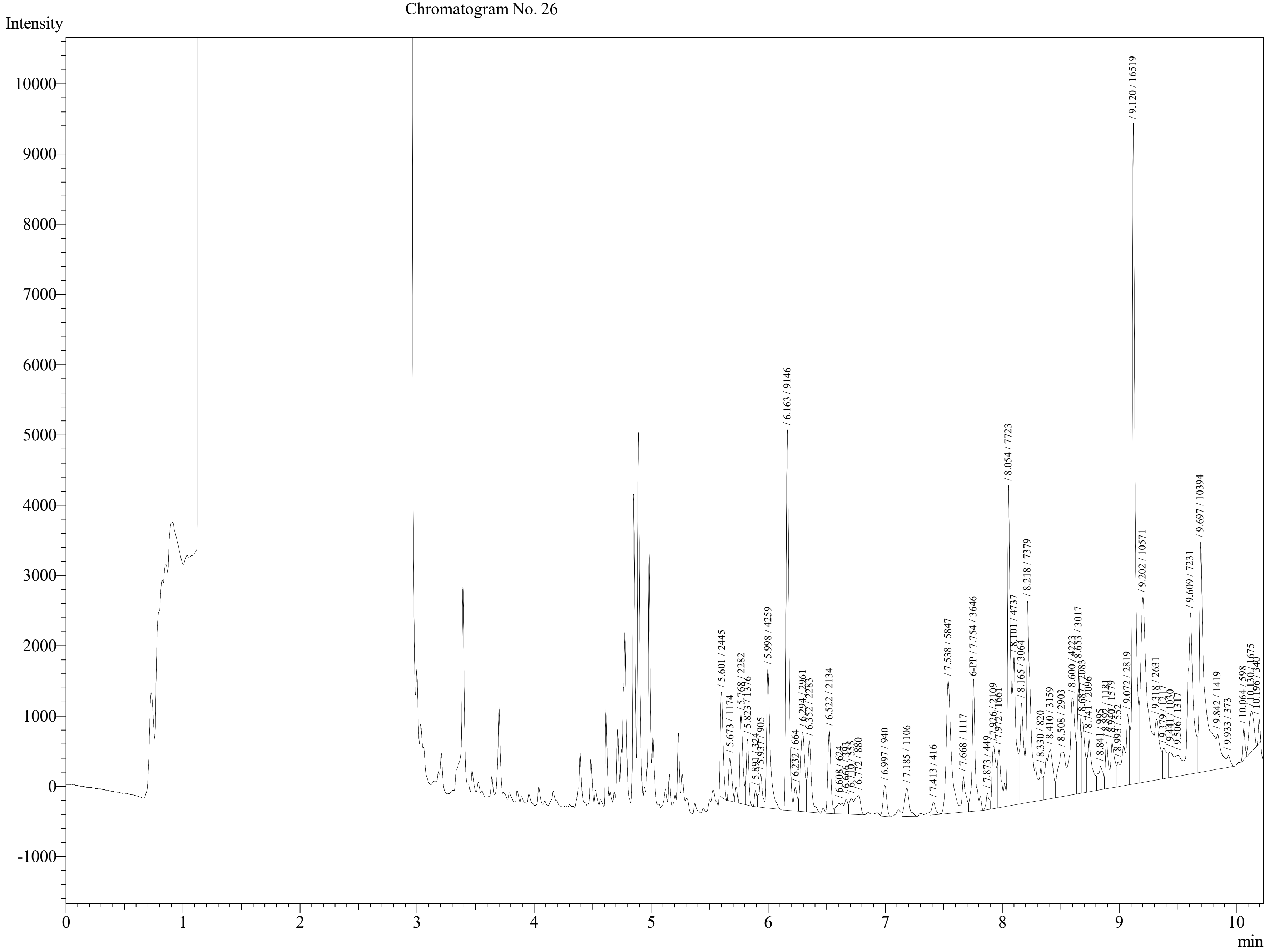

Chromatogram No. 27

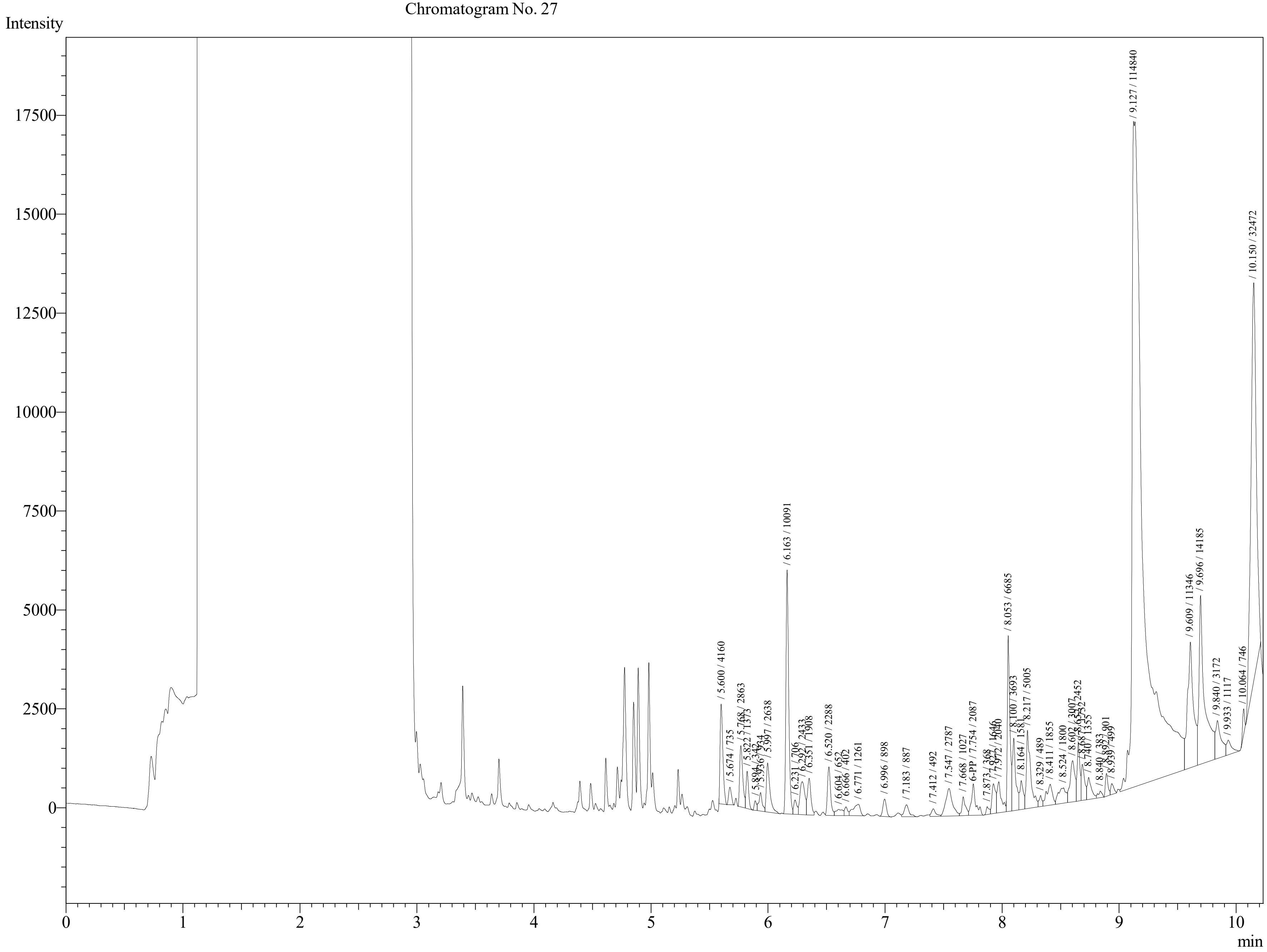

Chromatogram No. 28

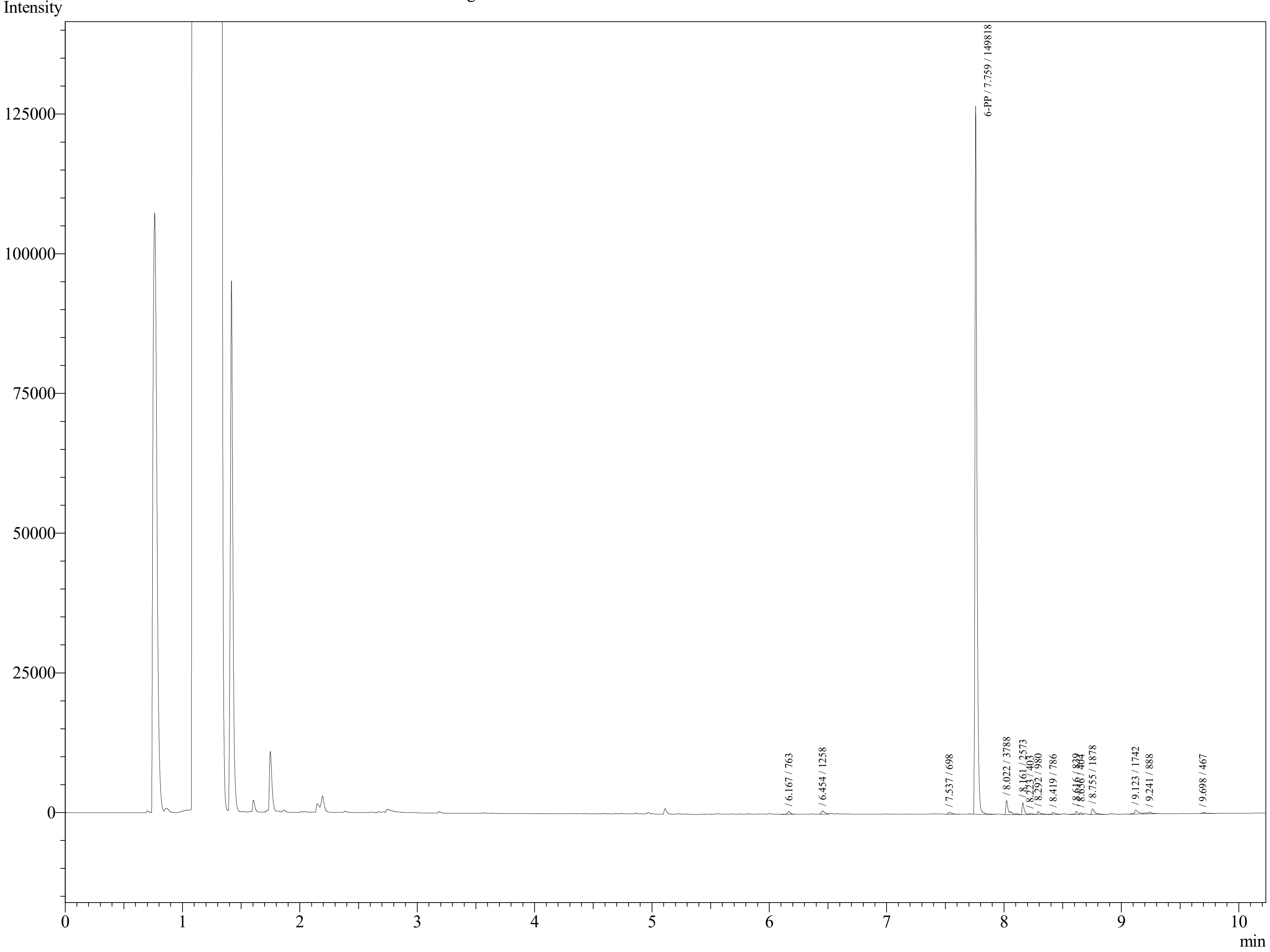

Chromatogram No. 29

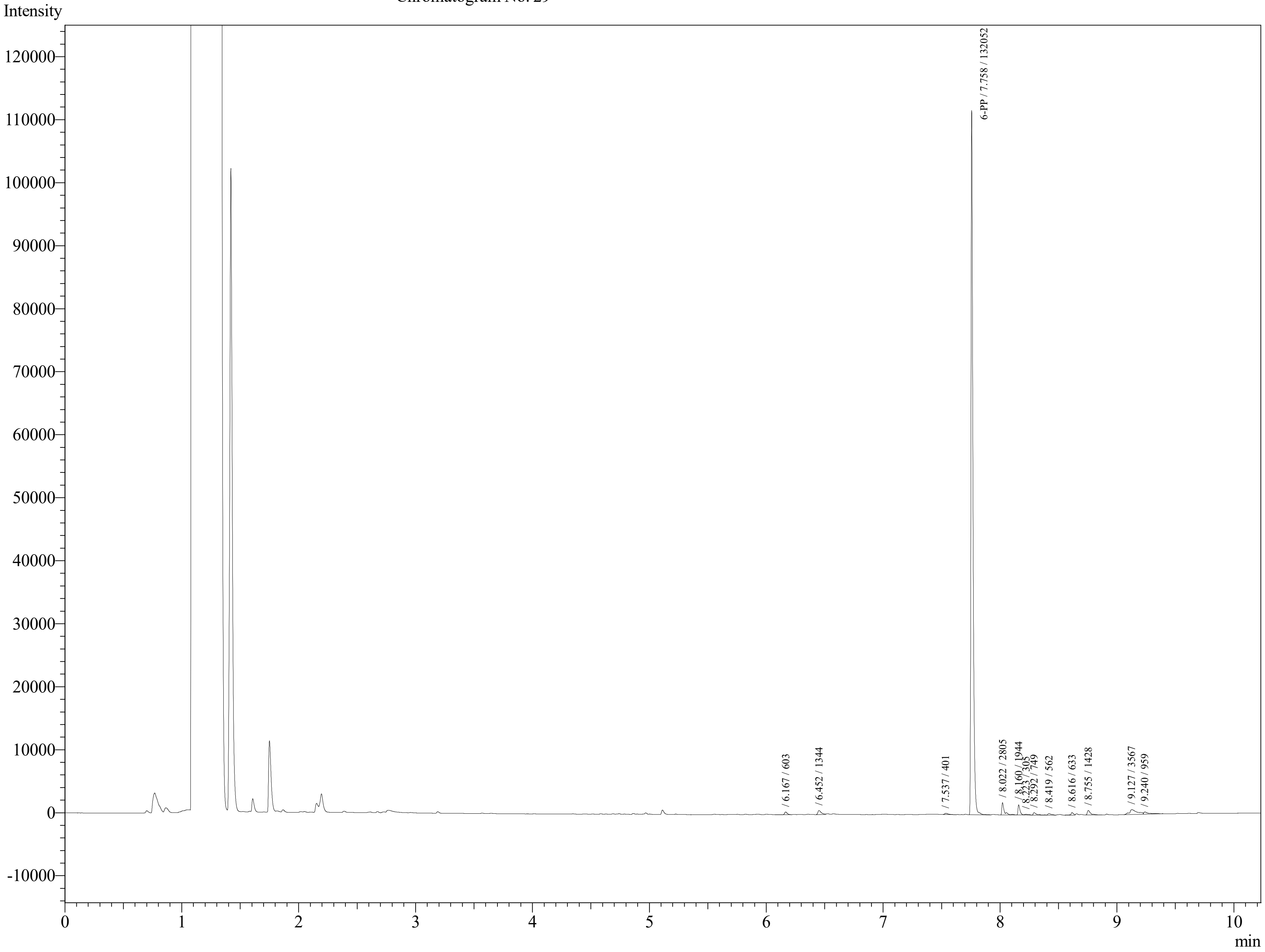

Chromatogram No. 30

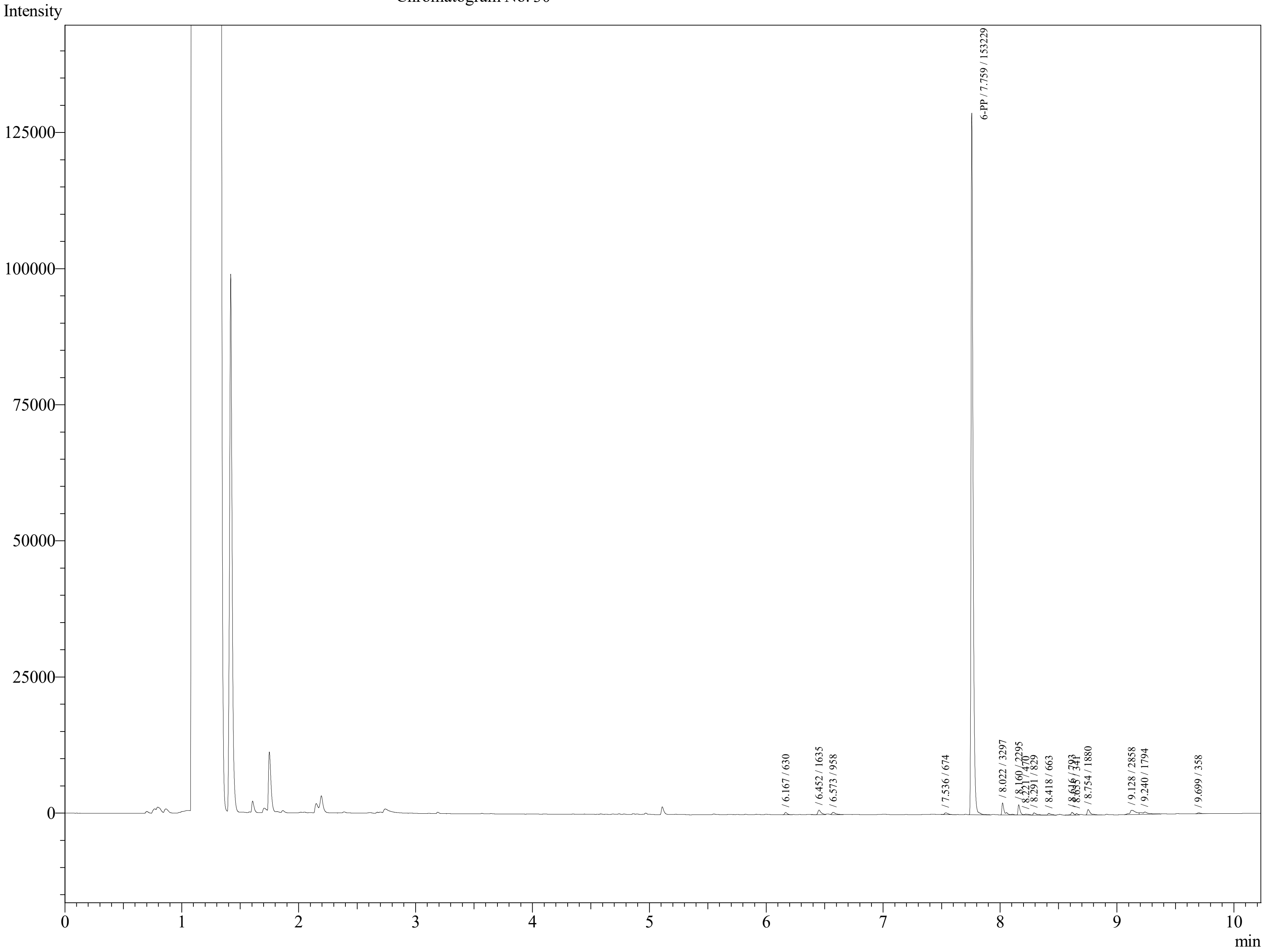

Chromatogram No. 31

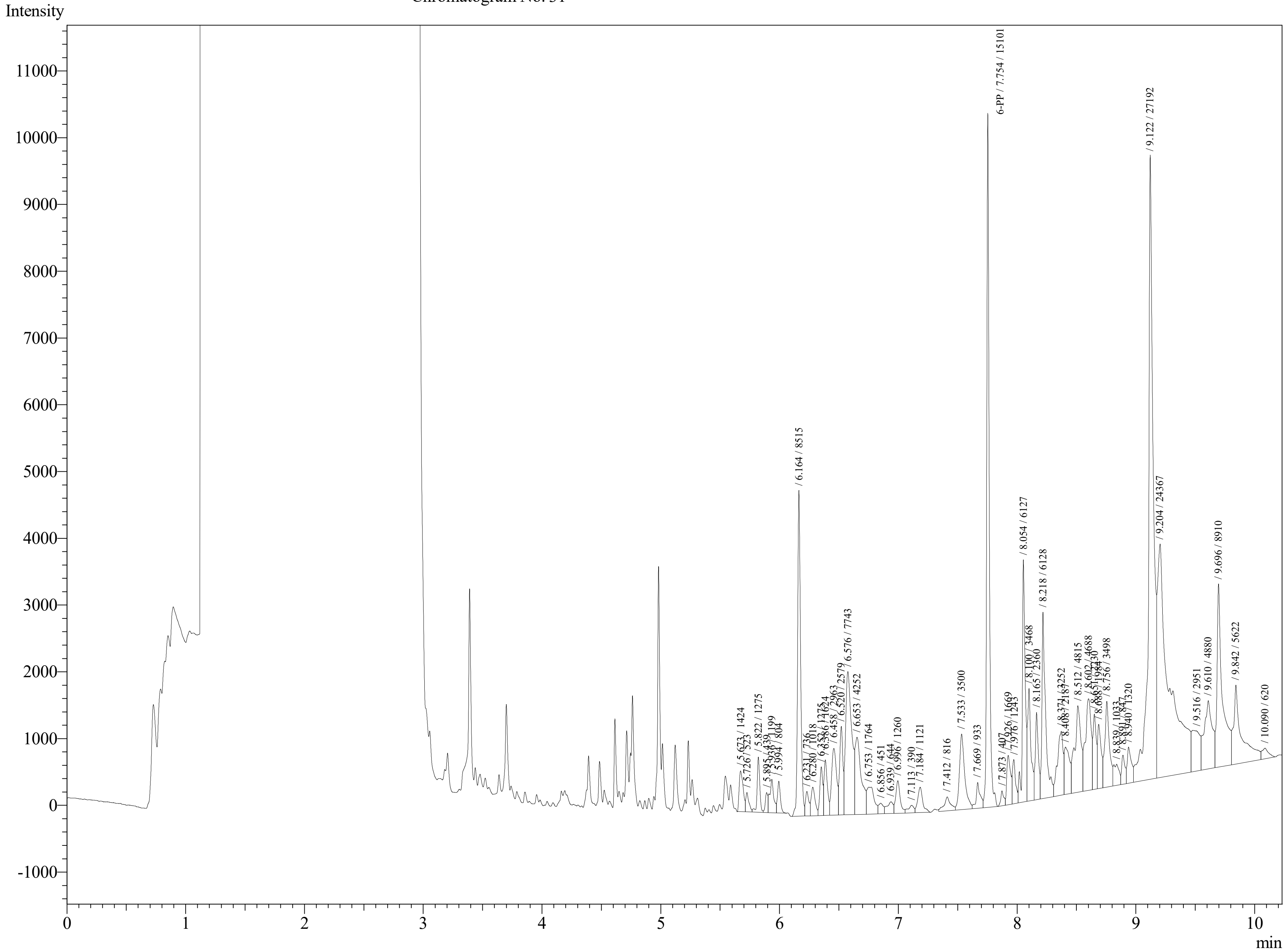

Chromatogram No. 32

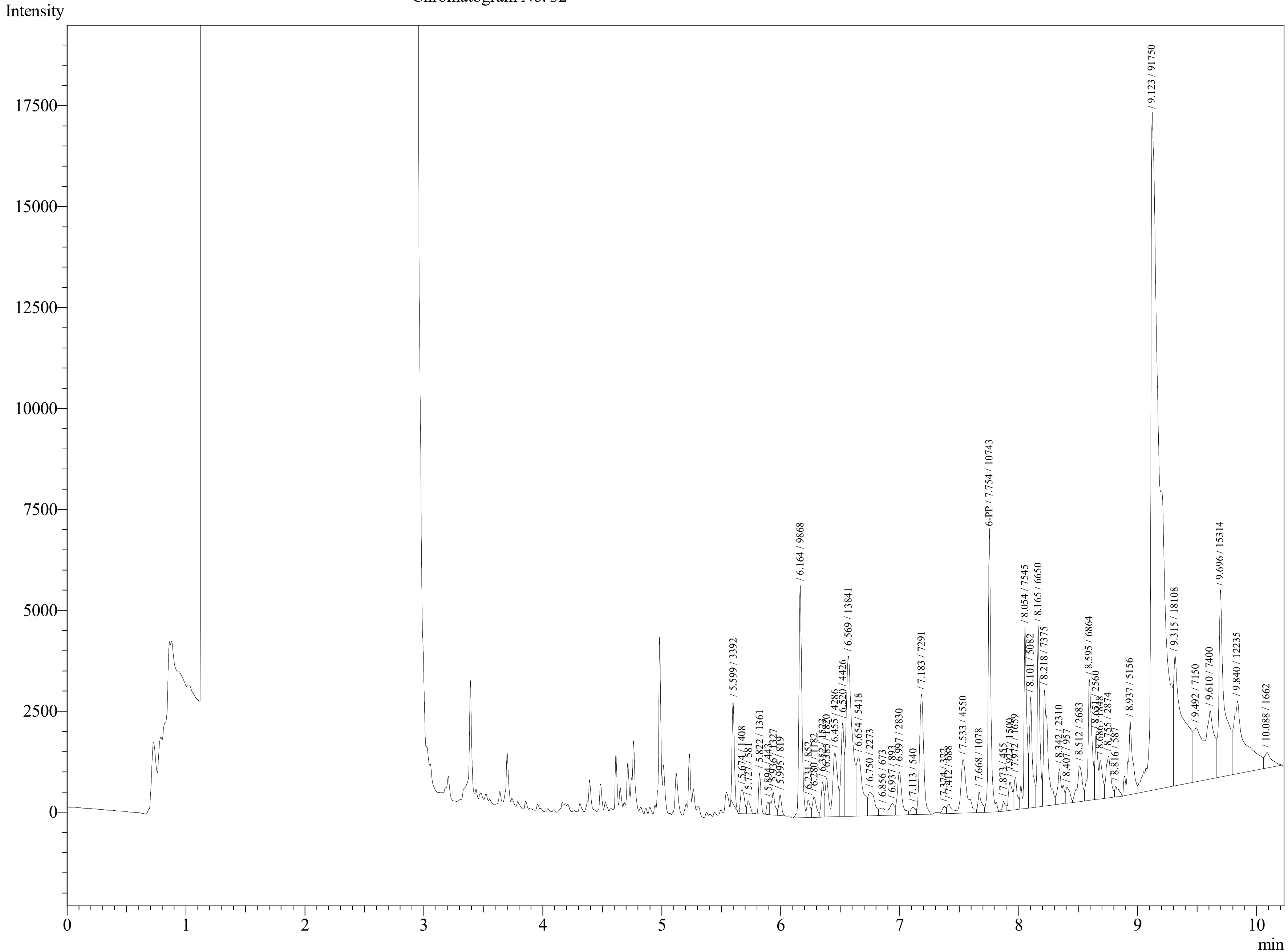

Chromatogram No. 33

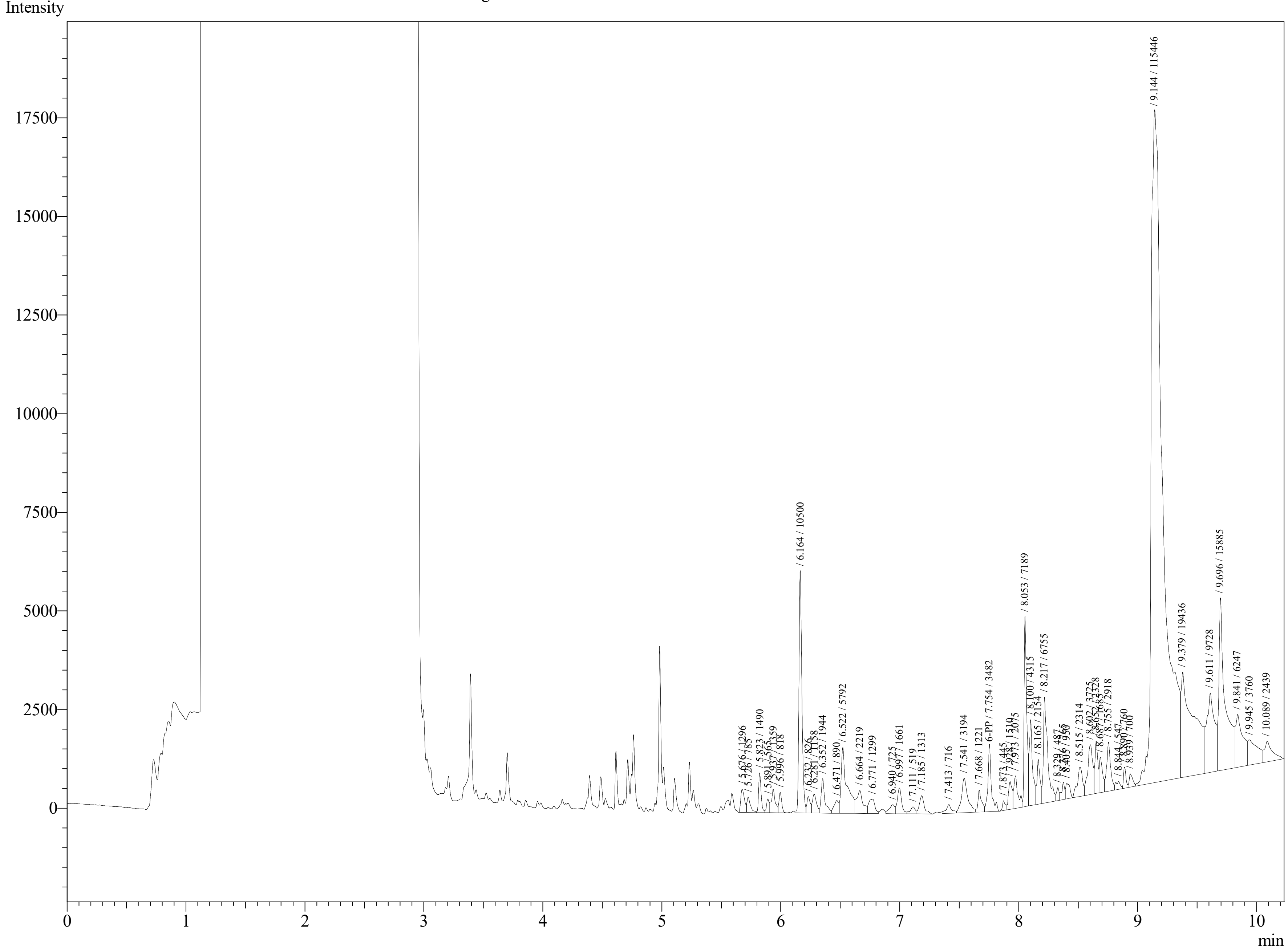

Chromatogram No. 34

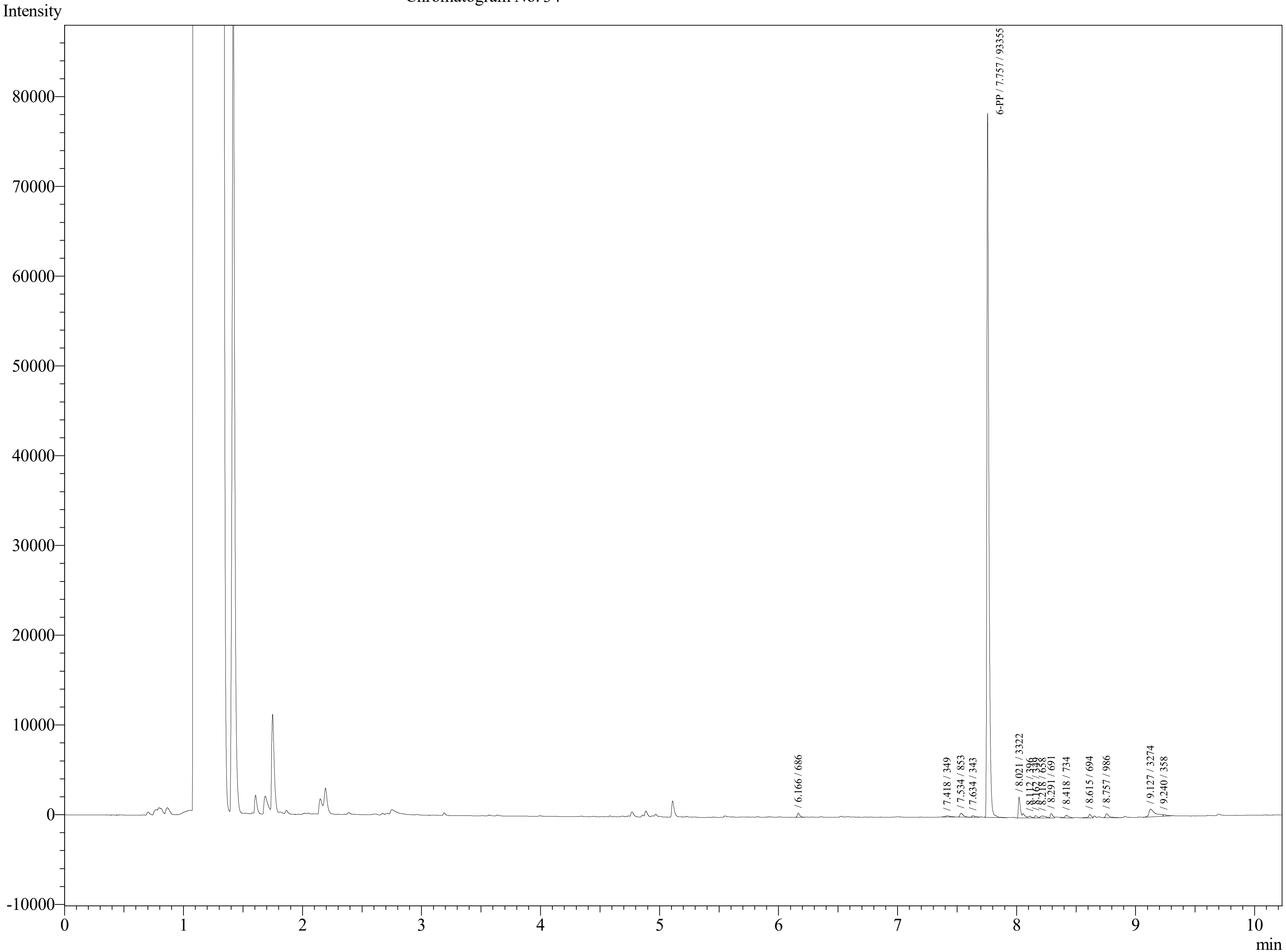

Chromatogram No. 35

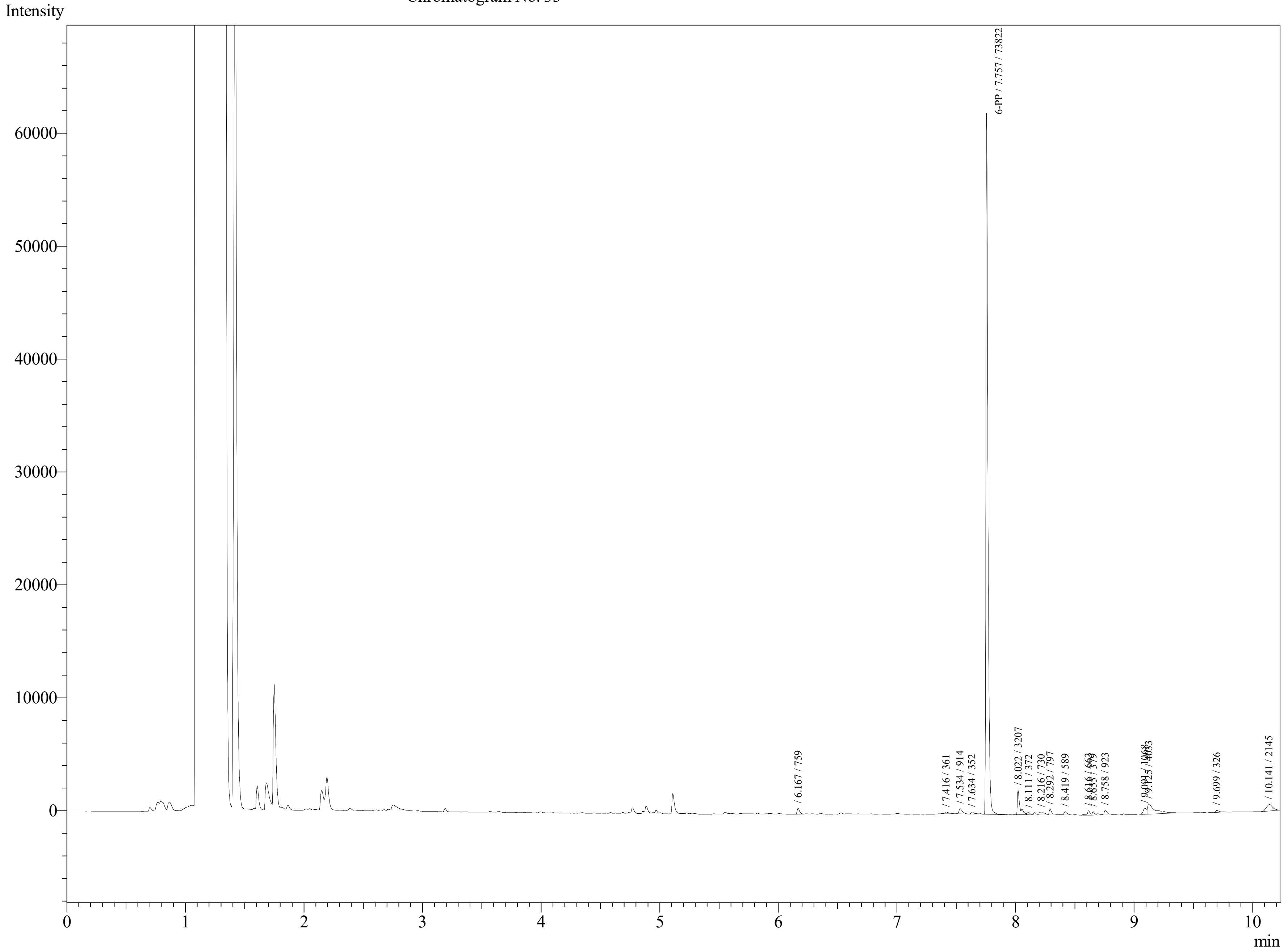

Chromatogram No. 36

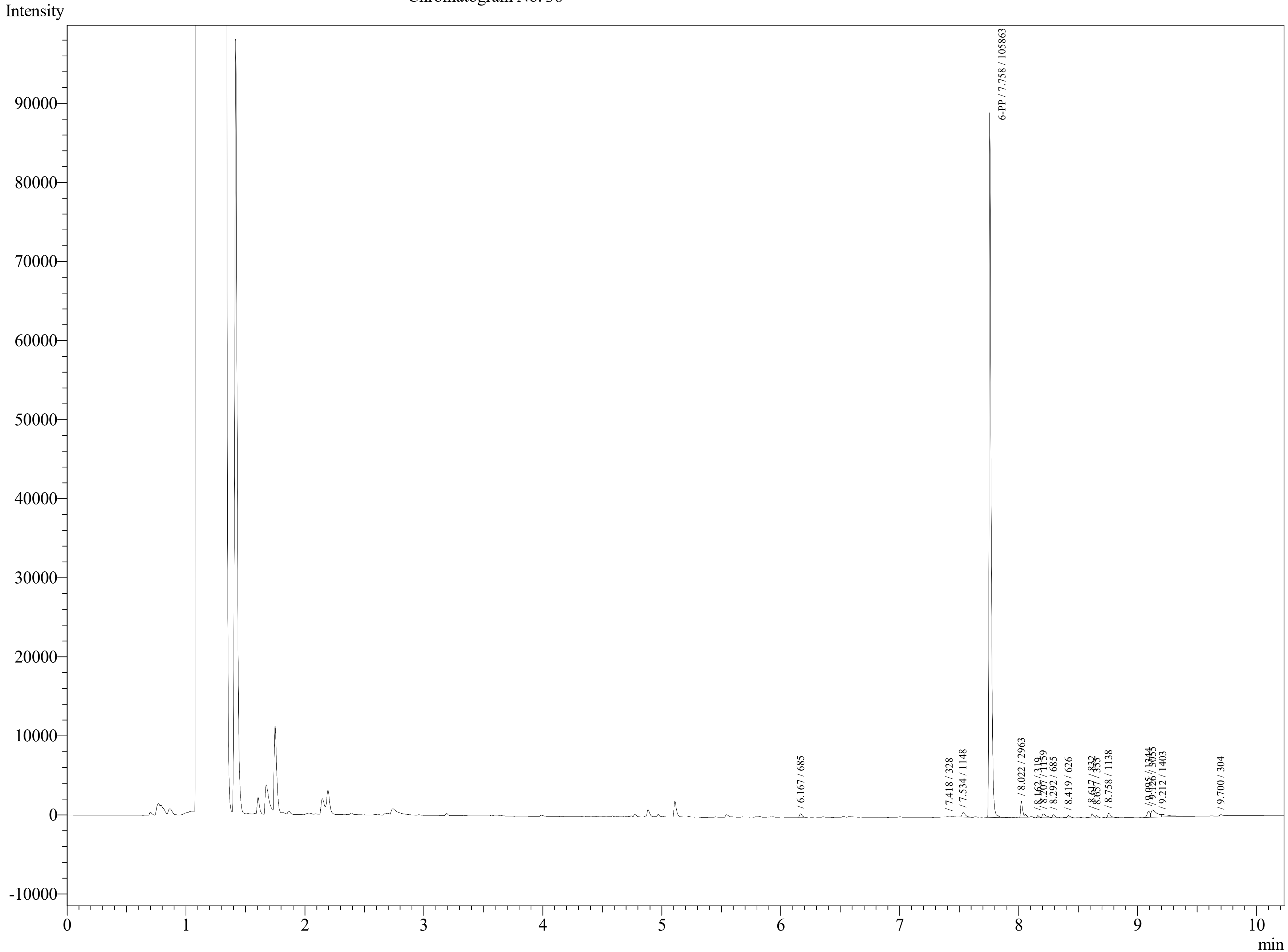

Chromatogram No. 37

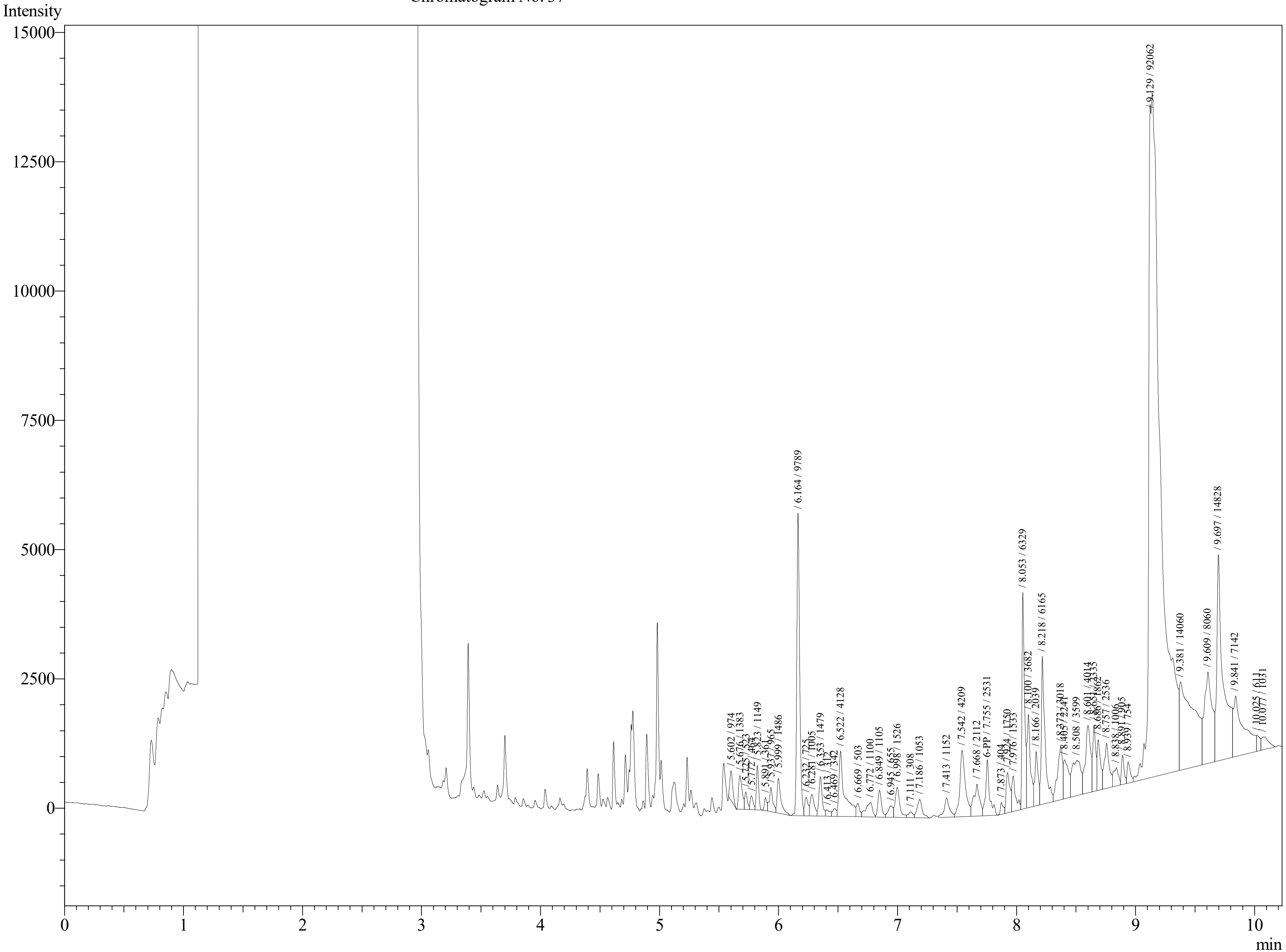

Chromatogram No. 38

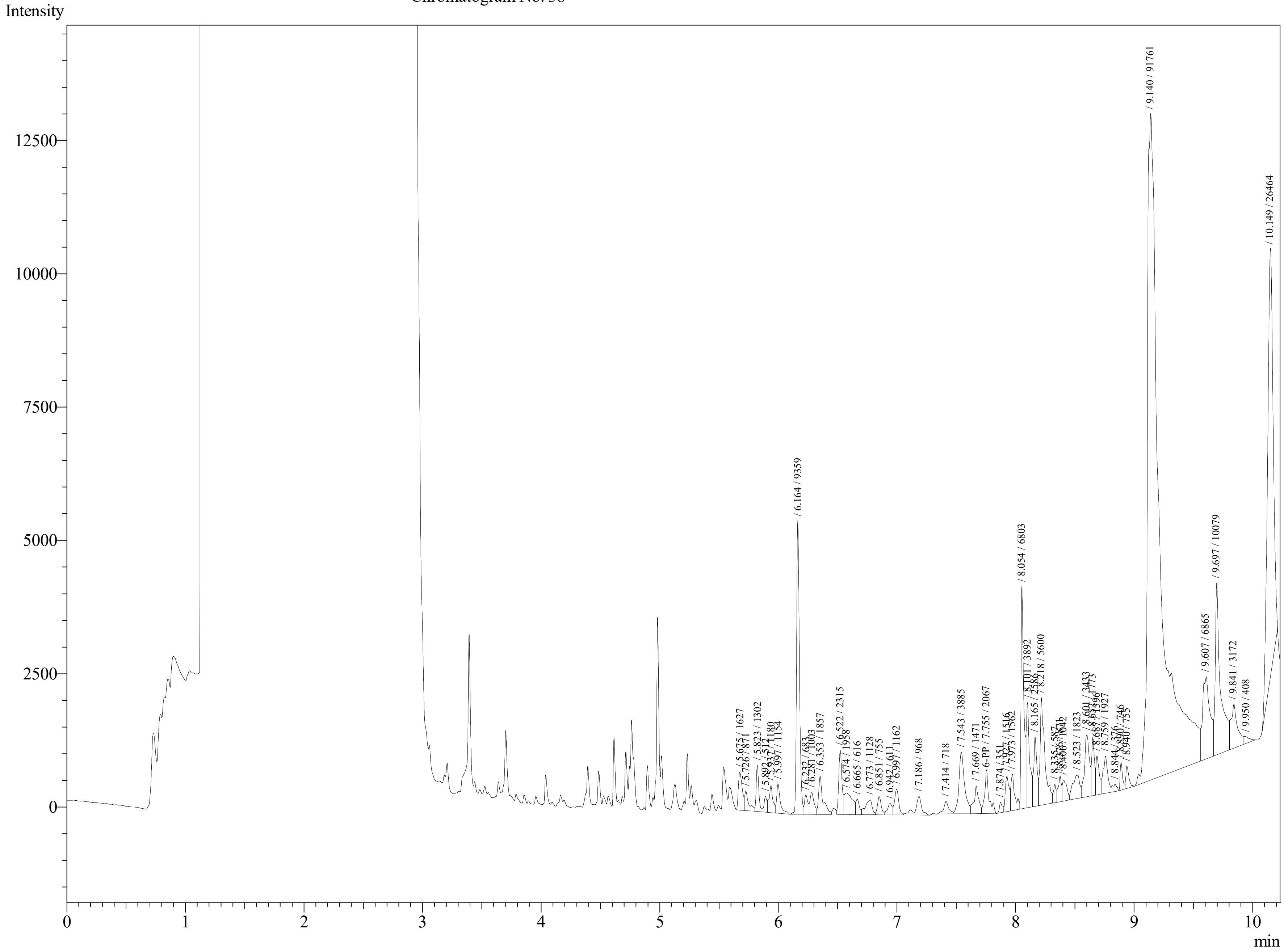

Chromatogram No. 39

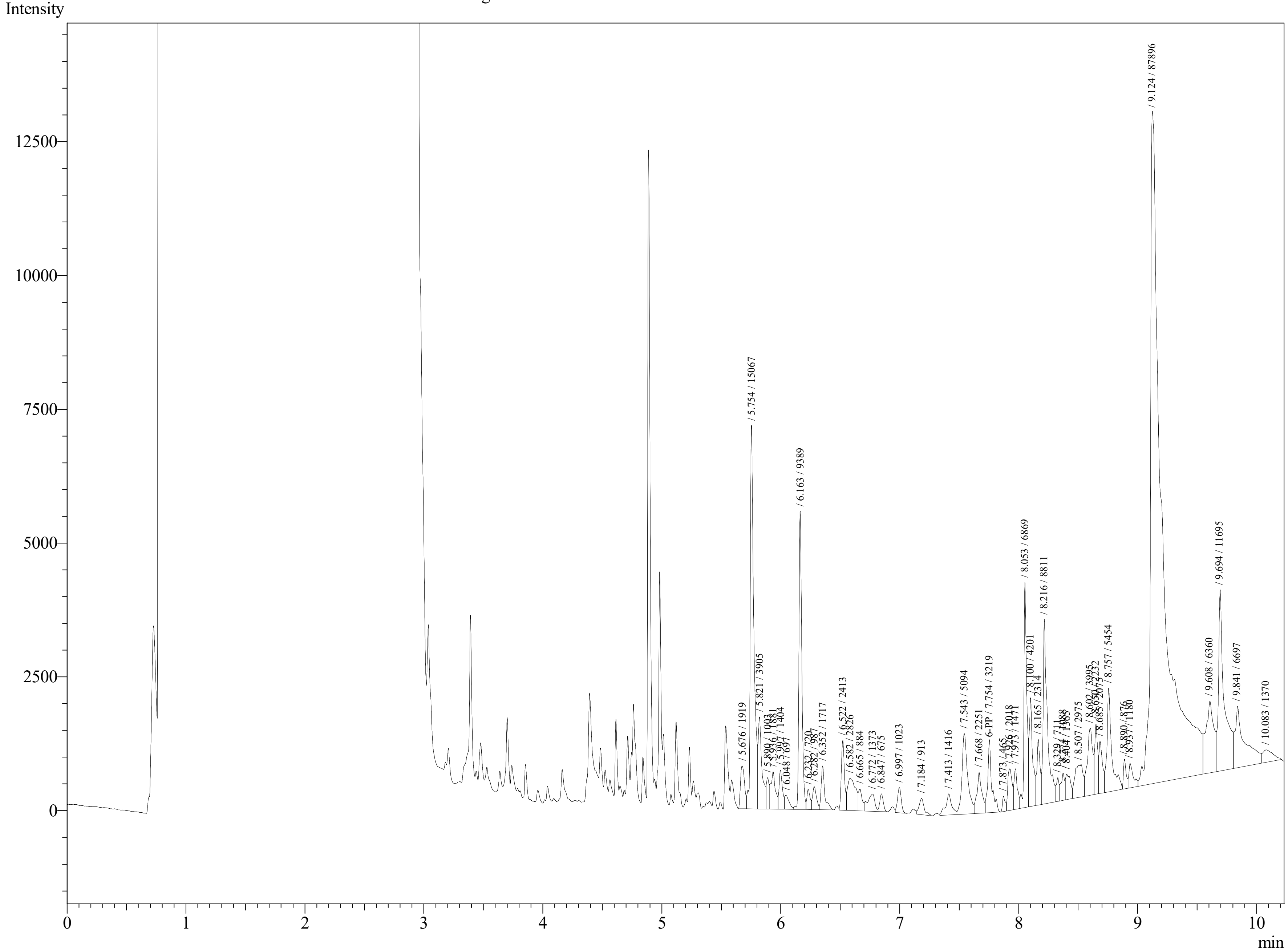

Chromatogram No. 40

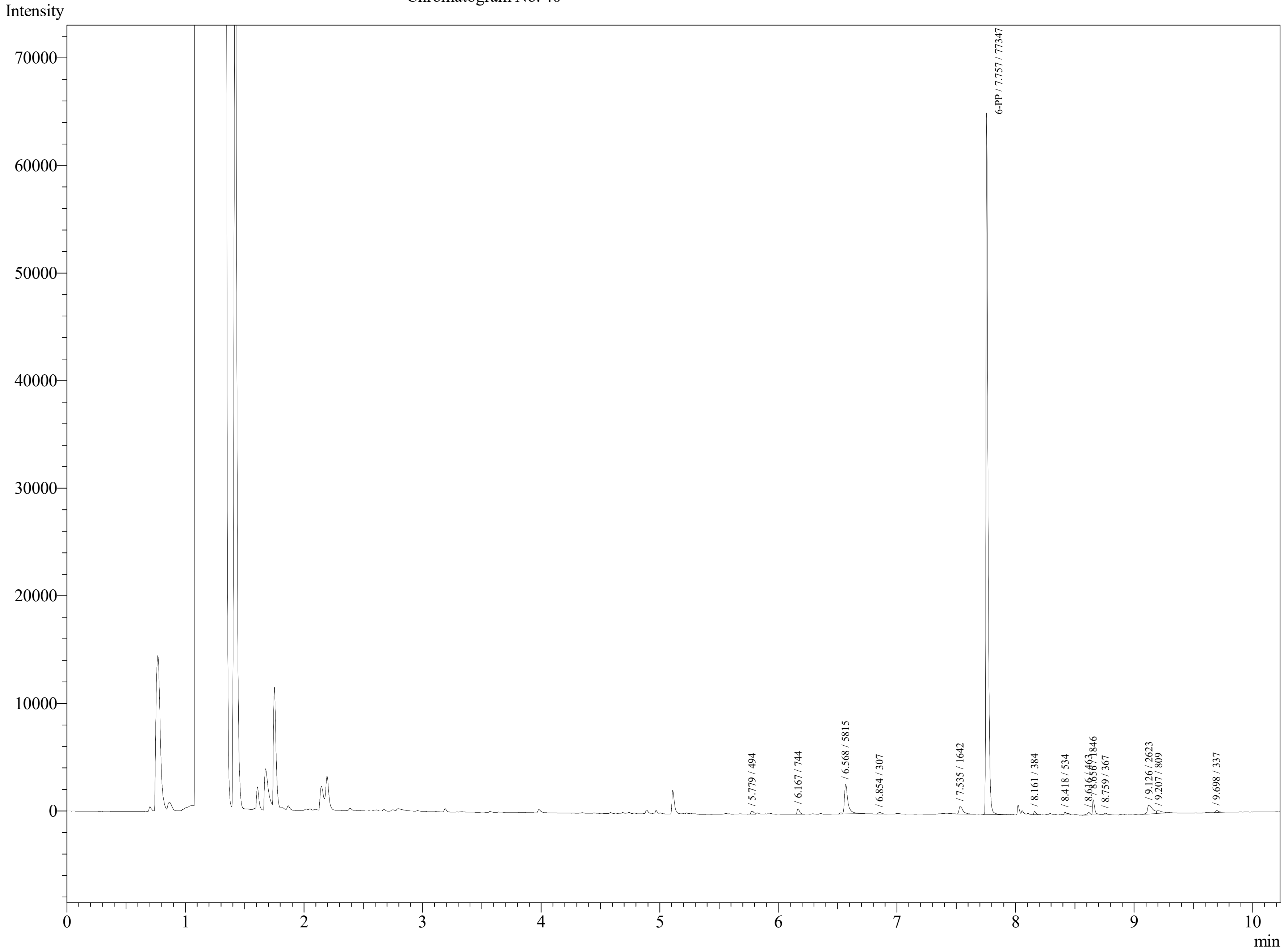

Chromatogram No. 41

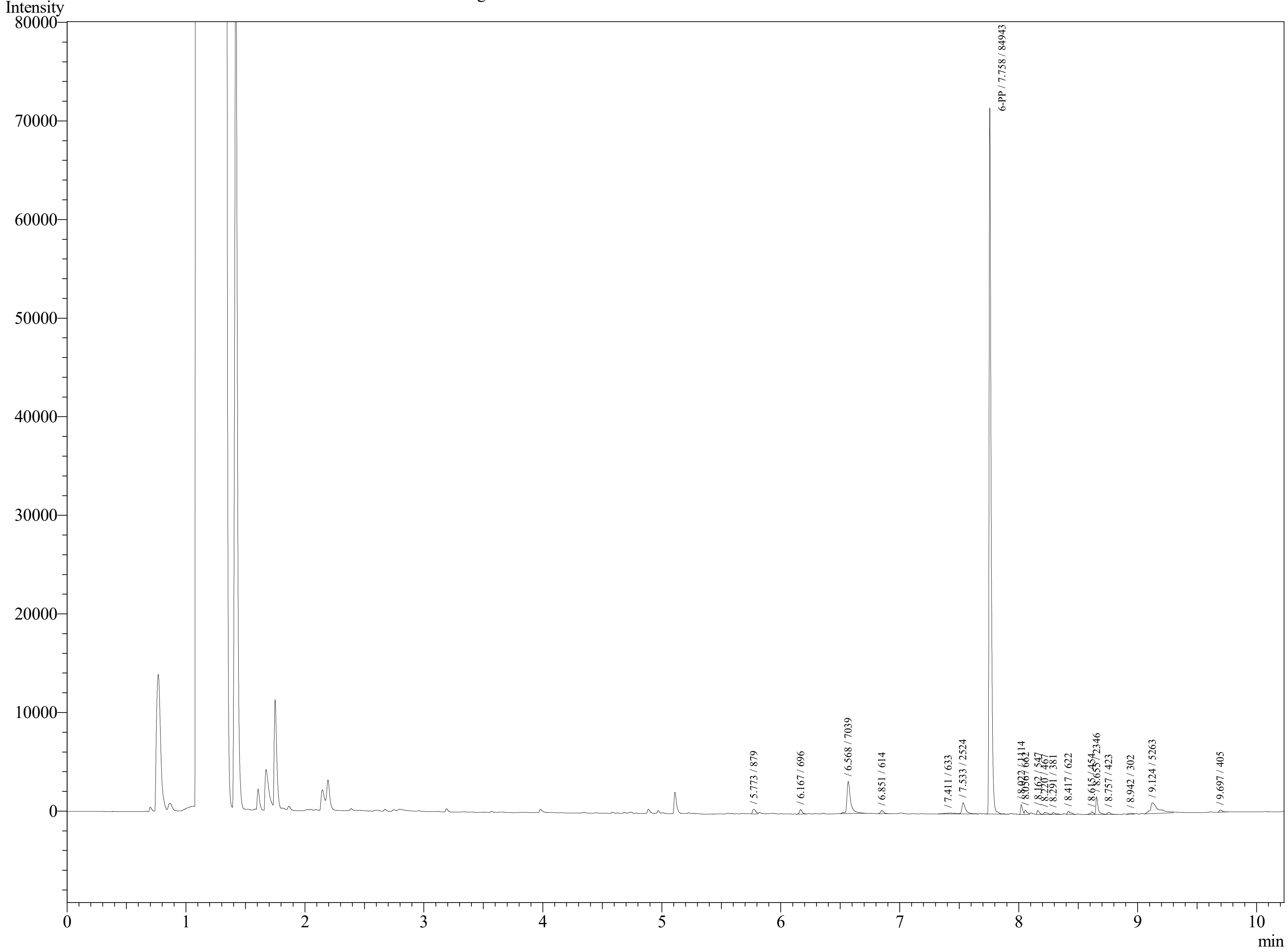

Chromatogram No. 42

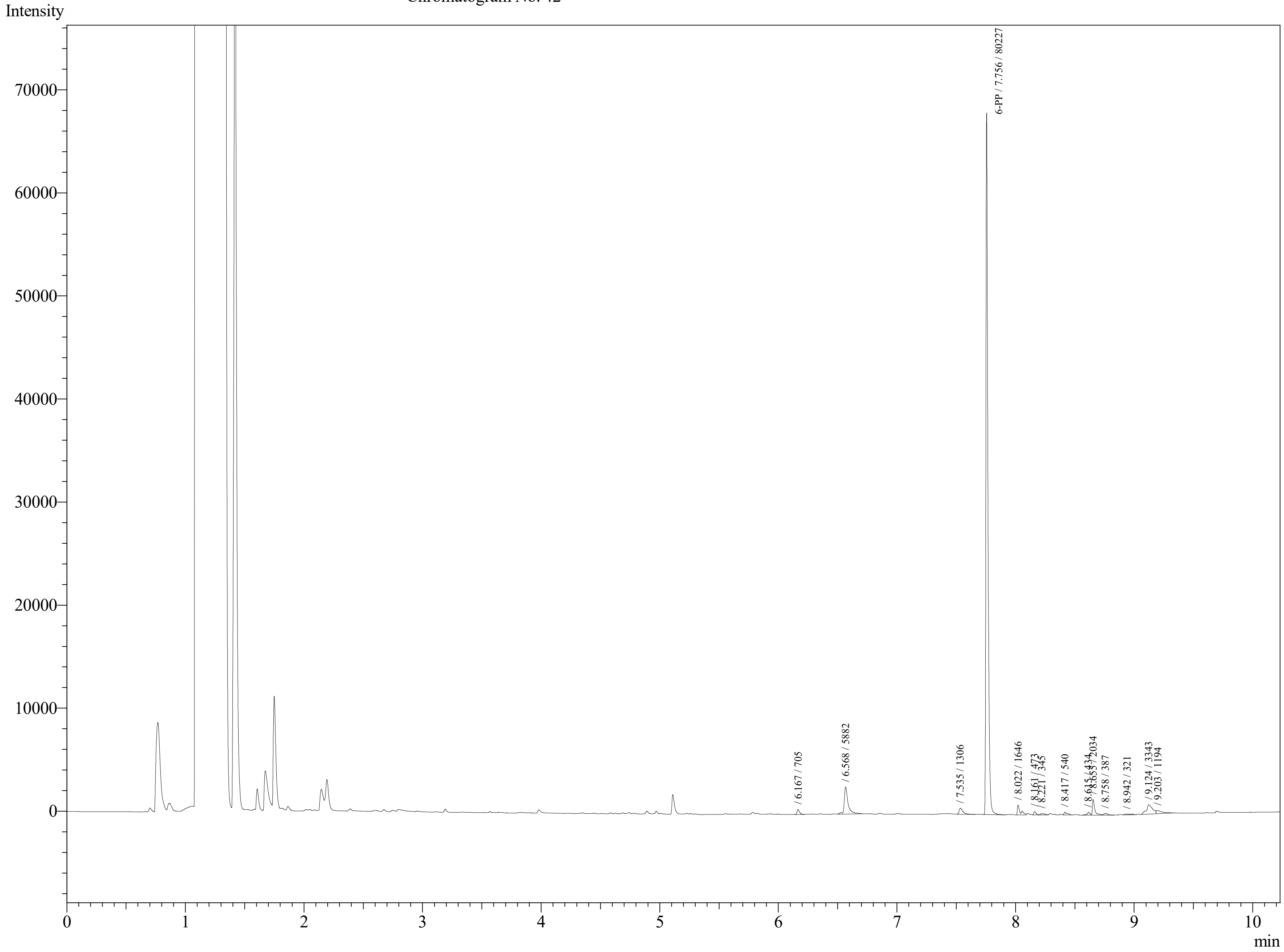

Chromatogram No. 43

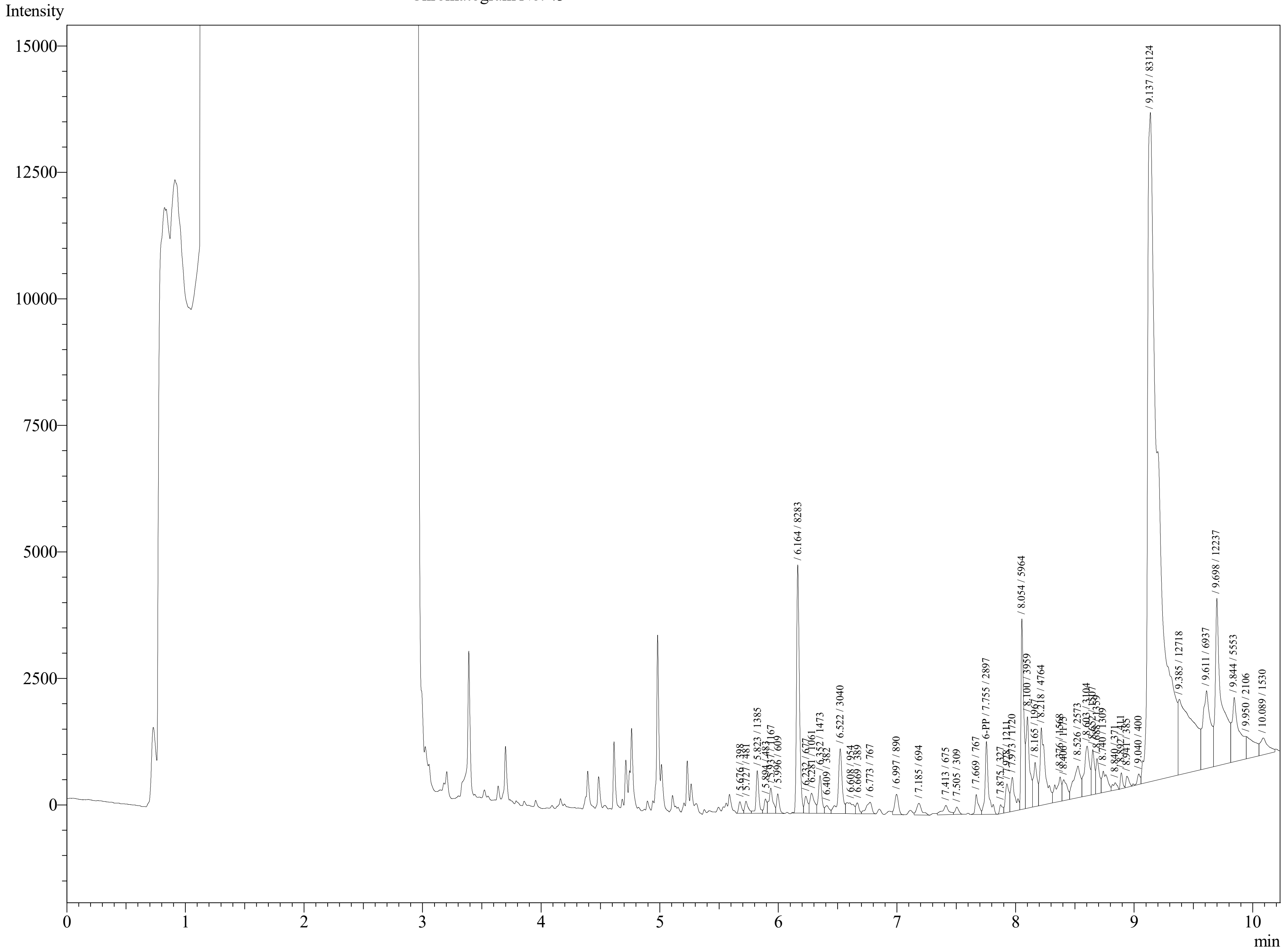

Chromatogram No. 44

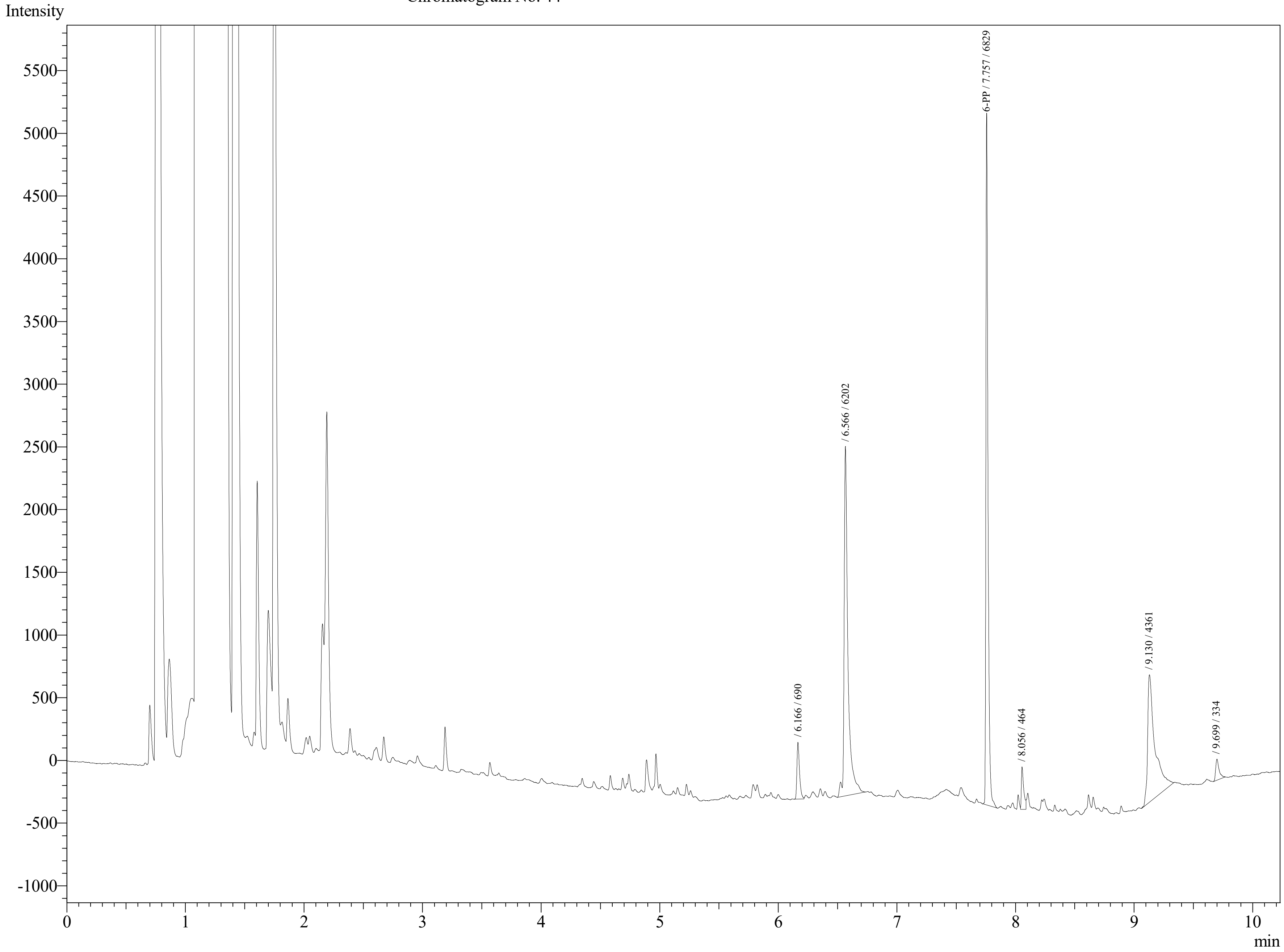

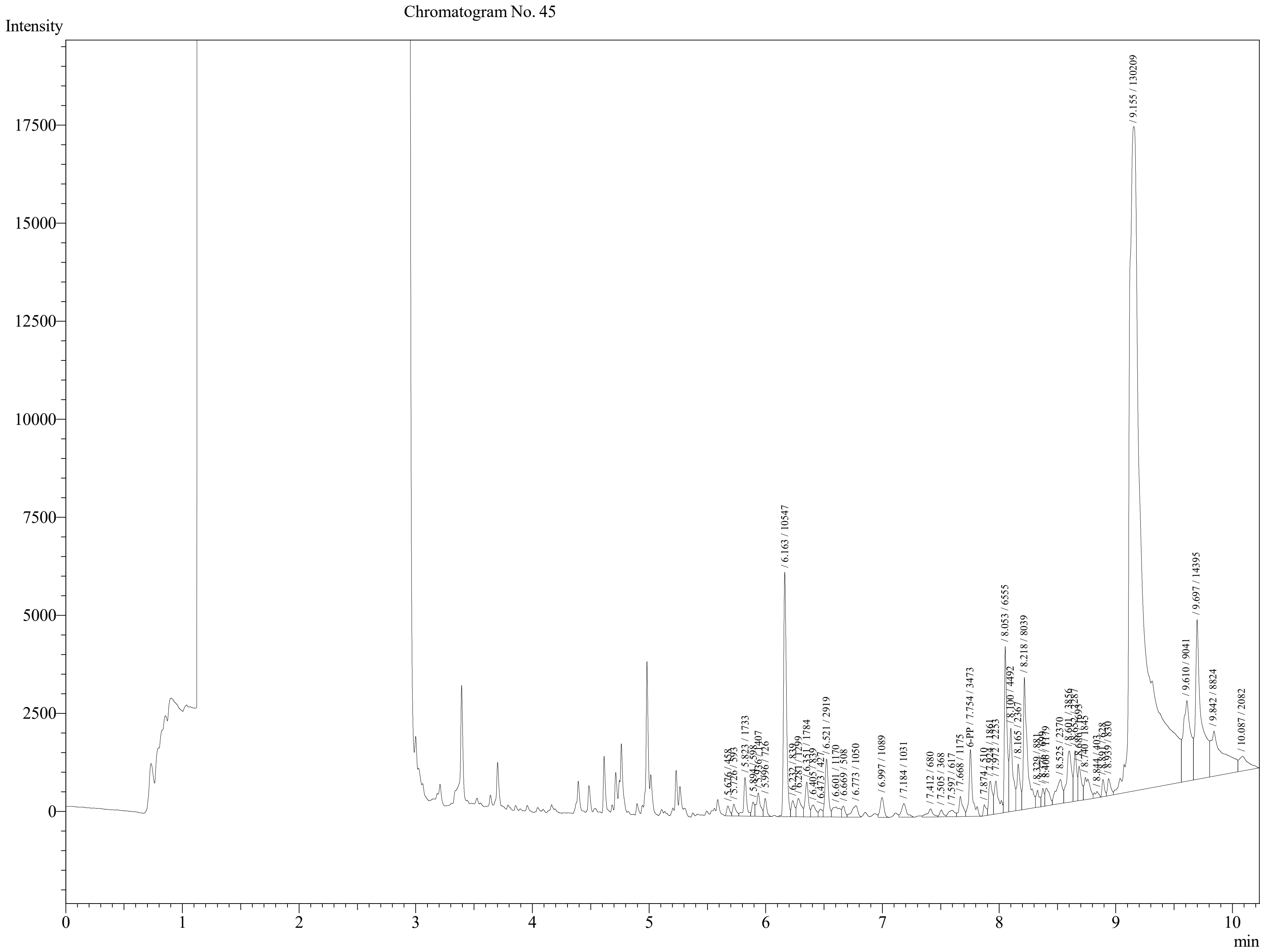

Chromatogram No. 45
Intensity
min
/ 6.163 / 10547
/ 8.053 / 6555
/ 8.218 / 8039
/ 7.754 / 3473
/ 9.155 / 130209
/ 9.610 / 9041
/ 9.697 / 14395
/ 9.842 / 8824
/ 10.087 / 2082
