# Supplementary material for: Increase in 6-pentyl-alpha-pyrone production by Trichoderma spp. in liquid medium using in situ removal by adsorbent resin
Source: Nat Prod Bioprospect. 2026 Oct 19;16(1):93. doi: 10.1007/s13659-026-00650-y (PMC13680593; doi:10.1007/s13659-026-00650-y)
Supplement: Supplementary file 2 [file 13659_2026_650_MOESM1_ESM.docx]

# **Increase in 6-pentyl-alpha-pyrone production by *Trichoderma* spp. in liquid medium using *in situ* removal by adsorbent resin**

Natural Products and Bioprospecting

Alex Maan

Lincoln University, Department of Pest Management and Conservation, Canterbury, New Zealand

ORCID 0009-0009-6476-2884

Eirian Jones

Lincoln University, Department of Pest Management and Conservation, Canterbury, New Zealand

Eirian Jones: 0000-0002-1879-4537

John Hampton

Lincoln University, Department of Agricultural Sciences, Canterbury, New Zealand

ORCID 0000-0003-3449-825X

Travis Glare

Lincoln Agritech, Canterbury, New Zealand

ORCID 0000-0001-7795-8709

Artemio Mendoza-Mendoza

Lincoln University, Department of Pest Management and Conservation, Canterbury, New Zealand

ORCID 0000-0002-1532-3406

**Supplementary material**

Supplementary Fig. 1	Thin-layer chromatography (TLC) plate photographed under 254 nm wavelength with supernatant samples from 10 Trichoderma isolates cultivated in PDB for 7 days. Lanes are ordered left to right from the lowest producer to the highest producer of 6-PP according to spectrophotometry measurements with a 6-PP standard on the right-most lane indicated by the white arrow.

Supplementary Fig. 2	TLC analysis of Trichoderma supernatants and 6-PP reference 320 nm scanned with a CAMAG scanner. Scans showing absorbance of TLC plate with supernatant samples from T. atroviride LU132, T. atroviride LU497, T. gamsii LU771, T. gamsii LU775, T. viridescens LU1369, and a reference 6-PP sample at 320 nm wavelength. A small peak due to the graphite pencil mark can be seen at 0.0 RF, indicated by dashed rectangles does not represent sample absorbance. All other peaks represent sample absorbance. The reference sample of 6-PP is indicated.

6-pentyl-alpha-pyrone

6-heptyl-5,6-dihydro-2H-Pyran-2-one

(E)-6-Pent-1-enyl pyran-2-one

δ-decanolactone

δ-Undecalactone

Massoilactone

Tetrahydro-4-hydroxy-6-pentyl-2H-pyran-2-one

2H-Pyran-2,4(3H)-dione, 3-acetyl-6-methyl-

Supplementary Fig. 3	Chemical structures of pyrones produced by Trichoderma spp. that accumulated in Amberlite as identified by GC-MS.

| **Supplementary Table 1.** GC-MS chromatograms of *Trichoderma atroviride* IMI206040 samples cultured in Potato Dextrose Broth (PDB) for 5 days. | | | |
| --- | --- | --- | --- |
| **Name** | **Ret Time** | **Area** | **Area %** |
| 1,2-Ethanediol, monoacetate | 6.518 | 49148 | 0.01 |
| 2-Furanmethanol | 6.587 | 760392 | 0.09 |
| 2-Heptanone | 7.093 | 121882 | 0.01 |
| Furan, 2-pentyl- | 8.769 | 93375 | 0.01 |
| 2-Cyclopenten-1-one, 2-hydroxy-3-methyl- | 9.479 | 76792 | 0.01 |
| Furaneol | 9.97 | 97164 | 0.01 |
| Ethanone, 1-(1H-pyrrol-2-yl)- | 10.038 | 527966 | 0.06 |
| Phosphorochloridothioic acid, O,O-diethyl ester | 10.546 | 3415327 | 0.4 |
| Phenylethyl Alcohol | 10.89 | 3765301 | 0.44 |
| Heptanedinitrile | 11.393 | 67905076 | 8 |
| 1,5-Hexadiene, 3-methyl- | 12.161 | 226811 | 0.03 |
| 2-Coumaranone | 12.78 | 347980 | 0.04 |
| Dehydroacetic Acid | 14.663 | 151420 | 0.02 |
| Pentadecane | 15.515 | 286586 | 0.03 |
| **6-pentyl-alpha-pyrone** | **16.099** | **363443330** | **42.8** |
| 2H-Pyran-2-one, 5,6-dihydro-6-pentyl- | 16.218 | 1108728 | 0.13 |
| 2,6,10,14-Hexadecatetraen-1-ol, 3,7,11,15-tetramethyl- acetate, (E,E,E)- | 17.935 | 1182914 | 0.14 |
| Sesquicineole | 19.214 | 12938873 | 1.52 |
| (1R,3E,7E,11R)-1,5,5,8-Tetramethyl-12-oxabicyclo[9.1.0]dodeca-3,7-diene | 19.821 | 3151707 | 0.37 |
| 2,3,4,5,6-Pentachloroaniline | 20.624 | 4564290 | 0.54 |
| Octadecane, 1-chloro- | 20.879 | 2048183 | 0.24 |
| 5,9,13-Pentadecatrien-2-one, 6,10,14-trimethyl-, (E,E)- | 21.4 | 4369274 | 0.51 |
| Nonadecane, 9-methyl- | 22.192 | 561954 | 0.07 |
| Dodecane, 2,6,10-trimethyl- | 22.542 | 1053163 | 0.12 |
| 3,6,9,12,15-Pentaoxanonadecan-1-ol | 23.72 | 287951 | 0.03 |
| Thunbergol | 23.839 | 1185089 | 0.14 |
| 7-Hexadecenal, (Z)- | 24.317 | 997635 | 0.12 |
| Heptadecane, 8-methyl- | 24.64 | 217963 | 0.03 |
| Hexaethylene glycol | 27.569 | 153401 | 0.02 |
| Tetrapentacontane | 29.398 | 1351660 | 0.16 |
| Ergosterol | 31.572 | 2246217 | 0.26 |

| **Supplementary Table 2.** GC-MS chromatograms of *Trichoderma atroviride* IMI206040 samples cultured in minimal medium supplemented with NO_3_ for 5 days. | | | |
| --- | --- | --- | --- |
| Name | Ret Time | Area | Area % |
| Butanoic acid, 2-methyl- | 6.579 | 54053 | 0.01 |
| 2-Heptanone | 7.114 | 116322 | 0.03 |
| Furan, 2-pentyl- | 8.782 | 20753 | 0.01 |
| 2,3-Butanediol, diacetate | 9.868 | 113198 | 0.03 |
| Phosphorochloridothioic acid, O,O-diethyl ester | 10.549 | 1020434 | 0.26 |
| Ethyl 3-acetoxybutyrate | 10.706 | 256638 | 0.06 |
| Heptanedinitrile | 11.273 | 2693168 | 0.68 |
| 1H-Benzotriazole, 5-methyl- | 11.657 | 171607 | 0.04 |
| Butanedioic acid, diethyl ester | 11.76 | 126675 | 0.03 |
| 1-Undecanol | 12.267 | 145353 | 0.04 |
| Heptane, 2,4-dimethyl- | 12.352 | 101781 | 0.03 |
| Cyclohexane, isothiocyanato- | 12.751 | 197071 | 0.05 |
| Dodecane, 4,6-dimethyl- | 13.395 | 37457 | 0.01 |
| Dehydroacetic Acid | 14.632 | 1723826 | 0.44 |
| Decane, 2-methyl- | 15.39 | 38683 | 0.01 |
| unknown | 15.669 | 1061962 | 0.27 |
| **6-pentyl-alpha-pyrone** | **15.938** | **124317909** | **31.42** |
| Octadecane, 1-iodo- | 16.637 | 123438 | 0.03 |
| unknown | 16.724 | 4812543 | 1.22 |
| Pentadecane, 2-methyl- | 16.899 | 369031 | 0.09 |
| (E,E,E)-2,6,10,14-Hexadecatetraen-1-ol acetate | 17.924 | 620339 | 0.16 |
| 1,2-Benzenediol, 3,5-bis(1,1-dimethylethyl)- | 19.076 | 796360 | 0.2 |
| unknown | 19.123 | 811825 | 0.21 |
| unknown | 19.22 | 34453248 | 8.71 |
| 1-Decanol, 2-hexyl- | 20.713 | 243172 | 0.06 |
| unknown | 21.755 | 2222263 | 0.56 |
| Octadecane, 1-chloro- | 24.309 | 859211 | 0.22 |
| Heptadecane, 8-methyl- | 26.408 | 930922 | 0.24 |
| unknown | 26.555 | 5528780 | 1.4 |
| unknown | 26.846 | 1311357 | 0.33 |
| unknown | 26.986 | 19578511 | 4.95 |
| Squalene | 28.473 | 841036 | 0.21 |
| Ergosterol | 31.838 | 3550617 | 0.9 |

| **Supplementary Table 3.** GC-MS chromatograms of *Trichoderma atroviride* IMI206040 samples cultured in minimal medium supplemented with NH_4_ for 5 days. | | | |
| --- | --- | --- | --- |
| Name | Ret Time | Area | Area % |
| Heptaethylene glycol monododecyl ether | 29.939 | 761956 | 0.34 |
| Cyclodecasiloxane, eicosamethyl- | 29.17 | 244514 | 0.11 |
| 3,5-Cyclo-6,8(14),22-ergostatriene | 29.066 | 262604 | 0.12 |
| 4,4'-Ethylenebis(2,6-di-tert-butylphenol) | 28.375 | 1042675 | 0.47 |
| 13-Docosenamide, (Z)- | 28.223 | 2803304 | 1.25 |
| Unknown | 27.508 | 3759982 | 1.68 |
| Unknown | 27.001 | 9806224 | 4.38 |
| Diisooctyl phthalate | 26.494 | 1427083 | 0.64 |
| Unknown | 26.379 | 3607877 | 1.61 |
| Unknown | 26.155 | 2509941 | 1.12 |
| Octaethylene glycol monododecyl ether | 25.831 | 927231 | 0.41 |
| Undec-10-ynoic acid, dodecyl ester | 24.344 | 1591825 | 0.71 |
| Octadecanoic acid | 23.211 | 330688 | 0.15 |
| n-Hexadecanoic acid | 21.324 | 1238074 | 0.55 |
| 9-Undecenal, 2,10-dimethyl- | 19.249 | 38565020 | 17.23 |
| 2,6-Di-t-butyl-4-methylphenol acetate(ester) | 18.56 | 2409268 | 1.08 |
| Unknown | 18.32 | 5958198 | 2.66 |
| Cyclohexene, 1-decyl- | 17.617 | 1601794 | 0.72 |
| Hexacosyl heptafluorobutyrate | 17.462 | 600260 | 0.27 |
| Globulol | 17.252 | 1837754 | 0.82 |
| trans-Geranylgeraniol | 17.069 | 638072 | 0.29 |
| Butylated Hydroxytoluene | 16.498 | 30819349 | 13.77 |
| 2,5-Cyclohexadiene-1,4-dione, 2,6-bis(1,1-dimethylethyl)- | 15.95 | 12708082 | 5.68 |
| 2-Methyltetracosane | 15.035 | 216351 | 0.1 |
| Dehydroacetic Acid | 14.661 | 464685 | 0.21 |
| 2-Hydroxy-iso-butyrophenone | 13.481 | 220663 | 0.1 |
| Nonane, 5-(2-methylpropyl)- | 13.226 | 340193 | 0.15 |
| Benzaldehyde, 2,4-dimethyl- | 12.513 | 4197125 | 1.88 |
| 2-Buten-1-ol, 2-methyl- | 11.944 | 1012183 | 0.45 |
| Ethyl 3-acetoxybutyrate | 10.739 | 244567 | 0.11 |
| Phosphorochloridothioic acid, O,O-diethyl ester | 10.578 | 405710 | 0.18 |
| 2,5-Furandione, 3,4-dimethyl- | 9.704 | 196445 | 0.09 |
| 1,3-Dioxolane-2-acetic acid, 2-methyl- | 9.499 | 218579 | 0.1 |
| 2,5-Furandione, 3-methyl- | 8.103 | 1269806 | 0.57 |
| Butanoic acid, 3-hydroxy-, ethyl ester | 7.897 | 568465 | 0.25 |
| 3-Hexanol, 2-methyl- | 6.707 | 352881 | 0.16 |

| **Supplementary Table 4**. GC–MS profiling of pyrone-related compounds by *Trichoderma* strains under different sample preparation conditions | | | | | | | | | |
| --- | --- | --- | --- | --- | --- | --- | --- | --- | --- |
| **Strain** | **Sample type** | **Compound** | **RI actual** | **RI literature** | **MS match** | **Peak area** | **Area %** | **RT (min)** |  |
| **LU497** | Amberlite resin extract * | **6-pentyl-alpha-pyrone ^1^** | 1431 | 1415 | 0.96 | **2.74E+08** | **74.91** | 14.625 |  |
|  |  | δ-decanolactone ^2^ | 1452 | 1455 | 0.94 | 3.29E+05 | 0.09 | 14.895 |  |
|  |  | (E)-6-Pent-1-enylpyran-2-one | 1483 |  | 0.82 | 1.34E+07 | 3.66 | 15.28 |  |
|  |  | δ-Undecalactone ^3^ | 1513 | 1535 | 0.83 | 6.66E+04 | 0.02 | 15.655 |  |
|  |  | tetrahydro-4-hydroxy-6-pentyl-2H-pyran-2-one ^4^ | 1630 |  | 0.87 | 3.72E+05 | 0.1 | 17.04 |  |
|  | Control supernatant extract | **6-pentyl-alpha-pyrone ^1^** | 1420 | 1415 | 0.96 | **5.11E+07** | **64.8** | 14.485 |  |
|  |  | Massoilactone ^5^ | 1431 | 1433 | 0.93 | 1.50E+05 | 0.19 | 14.63 |  |
|  |  | (E)-6-Pent-1-enylpyran-2-one | 1479 |  | 0.82 | 1.36E+06 | 1.73 | 15.235 |  |
| **LU771** | Amberlite resin extract * | **6-pentyl-alpha-pyrone ^1^** | 1438 | 1415 | 0.97 | **4.22E+08** | **78.58** | 14.71 |  |
|  |  | δ-decanolactone ^2^ | 1455 | 1455 | 0.93 | 1.75E+05 | 0.03 | 14.93 |  |
|  |  | (E)-6-Pent-1-enylpyran-2-one | 1485 |  | 0.84 | 2.31E+07 | 4.31 | 15.31 |  |
|  |  | tetrahydro-4-hydroxy-6-pentyl-2H-pyran-2-one ^4^ | 1632 |  | 0.89 | 4.02E+05 | 0.07 | 17.065 |  |
|  |  | 6-heptyl-5,6-dihydro-2H-Pyran-2-one ^6^ | 1647 | 1703.8 | 0.83 | 5.47E+05 | 0.1 | 17.225 |  |
|  | Amberlite supernatant extract | **6-pentyl-alpha-pyrone ^1^** | 1416 | 1415 | 0.97 | **4.20E+05** | **1.85** | 14.435 |  |
|  | Control supernatant extract | **6-pentyl-alpha-pyrone ^1^** | 1420 | 1415 | 0.96 | **4.30E+07** | **67.34** | 14.48 |  |
|  |  | Massoilactone ^5^ | 1432 | 1433 | 0.91 | 7.25E+04 | 0.11 | 14.635 |  |
|  |  | (E)-6-Pent-1-enylpyran-2-one | 1479 |  | 0.82 | 9.95E+05 | 1.56 | 15.235 |  |
|  |  | 6-heptyl-5,6-dihydro-2H-Pyran-2-one ^6^ | 1645 | 1703.8 | 0.83 | 1.77E+04 | 0.03 | 17.21 |  |
| **LU775** | Amberlite resin extract * | **6-pentyl-alpha-pyrone ^1^** | 1433 | 1415 | 0.96 | **3.06E+08** | **72.55** | 14.645 |  |
|  |  | (E)-6-Pent-1-enylpyran-2-one | 1483 |  | 0.83 | 1.65E+07 | 3.9 | 15.285 |  |
|  |  | tetrahydro-4-hydroxy-6-pentyl-2H-pyran-2-one ^4^ | 1632 |  | 0.91 | 9.65E+05 | 0.23 | 17.06 |  |
|  |  | 6-heptyl-5,6-dihydro-2H-Pyran-2-one ^6^ | 1646 | 1703.8 | 0.89 | 6.96E+05 | 0.17 | 17.215 |  |
|  | Amberlite supernatant extract | 2H-Pyran-2,4(3H)-dione, 3-acetyl-6-methyl- ^7^ | 1316 |  |  | 1.51E+04 | 0.11 | 13.1 |  |
|  |  | **6-pentyl-alpha-pyrone ^1^** | 1417 | 1415 | 90 | **4.39E+04** | **0.31** | 14.445 |  |
|  | Control supernatant extract | **6-pentyl-alpha-pyrone ^1^** | 1419 | 1415 | 0.96 | **3.34E+07** | **61.22** | 14.475 |  |
|  |  | Massoilactone ^5^ | 1432 | 1433 | 0.9 | 2.34E+04 | 0.04 | 14.635 |  |
|  |  | (E)-6-Pent-1-enylpyran-2-one | 1479 |  | 0.81 | 1.13E+06 | 2.06 | 15.235 |  |
| **LU1369** | Amberlite resin extract * | **6-pentyl-alpha-pyrone ^1^** | 1434 | 1415 | 0.96 | **3.37E+08** | **75.42** | 14.66 |  |
|  |  | Massoilactone ^5^ | 1440 | 1433 | 0.85 | 1.78E+06 | 0.4 | 14.735 |  |
|  |  | (E)-6-Pent-1-enylpyran-2-one | 1483 |  | 0.83 | 1.88E+07 | 4.2 | 15.285 |  |
|  |  | 6-heptyl-5,6-dihydro-2H-Pyran-2-one ^6^ | 1647 | 1703.8 | 0.94 | 2.93E+05 | 0.07 | 17.225 |  |
|  | Amberlite supernatant extract | **6-pentyl-alpha-pyrone ^1^** | 1417 | 1415 | 0.93 | **5.08E+04** | **0.32** | 14.445 |  |
|  | Control supernatant extract | 2H-Pyran-2,4(3H)-dione, 3-acetyl-6-methyl- ^7^ | 1316 |  |  | 9.25E+04 | 0.16 | 13.1 |  |
|  |  | **6-pentyl-alpha-pyrone ^1^** | 1419 | 1415 | 0.96 | **3.21E+07** | **56.93** | 14.475 |  |
|  |  | (E)-6-Pent-1-enylpyran-2-one | 1479 |  | 0.81 | 5.01E+05 | 0.89 | 15.235 |  |
| **LU132** | Control supernatant extract | **6-pentyl-alpha-pyrone ^1^** | 1420 | 1415 | 0.96 | **5.35E+07** | **65.69** | 14.49 |  |
|  |  | Massoilactone ^5^ | 1432 | 1433 | 0.93 | 1.56E+05 | 0.19 | 14.635 |  |
|  |  | (E)-6-Pent-1-enylpyran-2-one | 1479 |  | 0.82 | 1.50E+06 | 1.84 | 15.235 |  |
|  | Amberlite supernatant extract | **6-pentyl-alpha-pyrone ^1^** | 1416 | 1415 | 0.97 | **5.41E+05** | **2.76** | 14.435 |  |
| * 20X Sample dilution | |  |  |  |  |  |  |  |  |
